# Characterization of Rhamnolipids Produced by an Arctic Marine Bacterium from the *Pseudomonas fluorescence* Group

**DOI:** 10.3390/md16050163

**Published:** 2018-05-14

**Authors:** Venke Kristoffersen, Teppo Rämä, Johan Isaksson, Jeanette Hammer Andersen, William H. Gerwick, Espen Hansen

**Affiliations:** 1 Marbio, UiT—The Arctic University of Norway, N-9037 Tromsø, Norway; venke.kristoffersen@uit.no (V.K.); teppo.rama@uit.no (T.R.); jeanette.h.andersen@uit.no (J.H.A.); 2 Department of Chemistry, UiT—The Arctic University of Norway, N-9037 Tromsø, Norway; johan.isaksson@uit.no; 3 Center for Marine Biotechnology and Biomedicine, Scripps Institution of Oceanography and Skaggs School of Pharmacy and Pharmaceutical Sciences, University of California San Diego, La Jolla, CA 92093, USA; wgerwick@ucsd.edu

**Keywords:** arctic bacteria, bioactive, OSMAC (one strain, many compounds), molecular networking, rhamnolipids

## Abstract

The marine environment is a rich source of biodiversity, including microorganisms that have proven to be prolific producers of bioactive secondary metabolites. Arctic seas are less explored than warmer, more accessible areas, providing a promising starting point to search for novel bioactive compounds. In the present work, an Arctic marine *Pseudomonas* sp. belonging to the *Pseudomonas (P.) fluorescence* group was cultivated in four different media in an attempt to activate biosynthetic pathways leading to the production of antibacterial and anticancer compounds. Culture extracts were pre-fractionated and screened for antibacterial and anticancer activities. One fraction from three of the four growth conditions showed inhibitory activity towards bacteria and cancer cells. The active fractions were dereplicated using molecular networking based on MS/MS fragmentation data, indicating the presence of a cluster of related rhamnolipids. Six compounds were isolated using HPLC and mass-guided fractionation, and by interpreting data from NMR and high-resolution MS/MS analysis; the structures of the compounds were determined to be five mono-rhamnolipids and the lipid moiety of one of the rhamnolipids. Molecular networking proved to be a valuable tool for dereplication of these related compounds, and for the first time, five mono-rhamnolipids from a bacterium within the *P. fluorescence* group were characterized, including one new mono-rhamnolipid.

## 1. Introduction

It is estimated that only a small percentage of the existing marine bacterial diversity has been cultivated to date. As a result, there is a strong likelihood to isolate previously uncultured bacterial strains and some of these will produce new secondary metabolites (SMs) [1]. It is also likely to find novel SMs from already cultivated bacteria by applying the OSMAC (one strain many compounds) approach [2]. The concept behind this approach is that some metabolic pathways remain silent during standard cultivation conditions and the corresponding SMs are not synthesized. Introducing small changes into the cultivation conditions can activate different metabolic pathways which may lead to the production of numerous SMs from a single strain [2].

When searching for novel SMs from natural sources, it is important to reduce the time and resources spent on rediscovering known compounds. The process of identifying known compounds is known as “dereplication”. The most common method of dereplication in natural product (NP) drug discovery employs mass spectrometry (MS) in combination with liquid chromatography, as this combination is both sensitive and well suited for analyzing complex mixtures. Further, when using high-resolution MS, the accurate mass of the compound can be used to calculate the elemental composition which can then be used to search databases such as MarinLit, Dictionary of Natural Products, and SciFinder to identify known molecules. However, this approach will only recognize compounds that are identical to those in the databases, and any that are similar but non-identical to existing compounds will not be identified [3,4,5]. A strategy to overcome this limitation is to include information on MS/MS fragmentation in the dereplication process, as fragments will be characteristic for common structural features in a molecular class. These fragment data can be used to search MS fragment libraries such as Global Natural Products Social molecular networking (GNPS) [6]. As the number of NPs included in these fragmentation libraries is rapidly increasing, this method is becoming very useful for dereplication as well as compound class identification. The MS fragmentation data from compounds in a given sample can also be organized into molecular networks, a feature which also displays the mass differences between compounds in a network cluster. Therefore, compounds with similar structures will give similar fragmentation patterns and group together [7,8].

In the current study, we cultivated in four different media a newly isolated Arctic marine *Pseudomonas* sp. strain M10B774 that is affiliated with the *P. fluorescence* group. Fractions of the culture extracts were screened for antibacterial activity against the pathogenic bacteria *Staphylococcus aureus*, *Enterococcus faecalis*, *Streptococcus agalactiae*, *Escherichia coli* and *Pseudomonas aeruginosa* in a growth inhibition assay. Cytotoxic activity of the fractions was also evaluated against three cancer cell lines, human melanoma (A2058), human breast carcinoma (MCF7) and human colon carcinoma (HT29), as well as the non-malignant normal lung fibroblast cell line (MRC5). Further, the project demonstrated the use of MS/MS-based molecular networking as a dereplication strategy to identify known compounds, their analogs and related compounds. The use of this strategy led to the isolation of one new and four known mono-rhamnolipids as well as the lipid moiety from one of the rhamnolipids.

## 2. Results

### 2.1. Identification of the Isolate M10B774

The bacterium isolate M10B774 was isolated from an Atlantic halibut in the Norwegian Sea on a medium containing Difco Marine Broth 15 g/L, peptone 5 g/L, 300 mL filtered seawater and 700mL Milli-Q water (FMAP). To identify the bacterium, 16S rRNA sequencing and Basic Local Alignment Search Tool (BLAST) searches against reference sequences in GenBank were performed [9]. Based on these results, a set of related sequences were selected and a phylogenetic tree created (Figure S1). This phylogenetic analysis showed that the isolate is closely related to *P. gessardii* and belongs to the *P. fluorescence* group. The identity was not fully resolved, but it is suggested that the isolate is a new species or perhaps conspecific with *P. gessardii*.

### 2.2. Bioactive Extracts

The *Pseudomonas* sp. isolate was evaluated for its potential to produce antibacterial and cytotoxic compounds. It was cultivated in four different growth media: M19, VR_1, VR_2 and SGC (media compositions are listed in Section 4.2), in volumes of 2 × 200 mL. Compounds excreted into the medium were collected by adding Diaion^®^ HP20ss resin beads to the cultures. The resin was collected and extracted with methanol (MeOH). Dried extracts were fractionated with flash chromatography into six fractions and screened for antibacterial activity in a growth inhibition assay against *E. coli*, *S. aureus*, *P. aeruginosa*, *E. faecalis* and *S. agalactiae.* The fractions were screened for cytotoxic activity against three cancer cell lines, A2058, HT29 and MCF7, as well as the non-malignant MRC5 cell line, using a viability assay.

With six fractions obtained from each of the four extracts, 24 fractions were screened for bioactivity. Fraction 5, eluting in 100% methanol from the M19, VR_1 and VR_2 growth media showed activity in both the antibiotic and cytotoxicity assays. The screening results showed that cultivating this *Pseudomonas* sp. in the four different growth media led to different bioactivity profiles (Table 1). Fraction 5 from the VR_1, VR_2 and M19 media showed activity in the antibacterial assay (OD_600 nm_ < 0.05), whereas no activity was observed in the SGC fractions. The M19 Fraction 5 was active against all three of the Gram-positive bacteria, whereas the VR_2 Fraction 5 showed activity against just two of them, *S. agalactiae* and *E. faecalis*. Further, Fraction 5 from the VR_1 culture was active against only one bacterium, *S. agalactiae*. None of the tested fractions had any effect on the assayed Gram-negative bacteria (*E. coli* and *P. aeruginosa*).

In the cytotoxicity assay, only the M19 Fraction 5 was active against all the four tested cell lines (Table 1). Based on these bioactivity results, active Fraction 5 from the VR_2 media was analyzed using LC-MS/MS to generate molecular networks for the compounds present in this fraction.

### 2.3. Identification of Bioactive Compounds

A molecular network-based approach using MS/MS data from active Fraction 5 (sample VR_2) as well as the inactive Fractions 4 and 6 utilized the GNPS platform in an attempt to identify the compounds responsible for the observed antibacterial activity. The molecular networking gave rise to 183 clusters. One of the clusters was especially promising because the nodes (compounds) were exclusively present in active Fraction 5. Moreover, one of the nodes matched with that of a rhamnolipid standard that was present in the GNPS library.

Rhamnolipids are secondary metabolites that consist of one or two rhamnose moieties linked to one or two saturated or unsaturated fatty acids [10,11], and are known to have potent surfactant properties [11]. The clustering of the compounds indicated that they were likely related rhamnolipids. UHPLC-HR-ESI-MS analysis of the fraction suggested that the compounds were present as Na^+^ adducts. UHPLC-HR-ESI-MS of active Fraction 5 from the M19 and VR_1 samples revealed that the same compounds were present (i.e., identical retention times, accurate mass and collisional cross sections), whereas the inactive Fraction 5 from the SGC sample did not contain detectable amounts of any of these compounds. Based on the chromatographic and culture condition distribution of these rhamnolipids, it was suspected that they were responsible for the observed activity; resultingly, they were selected for isolation and structure elucidation.

### 2.4. Characterization of the Isolated Compounds

**Figure 1:**
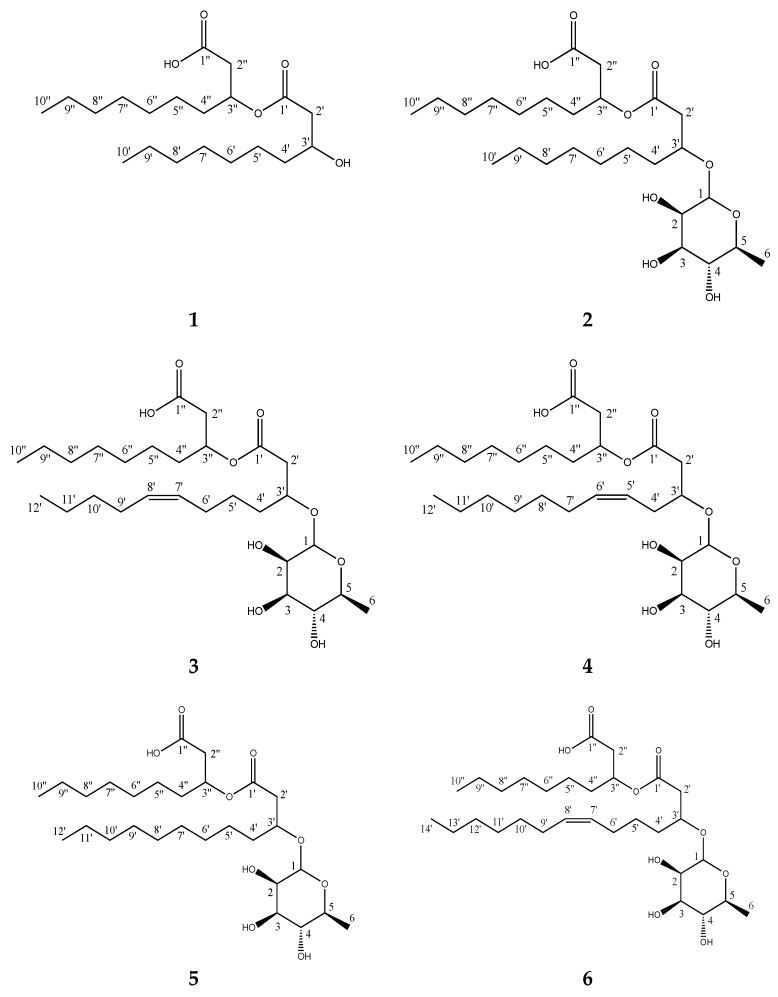
Structures of compounds **1**–**6** isolated from *Pseudomonas* sp.

Compounds **1**–**6** were isolated as viscous liquids. Their molecular formulae were calculated using accurate mass and isotope distribution from HR-ESI-MS. The structures shown in Figure 1 were determined using 1D and 2D NMR as well as and MS/MS fragmentation. Compound **1** was found to be the lipid moiety of compound **2**, and compounds **2**–**6** were found to be mono-rhamnolipids with different fatty acids. Proton scalar coupling constants, as well as chemical shifts, were in close agreement with the previously reported relative configuration of the rhamnose moiety; ^3^*J*(1,2 1.7 Hz), ^3^*J*(2,3 3.3 Hz), ^3^*J*(3,4 9.5 Hz), and ^3^*J*(4,5 9.5 Hz). The observed NOESY/ROESY patterns with two overlapping anti-phase zero quantum coherence artifacts for H3–H4 and H4–H5 suggests that extra care should be taken when interpreting these results. This conclusion is consistent with a rhamnose sugar since the zero quantum coherences (ZQCs) suggest that H4, H5 and H6 are sequentially anti and axial to each other, thus giving rise to strong scalar couplings with very little ROE contribution, but with significant ZQC due to their similar chemical shifts. Together with chemical shifts and coupling constants, all sugar moieties in **2**–**6** are fully consistent with rhamnose in α position. HSQC, HMBC, H2BC and HSQC-TOCSY were successfully employed to fully assign the resonances of the lipid chains and the positions of unsaturation.

The molecular formula of **1** was calculated to be C_20_H_38_O_5_ (*m*/*z* 381.2609, [M + Na]^+^, calcd 381.2611), suggesting two degrees of unsaturation. 1D and 2D NMR spectra (Figures S4–S8) showed that the compound was a di-lipid comprised of two-saturated 3-hydroxydecanoic acids that were linked through an ester bond. MS/MS fragmentation confirmed that each fatty acid consisted of 10 carbon atoms (Figure S46).

The molecular formula of **2**, C_26_H_48_O_9_ (*m*/*z* 527.3192, [M + Na]^+^, calcd 527.3191), indicated three degrees of unsaturation. 1D and 2D NMR data (Figures S9–S13) revealed that it was the known rhamnolipid Rha-C_10_-C_10_ [11], consisting of one rhamnose moiety with the same fully saturated C_10_-C_10_ di-lipid moiety as in **1**. The size and saturation of the lipid chains were confirmed with MS/MS fragmentation data (Figure S47).

**Figure 2:**
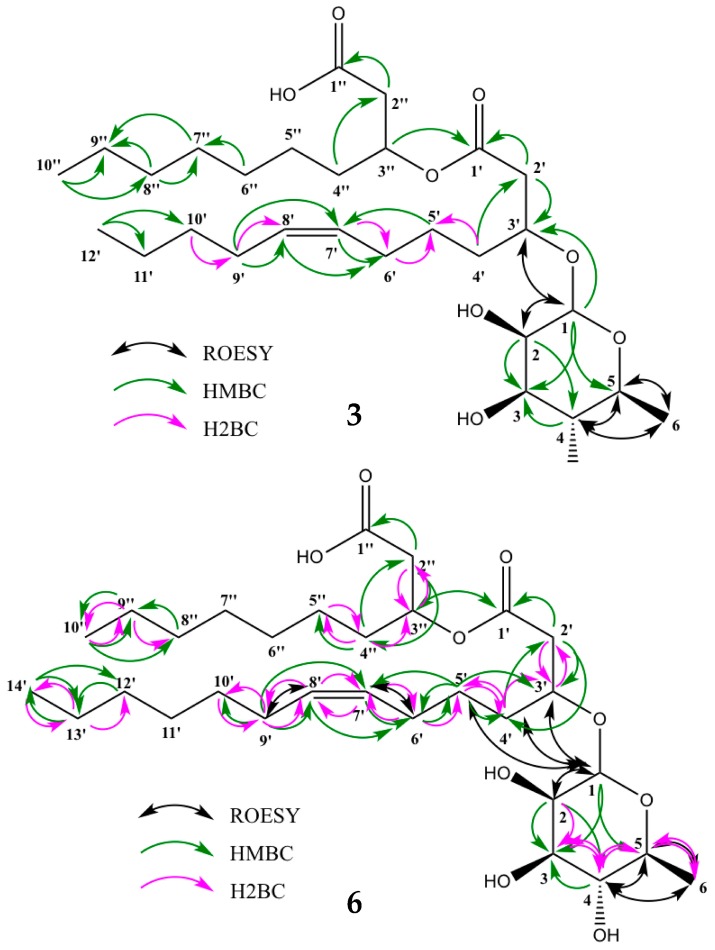
Selected 2D-NMR correlations for compound **3** and **6**. HMBC and H2BC revealed the position of unsaturation in the lipid chain, and the full lipid spin systems were identified in HSQC-TOCSY. HMBC and ROESY correlations confirmed the rhamnose moiety structure, while ROESY as well as homo- and heteronuclear coupling constants determined the olefinic protons to be in *cis* configuration.

Compound **3** had the same molecular formula as that of compound **4**, C_28_H_50_O_9_ (*m*/*z* 553.3344, [M + Na]^+^, calcd 553.3347), but a different retention time, suggesting that it had a different unsaturation pattern. 2D NMR data (HSQC + HMBC) indicated that **3** was indeed a rhamnolipid very similar to **4**, but with the double bond at position 7′–8′ instead of 5′–6′ (Figure 1). The structures of the lipid chains were assembled by HMBC and H2BC correlation data as a result of the central placement of the double bond which induced good spectral dispersion throughout the 12-carbon chain and allowed for the unambiguous identification of all carbon resonances. MS/MS fragmentation data confirmed the length of the lipid chains to be C_10_ and C_12_, with an unsaturation on the C_12_ chain (Figure S48). The assignments are summarized in Table 2 and Figure 2. The configuration of the olefinic protons of compound **3** could not be directly assessed because of spectral overlaps in both the proton and the carbon dimensions for 7′/8′ as well as 6′/9′. It is reported here in a *cis* configuration by analogy to the other rhamnolipids isolated in this work. See below for the determination of the configuration of compounds **4** and **6**.

Compound **4**, which was recently isolated and identified from *Pseudomonas* sp. [12], was assigned the molecular formula C_28_H_50_O_9_ (*m*/*z* 553.3348 [M + Na]^+^, calcd 553.3347). 1D and 2D NMR (Figures S19–S26), together with MS/MS fragmentation (Figure S49), confirmed the lipid chains to be 10 and 12 carbon atoms long, with the unsaturation present in the C_12_ chain at position 5′–6′ (Figure 1). Upon closer examination, it was found that the configuration of the olefinic protons was in a *cis* configuration, which is in disagreement to what has been previously reported [12]. The vicinal ^3^*J*_HH_ coupling constant between the two vinyl protons was determined to be roughly 10.9 Hz from deconvolution and simulation of the 1D proton multiplets (dtt, *J* = 10.9, 7.3, 1.5 Hz) (Figure S27). The ROE between the two protons has a dominant antiphase character (Zero Quantum artifact) and is close to the diagonal, making it inconclusive as it could be present in both configurations. However, a ROE/NOE connectivity can be traced from 4′→5′→6′→7′ as well as a direct 4′→7′ consistent with *cis* (Figure S26). Furthermore, there are no direct ROE/NOE from 4′→6′ or 7′→5′, which would have been expected in a *trans* configuration. The ^3^*J*_CH_ couplings involving the olefinic protons were estimated to be between 9–10 Hz which also favors a *cis* configuration over *trans* (Figures S28 and S29).

The molecular formula of compound **5**, C_28_H_52_O_9_ (*m*/*z* 555.3503, [M + Na]^+^, calcd 555.3504), indicated structural similarity to **3** and **4**, but without the unsaturation on one of the lipid chains, as it had one less degree of unsaturation. 1D and 2D NMR (Figures S30–S34) as well as MS/MS fragmentation (Figure S50) confirmed it was a Rha-C_10_-C_12_, hence, the same lipid chain lengths as **3** and **4**, but fully saturated. A database search revealed that it was a known compound, previously identified from *Pseudomonas aeruginosa* [13].

The molecular formula of **6** was determined to be C_30_H_54_O_9_ (*m*/*z* 581.3660, [M + Na]^+^, calcd 581.3660), indicating four degrees of unsaturation and one lipid chain two carbons longer than compounds **3**, **4** and **5**. The four degrees of unsaturation indicated that one lipid chain possessed a double bond. 1D and 2D NMR data (Figures S35–S43) established **6** to be the mono-rhamnolipid Rha-C_14:1_-C_10_, with the unsaturation at position 7′–8′. From MS/MS fragmentation (Figure S51), the lipid chain lengths were confirmed to be 10 and 14 carbons long, with the unsaturation being present in the longer chain. A database search showed that rhamnolipids with the composition Rha-C_14:1_-C_10_ are indeed known, but neither the position of unsaturation nor the order of the lipid chains were assigned in the previous studies [11]. However, comparing the NMR and MS/MS fragmentation data with the data from Tedesco et al. [12], it seems probable that their compound 3 has the same structure as our compound **6**. Our 1D and 2D NMR data were nearly identical to that reported, with the mean error of carbon chemical shifts = 0.69 ppm. Furthermore, the MS fragmentation data showed an identical pattern. However, they interpreted their data differently and described a different structure (Rha-C_12:1_-C_12_). We believe that the key fragment at *m*/*z* 265.18 represents the sodium adduct of the first fatty acid (i.e., 1′–14′) which indicates that the two lipid chains are C_10_ and C_14_ instead of both being C_12_. This is in agreement with the fragmentation mechanism of compounds **3**, **4**, **5** and **6**. The difference in mass of the fragments between **6** and **3**, **4**, and **5** correspond to C_2_H_4_, suggesting that the additional C_2_H_4_ is added to the unsaturated chain instead of the saturated chain as Tedesco et al. reported for their compound 3. Simulations in Mass Frontier 7.0 were not conclusive as both tentative structures of **6** could form fragments of the correct mass within a reasonable number of steps. Careful examination of the HSQC-TOCSY data for **6** allowed us to unambiguously identify all 14 carbons in the spin system of the suggested unsaturated lipid chain (Figures S41 and S42), thus conclusively establishing the identity of the rhamnolipid with two chains of 10 and 14 carbons, respectively, and where the longer chain possesses a double bond at position 7′–8′. The assignments are summarized in Table 3 and Figure 2. Analogous to compound **3**, the configuration of the olefinic protons was found to be in a *cis* configuration. The vicinal ^3^*J*_HH_ coupling constant between the two nearly overlapping olefinic protons was determined to be roughly 10.9 Hz from deconvolution and simulation of the 1D proton multiplets (dtt, *J* = 10.9, 6.6, 0.6 Hz) (Figure S15). The ROE/NOE pattern is less dispersed because of the greater distance to the branching point, but careful inspection allowed us to identify that all observable correlations did indeed follow the same pattern as in compound **4** (Figure S44). Most importantly there are no direct ROE/NOE from 4′→6′ or 7′→5′, which would have been expected in a *trans* configuration.

### 2.5. Bioactivity of Compounds **1**–**6**

#### 2.5.1. Antibacterial Activity

**Figure 3:**
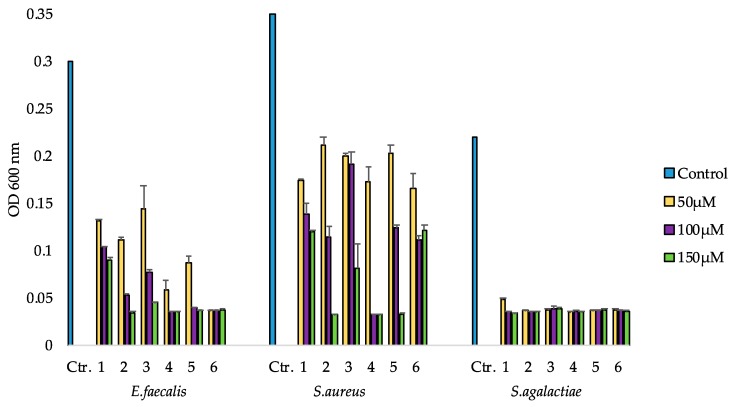
Growth inhibition assay of **1**–**6** tested at three concentrations against the Gram-positive bacteria *E. faecalis*, *S. aureus* and *S. agalactiae*. Bacteria and medium (50:50) were used as negative growth controls. Values are means of two replicates, error bars indicate standard deviation.

The six isolated compounds were tested for antibacterial activity in a growth inhibition assay and in a biofilm formation inhibition assay. Test concentrations in both bioassays were 50, 100 and 150 µM. In the growth inhibition assay, the compounds were tested against five pathogenic bacteria. All of the compounds were active against the three Gram-positive bacteria (Figure 3); however, none showed activity against the two Gram-negative bacteria *E. coli* and *P. aeruginosa* (Figure S2). Compounds **1**–**5** also showed a dose dependent activity against *E. faecalis*. Compared to the control, **1** had some effect at all three test concentrations, but it was less active than the other five compounds. Compound **2** was highly active (OD_600 nm_ ≤ 0.05) against *E. faecalis* at the two highest concentrations, while compound **3** showed high activity against *E. faecalis* only at the highest concentration of 150 µM. Compounds **4** and **5** were highly active at the two highest concentrations, while **6** had high activity at all three concentrations.

Against *S. aureus,* all compounds displayed a dose dependent activity. Compounds **1**, **3** and **6** had some effect at all concentrations compared to the control, but they did not show a high level of activity even at 150 µM. Compounds **2** and **5** were highly active at 150 µM, and **4** was active at the two highest concentrations. All compounds were highly active against *S. agalactiae* from 50 µM.

#### 2.5.2. Inhibition of Biofilm Formation

**Figure 4:**
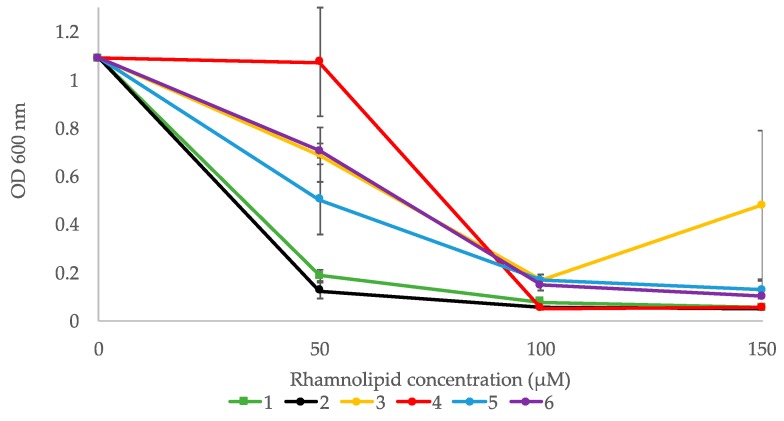
Biofilm formation inhibition assay performed on *S. epidermidis*. Values are mean of three replicates, ± standard error.

The ability of the six compounds to inhibit biofilm formation was tested using the Gram-positive bacterium *Staphylococcus epidermidis*. All compounds displayed a dose dependent activity (Figure 4). Compounds **1** and **2** displayed high activity with OD_600 nm_ values below 0.2 (controls had OD ~1.0) at 50 µM, whereas the other compounds had high activity at 100 µM and above (Figure 4). Compound **3** seemed to have higher effect at 100 µM compared to 150 µM, but that is likely due to variations in the assay.

#### 2.5.3. Cytotoxic Activity

**Figure 5:**
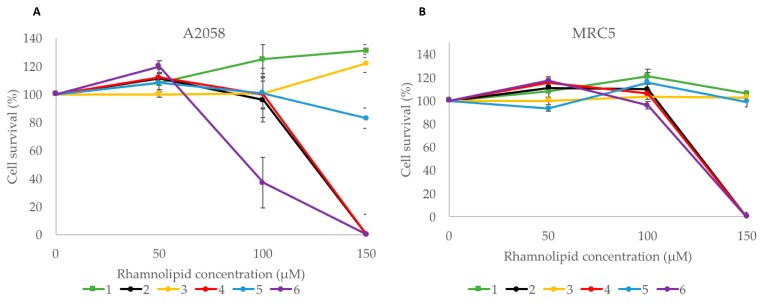
A cell viability MTS assay was used to evaluate the cytotoxicity of compounds **1**–**6**: (**A**) Human melanoma cells (A2058); (**B**) non-malignant cells (MRC5). Values are mean of three replicates, ± standard error.

The human melanoma cancer cell line A2058 and the non-malignant MRC5 cell line were used to test compounds **1**–**6** for activity in an MTS cell viability assay (Figure 5). Compounds **2**, **4** and **6** showed a dose-dependent activity against A2058 cells. They had no effect at the lowest concentration, but compound **6** had some activity at 100 µM, with around 40% cell survival. At 150 µM, compounds **2**, **4** and **6** showed high activity with 0% cell survival. Compounds **1**, **3** and **5** did not display any activity against the A2058 cells. While compounds **2**, **4** and **6** also displayed activity against MRC5 cells at 150 µM, with 0% cell survival, compounds **1**, **3** and **5** showed no effect against this cell line at the tested concentrations.

## 3. Discussion

This newly isolated *Pseudomonas* sp. strain was cultured in four different media, and the culture extracts were subsequently fractionated into six fractions each prior to bioactivity screening. SMs are often produced in small quantities, and other compounds, such as media components and primary metabolites, can mask their activities. This risk is mitigated when the extracts are pre-fractionated, which generally increases the hit rate in bioassays [11]. The bioactivity screening of the fractions from the four media revealed that the selected media influenced the production of bioactive compounds. Activity was observed in Fraction 5 from the M19, VR_1 and VR_2 media. These are all nutrient rich media wherein the main difference is the energy source, a feature which is known to affect the production of secondary metabolites [11,14]. The M19 medium has d-mannitol as the energy source, and Fraction 5 from this medium showed activity against all three of the tested Gram-positive bacterial strains. In addition, it was the only fraction that showed activity in the cancer cell viability assay, where it was active against all three of the cancer cell lines as well as the non-malignant cell line. The fractions deriving from the extracts formed from growth in the VR_1 and VR_2 media were similar; both media contain yeast and malt extracts as energy source. The difference between them is that the VR_2 medium contains iron sulfate and potassium bromide, which are components of seawater [15]. Adding trace elements to a growth medium is known to effect the production of secondary metabolites [16], and this modification seemed to have some effect in our study, as the VR_2 Fraction 5 was active against both *E. faecalis* and *S. agalactiae*, whereas the VR_1 Fraction 5 was active only against *S. agalactiae* in the antibacterial assay. No samples from the SGC medium had any activity in the bioactivity screening. This was the only low nutrient medium used; we had hypothesized that stressing the *Pseudomonas* sp. might induce the production of new secondary metabolites. As the samples from this growth medium did not have any activity, it may be that the nutrient level was too low to allow the production of energetically costly antibacterial and anticancer compounds. These results demonstrate that a diverse selection of growth media is important when searching for bioactive compounds from cultured microorganisms.

HR-ESI-MS analyses of the fractions showed that the isolated rhamnolipids were present in the samples from the M19, VR_1 and VR_2 media, but not in the inactive SGC sample. Rhamnolipids are known to have antibacterial and cytotoxic activities, so these compounds were suggested and later confirmed to be responsible for the observed bioactivity [17]. Yield, diversity and ratios of rhamnolipids depend on cultivation conditions [18,19,20], so differences in the rhamnolipid content and composition due to different media composition can explain why the three samples were active in the different bioassays. However, it is also possible that non-identified compounds were responsible for some of the observed bioactivity. The effect of the media composition was clearly observed for compound **1**, as it was among the most abundant peaks in the HR-ESI-MS of the M19 extract, while it was found only in minute amounts in the VR_1 and VR_2 extracts.

MS/MS fragmentation followed by molecular networking proved to be an effective way to dereplicate these related rhamnolipids. Using HR-ESI-MS for dereplication of bioactive compounds is a powerful tool, as the elemental composition can be used to search databases of known compounds. However, subtle changes in the chemical structure of a known compound can be difficult to recognize, such as position of unsaturation and relative carbon chain length of fatty acid chains. Using MS/MS fragmentation patterns to establish relationships between molecules within a sample as well as between unknown compounds and library references can facilitate the dereplication process.

The molecular network cluster also suggested that the VR_2 Fraction 5 contained di-rhamnolipids. From HR-ESI-MS analysis, it appeared that the di-rhamnolipids had the same retention time as the mono-rhamnolipids with the same lipid chains, the only difference being an extra rhamnose moiety in the di-rhamnolipidc (e.g., Rha-Rha-C_10_-C_10_, and Rha-C_10_-C_10_). The same feature was observed in the prep-HPLC-MS data obtained during isolation of the mono-rhamnolipids from the M19 extracts; it appeared that the mono-rhamnolipids and traces of the corresponding di-rhamnolipids had the same retention times. However, when analyzing the purified compounds by NMR, di-rhamnolipids were not detected. This suggests that the di-rhamnolipids were likely generated in the ion source of the MS. Rhamnose moieties are easily removed from the lipid moiety in the ion source, resulting in free rhamnose moieties which can react with a mono-rhamnolipid, forming a di-rhamnolipid species. Indeed, considering the structural differences of mono- and di-rhamnolipids, they are not expected to have the same retention times. Déziel et al. [21] and Behrens et al. [22] showed that mono-rhamnolipids and the corresponding di-rhamnolipids had different retention times on reversed-phase HPLC columns, supporting the idea that the proposed di-rhamnolipids were generated in the ion source.

Rhamnolipids were first discovered in 1946 by Bergstrøm et al. [23] as a product of *P. aeruginosa*. Subsequently, other *Pseudomonas* sp. and bacteria from the genus *Burkholderia* have been discovered to produce rhamnolipids, but the known producers are still limited to only a few species [11,24,25]. Rhamnolipids have been widely studied, and today more than 60 congeners and isomers have been identified and characterized, as reviewed by Abdel-Mawgoud et al. in 2010 [11]. In addition to having antibacterial and cytotoxic activity, rhamnolipids have also shown antiviral, antifungal and anti-biofilm activities. Most studies have focused on *P. aeruginosa*, which is currently used for the industrial production of rhamnolipids. However, one issue arising from use of this bacterium for commercial production is its human pathogenicity [26,27,28]. Bacteria from the *P. fluorescence* group are not known to be human pathogens, so the *Pseudomonas* sp. strain used in this study could be a candidate to replace *P. aeruginosa* for industrial production of rhamnolipids. Hence, it is important to gain insight into which rhamnolipids this M10B744 strain produces. 

The *Pseudomonas* sp. strain M10B744 was partly identified by phylogenetic analysis of the 16S rRNA gene, and is either a *P. gessardii*, or a new species closely related to *P. gessardii. P. gessardii* is not well studied, but *P. fluorescence* and *P. synxantha*, belonging to the *P. fluorescence* group, are reported to produce rhamnolipids [29,30,31,32,33]. However, the only rhamnolipid structurally characterized from this group is the di-rhamnolipid Rha-Rha-C_10_-C_10_ isolated from a *P. fluorescence* strain [34]. Thus, the five mono-rhamnolipids we isolated in the current study are the first mono-rhamnolipids structurally characterized from the *P. fluorescence* group. 

In this study, we were able to describe the fatty acids and their order for all the isolated rhamnolipids, including the position and stereochemistry of the double bonds. However, the absolute stereochemistry of C-3′ and C-3″ remains unresolved. The structure of compound **3** is described for the first time in this study. Searches in databases indicate that it is a new compound. Rhamnolipids with the same elemental composition and lipid chain lengths have been reported in several studies [21,22,35], but without the position of unsaturation or order of lipid chains identified. The previously reported structures are not necessarily identical to **3**, as it contains an unsaturation that in principal can be present in different positions. This is illustrated for compound **4** which had the same elemental composition and lipid chain lengths as **3**, C_10_–C_12:1_, but with the unsaturation at a different position (Figure 2). Compound **4** was recently described by Tedesco et al. as an isolate from an Antarctic *P. aeruginosa* [12]. 

We identified compound **6** as a mono-rhamnolipid with lipid chains C_10_ and C_14:1_. Rhamnolipids with these chains have previously been reported, but the position of the unsaturation and order of chain lengths have not been previously assigned [36]. However, comparison of our NMR and MS/MS fragmentation data with data from the study by Tedesco et al. revealed that the data were identical, and that compound **6** is the same rhamnolipid as their compound 3, which they described as a novel rhamnolipid with C_12_ and C_12:1_ lipid chains. MS/MS analysis of **6** gave a key fragment at *m*/*z* 411.24, and this mass corresponds the loss of a C_10_ lipid chain (Figure S51). Although this fragment was also present in the data of Tedesco et al., it was not assigned to any specific loss. In conclusion, both the NMR data (Figures S35–S45) and the MS/MS data (Figure S51) strongly indicated that the lipid chains are C_10_ and C_14:1_, and not C_12_ and C_12:1_ as reported by Tedesco et al. [12].

Much of the previous bioactivity screening of rhamnolipids has been performed on mixtures or on non-characterized rhamnolipids [37,38,39,40,41,42]. In the current study, we assessed the bioactivity of these natural products individually, and tested the isolated compounds in their pure form. In the antibacterial assay, all compounds showed some effect against the three Gram-positive bacteria strains. However, no activity was observed against the two Gram-negative bacteria strains, which usually are less sensitive to antimicrobial agents due to their outer cell wall that contains lipopolysaccharides acting as an extra barrier [43]. All isolated compounds were active in the biofilm formation inhibition assay against Gram-positive *S. epidermidis*.

A number of antimicrobial agents are amphiphilic compounds, such as daptomycin [44] and brilacidin [45], that function by binding to membranes as detergents, leading to membrane lysis. Rhamnolipids are amphiphilic due to their lipophilic lipid chain and hydrophilic rhamnose moiety, and are reported to act by affecting the membrane of target cells [46,47]. Sotirova et al. [48] found that rhamnolipids are inserted into the phospholipid membrane of cells, thus affecting their structure and function, which can lead to cell death. Al-Tahhan et al. [49] reported that rhamnolipids lead to the loss of lipopolysaccharides (LPS) and subsequent alteration of the outer membrane in the Gram-negative bacterium *P. aeruginosa*. Jiang et al. [50] reported that rhamnolipids can also induce cytotoxicity by reducing the surface tension of the culture medium, and this is also an effect of their amphiphilic nature [51,52].

As the rhamnose moiety is the same for all five of the mono-rhamnolipids studied herein, the variations in bioactivity between these compounds must be a result of differences in the lipid chains. The difference in activity in the cytotoxicity assay between **3** and **5** (not active) and **4** (highly active with 0% cell survival for both A2058 and MRC5) is somewhat surprising. Compounds **3**, **4** and **5** are structurally very similar to one another, as they have the same lipid chain lengths, C_10_-C_12_, but **3** and **4** have an unsaturation at different positions in chain B, and **5** is fully saturated. On the other hand, it is possible that there are some inaccuracies in the test concentrations, a matter that should be considered when working with small amounts of isolated natural products.

The effect of the rhamnose moiety was seen when comparing the activity of **1** and **2**, as they had the same lipid moiety but **2** also contained a rhamnose moiety. Fatty acids are known to have surfactant activity and to exhibit antibacterial activity by affecting the membrane of cells [53,54]. This was verified in the antibacterial assays, as **1** was active in both the growth inhibition and anti-biofilm assays, similar to the rhamnolipids, indicating that the presence of a rhamnose moiety in compound **2** did not substantially enhance the antibacterial activity. However, in the viability assays, compound **1** did not show any activity, whereas **2** was active against both cell lines; thus, it is clear that including a rhamnose moiety had an effect on the activity against the human A2058 and MRC5 cells.

In conclusion, using different cultivation media for the *Pseudomonas* sp. strain M10B744 gave extracts with different bioactivity profiles, appearently due to changes in the production of rhamnolipids. The rhamnolipids were initially identified by the use of MS/MS fragmentation data and molecular networking, demonstrating the utility of this approach for dereplication. Five mono-rhamnolipids were characterized for the first time from a bacterium within the *P. fluorescence* group. One of the rhamnolipids was a new molecule, demonstrating that Arctic marine bacteria can be a valuable resource for new bioactive molecules.

## 4. Materials and Methods

### 4.1. Microorganism

Isolation: *Pseudomonas* sp. strain M10B774, was isolated from an Atlantic halibut (*Hippoglossus hippoglossus*) in the Norwegian Sea, dd° N 77,46707333 and dd° E 10,609719 in January 2010. It was streaked onto FMAP agar consisting of: 15 g Difco marine broth (279110, Becton, Dickinson and Company, Franklin Lakes, NJ, USA), 15 g agar (A1296, Sigma-Aldrich, St. Louis, MO, USA), 700 mL Milli-Q water (Merck Millipore, Darmstadt, Germany), 300 mL filtrated seawater (FSW, 5 μm pore size, ceramic membrane filter 0.2 μm, UV filter) and 5 g peptone from caseine (82303, Sigma-Aldrich). After isolation the strain was stored in FMAP broth (without agar) and 30% glycerol (G5516, Sigma-Aldrich) at −80 °C. 

Identification: The isolate was stored at −80 °C, plated on FMAP agar plate and grown at 10 °C for 7 days before a single colony was inoculated into an Eppendorf tube with 100 μL of Milli-Q and boiled for 5 min. PCR was performed on a thermal cycler (Mastercycler epgradient S, Eppendorf, Hamburg, Germany) using 1 μL of the bacterial lysate as template, 1 μM of forward primer (27F, AGAGTTTGATCMTGGCTCAG), 1 μM of reverse primer (1492R, CGGTTACCTTGTTACGACTT) and 12.5 μL of ThermoPrimeTM 2× ReddyMix PCR master mix (ThermoFisher Scientific, Waltham, MA, USA) in a total volume of 25 μL. PCR was carried out using the following program: 94 °C for 5 min, 30 cycles at 94 °C for 30 s, 55 °C for 30 s, and 72 °C for 1 min, followed by a final extension at 72 °C for 10 min. The PCR products were analyzed by electrophoresis on a 1.0% agarose gel and documented with Bioimaging system, Syngene. The PCR product of 16S rRNA gene was purified with QIAquick PCR purification kit according to the manufacturer′s instructions (QIAGEN, Hilden, Germany). The primers 27F or 1492R were employed to sequence the purified PCR product. Sequence data were collected by the sequencing lab at University Hospital of North Norway (Tromsø, Norway). Homology searches were performed using the Basic Local Alignment Search Tool (BLAST) provided by the NCBI server (http://www.ncbi.nlm.nih.gov/BLAST) and the strain was identified using phylogenetic interference. See detailed description of the identification process in Supplementary Information Figure S1.

### 4.2. Fermentation and Extraction of Secondary Metabolites

*Pseudomonas* sp. was grown in 2 × 1 L Erlenmeyer flasks at 10 °C at 140 rpm in 200 mL M19, VR_1, VR2 and SGC medium (Table 4). All medium components were from Sigma-Aldrich, except Iron (II) sulfate heptahydrate (FeSO_4_·7H_2_O) and potassium bromide (KBr) from Merck. SGC medium were suspended in 100% FSW, whereas the three other media were in 50:50 FSW and Milli-Q.

The bacterium was cultivated in the four different media until growth was visible (1–2 weeks). To collect secondary metabolites excreted into the medium, Diaion^®^HP-20 resin beads (13607, Supelco Analytica, Bellefonte, PA, USA), 40 g/L, which were soaked in MeOH (34860, Sigma-Aldrich) for 20 min and washed extensively in Milli-Q water, were added to the cultures 3–4 days before extraction. Extraction was performed by filtrating the cultures under vacuum, using a fine mesh cheesecloth (1057, Dansk Hjemmeproduktion, Ejstrupholm Danmark)). Resin beads captured on the cheesecloth were washed with 100 mL Milli-Q and extracted twice with 150 mL MeOH before vacuum filtered through Whatman Ø 90 mm No. 3 filter (Whatman plc, Buckinghamshire, UK). The extracts were dried under pressure and stored at −20 °C.

### 4.3. Fractionation

Extracts of *Pseudomonas* sp. cultivated in the four media were dissolved in 8 mL 90% MeOH. Then, 2 g Diaion^®^ HP-20ss resin beads were added before the mixture was dried under pressure. Resin (6.5 g) was soaked in MeOH for 20 min before being exchanged with Milli-Q water and packed in a flash cartridge (Biotage^®^ SNAP Ultra, Biotage, Uppsala, Sweden). The cartridge was equilibrated in 5% MeOH before the extract/resin mixture was loaded on top. Fractionation was performed using a Biotage SP4^TM^ system with flow rate 12 mL/min and gradient 5–100% MeOH over 32 min, and MeOH:acetone (34850, Sigma-Aldrich) to 100% acetone over 18 min. This resulted in six fractions that were dried under pressure at 40 °C.

### 4.4. Bioactivity

#### 4.4.1. Growth Inhibition Assay

Media used in the growth inhibition assay include Muller Hinton broth (MH, 275730, Becton, Dickinson and Company) and Brain Heart Infusion broth (BHI, 53286, Sigma-Aldrich). Bacteria strains that were cultured in MH medium included *S. aureus* (ATCC 25923), *E. coli* (ATCC 259233) and *P. aeruginosa* (ATCC 27853), and in BHI medium included *E. faecalis* (ATCC 29122) and *S. agalactiae* (ATCC 12386). Fresh bacteria colonies were inoculated in respective growth medium and incubated overnight at 37 °C. The number of cells was adjusted in fresh medium to reach the log phase, and added to a 96-well microtiter plate (734-2097, Nunclon^TM^, Thermo Scientific) with 1500–15,000 CFU/well, total volume 100 µL/well. Flash fractions in the primary screening were dissolved in Milli-Q water with 1% dimethyl sulfoxide (DMSO, D4540, Sigma-Aldrich) to 1 mg/mL and tested in duplicates at concentrations 50 µg/mL. The isolated compounds **1**–**6** were dissolved in Milli-Q water with 1% DMSO and added to the wells in duplicates, at the final concentrations 50 µM, 100 µM and 150 µM. The plate was incubated overnight at 37 °C before the growth was measured my assessing the absorbance for at 600 nm with 1420 Multilabel Counter VICTOR^3^_TM_ (Perkin Elmer, Waltham, MA, USA). Bacterium suspension diluted with water (1:1) was used as growth control. A dilution series of gentamycin from 32 to 0.01 µg/mL were used as positive assay controls; the growth medium was used as a negative growth control.

#### 4.4.2. Biofilm Inhibition Assay

*Staphylococcus epidermidis* (ATCC 35984) grown in Tryptic Soy Broth (TSB, 105459, Merck, Kenilworth, NJ, USA) overnight at 37 °C was diluted in fresh medium with 1% glucose (D9434, Sigma-Aldrich) before being transferred to a 96-well microtiter plate; 50 µL/well were incubated overnight with 50 µL of compound **1**–**6** dissolved in Milli-Q water added in duplicates. The bacteria were then removed from the plate and the plate washed with tap water. The biofilm was fixed at 65 °C for 1 h before 70 µL 0.1% crystal violet (115940, Merck Millipore) was added to the wells for 10 min of incubation. Excess crystal violet solution was then removed and the plate dried for 1 h at 65 °C. Seventy microliters of 70% EtOH were then added to each well and the plate incubated on a shaker for 5–10 min. Biofilm formation inhibition were assessed by the presence of violet color and was measured at 600 nm absorbance using a 1420 Multilabel Counter VICTOR^3^_TM_. Fifty microliters of a non-biofilm forming *Staphylococcus haemolyticus* (clinical isolate 8-7A, University hospital, UNN, Tromsø, Norway) mixed in 50 µL autoclaved Milli-Q water was used as a control; 50 µL *S. epidermidis* mixed in 50 µL autoclaved Milli-Q water was used as the control for biofilm formation; and 50 µL TSB with 50 µL autoclaved Milli-Q water was used as a medium blank control.

#### 4.4.3. Cytotoxicity Assay

Cell viability of fractions and pure compounds was tested in an MTS in vitro cell proliferation assay against three cancer cell lines; human melanoma A2058 (ATCC, CRL-1147^TM^), human breast carcinoma MCF7 (ATCC HTB-22^TM^) and human colon carcinoma HT29 (ATCC HTB-22^TM^) and one non-malignant cell line, normal lung fibroblasts MRC5 (ATCC CCL-171^TM^). The cells were seeded in a 96-well microtiter plate in Roswell Park Memorial Institute (RPMI-1640 medium, FG1383, Merck) with 10% Fetal Bovine serum (FBS, S0115, Biochrom, Cambridge, UK) at a concentration of 2000 cells/well for the three cancer cell lines and 4000 cells/well for MRC5. After incubation for 24 h at 37 °C and 5% CO_2_, the medium was replaced with fresh RPMI-1640 medium which included 10% FBS and gentamycin (10 µg/mL. A2712, Merck). The samples were added in triplicate, fractions at a concentration of 50 µg/mL, and isolated compounds **1**–**6** at concentrations of 50, 100 and 150 µM, to form a total volume of 100 µL/well. After an additional 72 h incubation at 37 °C and 5% CO_2_, 10 µL CellTiter 96^®^AQ_ueous_ One Solution Reagent (G3581, Promega) with tetrazolium compound [3-(4,5-dimethylthiazol-2-yl)-5-(3-carboxymethoxyphenyl)-2-(4-sulfophenyl)-2H-tetrazolium, inner salt] and phenazine ethosulfate were added to each well before incubation for 1 additional hour. The absorbance was measured at 485 nm with a DTX 880, and cell viability calculated. RPMI-1640 with 10% FBS and 0.5% Triton^TM^ X-100 (Sigma-Aldrich) were used negative controls.

### 4.5. Dereplication, Isolation and Structure Elucidation

#### 4.5.1. LC-MS/MS and Molecular Networking

LC-MS/MS data for molecular networking were obtained with a system consisting of a Thermo Finnigan Surveyor Autosampler Plus, LC-Pump-Plus and PDA Plus coupled a Thermo Finnigan LCQ Advantage Max mass spectrometer. The flash chromatography fractions were dissolved in MeOH to a concentration of 1 mg/mL, and 20 µL of each fraction was injected onto a Kinetex C18 column (5 µm, 4.6 mm × 100 mm) (Phenomenex, Torrance, CA, USA). The mobile phase consisted of acetonitrile (ACN) and H_2_O (both containing 0.1% formic acid) with a flow of 0.7 mL/min, and the components were eluted with the following gradient: 30% ACN for 5 min, increase to 99% ACN over 17 min, hold at 99% ACN for 4 min. The MS was run in positive electrospray, and data from *m*/*z* 190 to 2000 was recorded with automated full dependent MS/MS scan enabled. The chromatograms were converted to .mzxml files using msConvert (www.proteowizard.sourceforge.net), and the chromatograms were submitted to GNPS for analysis (www.gnps.ucsd.edu). Cytoscape 3.6.0 (www.cytoscape.org) was used to visualize the molecular networks. A cosine value of 0.7 was used to generate the molecular network.

#### 4.5.2. HR-MS/MS

High-resolution mass spectrometry was run with ESI+ ionization using UPLC-QToF-MS. It was performed on an Acquity UPLC I-class and a Vion IMS QToF with an Acquity UPLC C18 column (1.7 µm, 2.1 mm × 100 mm) (all from Waters). The samples were run with a 12 min gradient increasing from 10% to 90% acetonitrile (ACN, 75-05-08, Merck) with 1% formic acid (FA, 069141, Biosolve, Dieuze, France) in ultra pure water (7732, Merck) and a flow rate 0.45 mL/min. Waters UNIFI 1.8.2 Scientific Information System software was used to process the data.

#### 4.5.3. Isolation of Compounds **1**–**6**

Purification of the rhamnolipids was performed using a prep-HPLC system (Waters) consisting of a 600 HPLC pump, a 3100 mass spectrometer, a 2996 photo diode array detector and a 2767 sample manager. The system was controlled with MassLynx version 4.1. Various columns were used (all from Waters): X-Terra RP-18 Prep Column (10 µM, 10 mm × 300 mm), Atlantis Prep dC18 Column (10 µM, 10 mm × 250 mm), XSelect CSH Prep Fluoro-Phenyl (5 µM, 10 mm × 250 mm). Gradients were optimized using Milli-Q water with 0.1% FA (33015, Sigma-Aldrich) and acetonitrile (34851, Sigma-Aldrich) with 0.1% FA as mobile phase. Flow rate was constant at 6 mL/min. Flash Fraction 5 was resuspended in 100% MeOH, and the initial separation of the rhamnolipids was done on the Atlantis dC18 column using a gradient from 50% to 100% ACN over 15 min. The combinations of gradients and columns used for the final isolation of each compound are listed in Table 5.

#### 4.5.4. NMR

All NMR spectra were acquired on a Bruker Avance III HD spectrometer equipped with an inverse detected TCI probe with cryogenic enhancement on ^1^H, ^2^H and ^13^C, operating at 599.90 MHz and 150.86 MHz for ^1^H and ^13^C, respectively. Samples were prepared in DMSO-*d*_6_ and methanol-*d*_4_, and recorded at 298 K.

All experiments were acquired using standard pulse sequences for Proton, Presat, Carbon, DQFCOSY, ECOSY, HSQC (bip), HMBC (bip), H2BC (bip), HSQCTOCSY (mlev), TOCSY (clean mlev), NOESY and ROESY (adiabatic) in Topspin 3.5pl7, using gradient selection where applicable, and processed in Mnova 12.0.0. Spectra were referenced on the residual solvent peak of methanol-*d*_4_ (δ_H_ = 3.31 and δ_C_ = 49.00) or DMSO-*d*6 (δ_H_ = 2.50 and δ_C_ = 39.52).

## Figures and Tables

**Table 1:** The antibacterial activities of chromatography Fraction 5 (eluting with 100% MeOH) from the extracts of *Pseudomonas* sp. grown in four different media were tested in a growth inhibition assay. Cytotoxic activities of the fractions were evaluated with a cell viability assay. Test concentration for both assays was 50 µg/mL.

	Growth Inhibition Assay					Viability Assay			
Media	*E. coli* ^N^	*S.aur* ^P^	*P.aer* ^N^	*E.F* ^P^	*S.aga* ^P^	A2058	MCF7	HT29	MRC5 *
VR_1	−	−	−	−	+	−	−	−	−
VR_2	−	−	−	+	+	−	−	−	−
M19	−	+	−	+	+	+	+	+	+
SGC	−	−	−	−	−	−	−	−	−

Antibacterial assay: +, OD_600 nm_ < 0.05 and active; −, OD_600 nm_ > 0.05 and inactive. ^P^ Gram-positive; ^N^ Gram-negative. *S.aur*, *S. aureus*; *P.aer*, *P. aeruginosa*; *E.F*, *E. faecalis*; *S.aga*, *S. agalactiae.* Viability assay: +, >50% cell death; −, <50% cell death. * Non-malignant cell line.

**Table 2:** ^1^H and ^13^C NMR assignments for compound **3** and the observed HMBC and H2BC correlations (^1^H→^13^C).

Position	δ_C_, Type	δ_H_ (*J* in Hz)	COSY	HMBC	H2BC	ROESY
1	98.7, CH	4.62, s		3′,3,5		3′,2,4′
2	70.3, CH	3.52 ^o^		3,4		
3	69.3, CH	3.41 ^o^	4		4	
4	71.9, CH	3.12, t (9.1)	3,5	10,5	5	5
5	69.0, CH	3.43 ^o^	4,6	4	6	3′,4,6
6	17.8, CH_3_	1.08, d (6.1)	5	4,5	5	4,5
1′	170.3, C					
2′	40.1, CH_2_		3′	1′,3′	3′	3′
3′	72.9, CH	3.91, d (5.6)	2′,4′	1′	2′	2′,1,4′
4′	32.1, CH_2_	1.45, dt (10.0, 6.4)	5′,3′	2′,3′	5′	^o^
5′	24.3, CH_2_	1.30 ^o^	6′,4′	7′		^o^
6′	26.3, CH_2_	1.98 ^o^	7′,5′	8′,7′	7′,5′	^o^
7′	129.9, CH	5.33 ^o^	6′	9′,6′	8′,6′	^o^
8′	129.3, CH	5.32 ^o^	9′	9′,6′	9′,7′	^o^
9′	26.6, CH_2_	2.00 ^o^	10′,8′	11′,10′,8′,7′	10′,8′	^o^
10′	31.3, CH_2_	1.27 ^o^	9′		9′	^o^
11′	21.7, CH_2_	1.27 ^o^	12	12′,10′	12′	^o^
12′	13.8, CH_3_	0.86, t (6.9)	11′	11′,10′	11′	^o^
1″	170.6 *, C					
2″	40.4, CH_2_	2.38 ^o^		1″		
3″	71.0, CH	5.11, s ^b^	2″,4″	1′		
4″	33.7, CH_2_	1.52, s ^b^	3″	2″		^o^
5″	24.7, CH_2_	1.20 ^o^				^o^
6″	28.6 **, CH_2_	1.23 ^o^		7″		^o^
7″	28.8 **, CH_2_	1.23 ^o^		9″		^o^
8″	31.2, CH_2_	1.22 ^o^		9″,7″		^o^
9″	22.1, CH_2_	1.25 ^o^	10″	10″,8″	10″	^o^
10″	14.0, CH_3_	0.85, t (7.0)	9″	9″,8″	9″	^o^

* Not detectable in 1D, extracted from 2D HMBC ** Assignments could not be unambiguously distinguished ^b^ Broad peak ^o^ Overlapping peak in ^1^H.

**Table 3:** ^1^H and ^13^C NMR assignments for compound **6** and the observed HMBC and H2BC correlations (^1^H→^13^C).

Position	δ_C_. type		δ_H_ (*J* in Hz)	COSY	HMBC	H2BC	R/NOESY
1	99.8, CH		4.78, d (1.5)	2	3′		2,3′,4′,5′
2	72.8, CH		3.74, dd (3.3, 1.7)	1,3			1, **
3	72.0, CH		3.67 ^o^	2,4	2,4	2,4	**
4	74.2, CH		3.31 ^o^	3,5	5,6	3,5	**
5	70.1, CH		3.67 ^o^	4,6	6	4,6	**
6	17.9, CH_3_		1.25, d (6.1)	5	4	5	4,5
1′	172.8, C				2′		
2′	41.2, CH_2_		2.56, dd (15.1, 7.6)2.47 ^o^	3′	4′ ^w^	3′	3′,4′
3′	74.7, CH		4.11, dq (7.5, 5.6)	2′,4′	2′,5′	2′,4′	1,2′,4′,5′
4′	33.5, CH_2_		1.56 ^o^	3′,5′	2′,5′	5′	1,2′,3′,5′,6′
5′	25.9, CH_2_		1.30, qd (7.4, 1.5)	4′,6′	6′	4′,6′	1,3′,4′,6′
6′	28.1, CH_2_		2.05 ^o^	5′,7′	5′,(7′,8′)	7′	4′,5′,7′w,7′
7′	130.3, CH		5.34 ^o^	6′	5′,(6′,9′)	6′,8′	6′
8′	131.5, CH		5.37 ^o^	9′	(6′,9′),10′ ^w^	7′,9′	9′
9′	28.2, CH_2_		2.07 ^o^	8′,10′	(7′,8′)	8′,10′	8′,10′,11′
10′	30.8, CH_2_		1.31 ^o^	9′	9′	9′	9′
11′	30.1, CH_2_		1.32 ^o^				9′
12′	32.99, CH_2_^*^		1.30 ^o^		14′	13′	
13′	23.7, CH_2_^*^		1.31 ^o^	14′	12′,14′	14′	14′
14′	14.45, CH_3_^*^		0.91, t (7.0) *	13′	13′	13′	13′
1″	177.1, C				2″		
2″	42.3, CH_2_		2.47 ^o^	3″	4″ ^w^	3″	3″,4″
3″	73.6, CH		5.31, m	2″,4″	2″	2″,4″	2″
4″	35.4, CH_2_		1.61, q (6.6)	3″,5″	2″	5″	2″,5″
5″	26.3, CH_2_		1.33 ^o^	4″	4″	4″	4″
6″	30.6, CH_2_		1.31 ^o^				
7″	30.4, CH_2_		1.31 ^o^				
8″	32.95, CH_2_ *		1.28 ^o^		10″	9″	
9″	23.7, CH_2_ *		1.31 ^o^	10″	8″,10″	10″	10″
10″	14.46, CH_3_ *		0.90, t (7.0) *	9″	9″	9″	9″

* Assignments could not be chain-specifically distinguished; ^o^ overlapping peak in ^1^H; shift extracted from 2D HMBC; ^w^ weak.

**Table 4:** Components of growth media used for fermentation of *Pseudomonas* sp. with product numbers. The amounts of medium ingredients are given in g/L.

Medium	d-Mannitol (63560)	Peptone (82303)	d-Glucose (D9434)	Casein Hydrolase (22090)	Malt Extract (70167)	Yeast Extract (Y1625)	FeSO_4_ ⋅ 7H_2_O (1.03965)	KBr (22186)
M19	20	20	-	-	-	-	-	-
VR_1	-	11.11	-	-	6.67	6.67	-	-
VR_2	-	11.11	-	-	6.67	6.67	0.044	0.044
SGC	-	-	4	3	-	-	-	-

**Table 5:** Column, gradient and run-time used for isolation of compound **1**–**6**.

Compound	Column	Gradient (%) ACN	Time (min)
**1**	XSelect	55–57	7.00
**2**	Atlantis	70–78	10.00
**3**	Atlantis	68–72	10.00
**4**	Atlantis	70–80	12.30
**5**	X-Terra	70–78	10.00
**6**	Atlantis	80–96	12.00

## Supplementary Materials

The following are available online at http://www.mdpi.com/1660-3397/16/5/163/s1. Figures S1–S45, including a phylogenetic three of *Pseudomonas* sp., strain M10B774, bioactivity data as well as 1D and 2D NMR data, Figures S46–S51, MS/MS data of compounds **1**–**6**, and HR-ESI-MS spectra of compounds **1**–**6** can be found online.
