# Supplementary material for: Characterization of Rhamnolipids Produced by an Arctic Marine Bacterium from the Pseudomonas fluorescence Group
Source: Mar Drugs. 2018 May 14;16(5):163. doi: 10.3390/md16050163 (PMC5983294; doi:10.3390/md16050163)
Supplement: Supplementary file 1 [file marinedrugs-16-00163-s001.pdf]

Supplementary Materials

Characterization of rhamnolipids produced by an Arctic marine bacterium from the *Pseudomonas fluorescence* group

Venke Kristoffersen [1], Teppo Rämä[1], Johan Isaksson [2], Jeanette Hammer Andersen[1], William H. Gerwick[3] and Espen Hansen[1]*

[1] Marbio, UiT - The Arctic University of Norway, N-9037 Tromsø, Norway; venke.kristoffersen@uit.no (V.K.); teppo.rama@uit.no (T.R.); jeanette.h.andersen@uit.no (J.H.A.); espen.hansen@uit.no (E.H.)

[2] Department of Chemistry, UiT - The Arctic University of Norway, N-9037 Tromsø, Norway; johan.isaksson@uit.no

[3] Center for Marine Biotechnology and Biomedicine, Scripps Institution of Oceanography and Skaggs School of Pharmacy and Pharmaceutical Sciences, University of California San Diego, La Jolla, CA 92093 USA; wgerwick@ucsd.edu

* Correspondence: espen.hansen@uit.no; Tel.: +47 77649262

Table of contents

Identification of Arctic marine bacterium

Figure S1. Phylogenetic tree of *Pseudomonas* species, showing the placement of the strain M10B774.

Antibacterial screening

Figure S2. Antibacterial growth assay of compounds **1-6** against Gram-negative bacteria.

Tabulated NMR data

Table S3. ^{13}C NMR assignments for compounds **1-6**

*NMR data of compound **1**.*

Figure S4. 1D proton of **1** in DMSO-d_6, T=298 K.
Figure S5. 1D carbon of **1** in DMSO-d_6, T=298 K.
Figure S6. 2D superimposed ^{13}C-HSQC and HMBC of **1** in DMSO-d_6, T=298 K.
Figure S7. 2D DQF-COSY of **1** in DMSO-d_6, T=298 K.
Figure S8. 2D ROESY (300 ms) of **1** in DMSO-d_6, T=298 K.

NMR data for compound **2**.

Figure S9. 1D proton of **2** in methanol-d_4, T=298 K.
Figure S10. 1D carbon of **2** in methanol-d_4, T=298 K.
Figure S11. 2D superimposed ^{13}C-HSQC and HMBC of **2** in methanol-d_4, T=298 K.
Figure S12. 2D DQF-COSY of **2** in methanol-d_4, T=298 K.
Figure S13. 2D ROESY (300 ms) of **2** in methanol-d_4, T=298 K.

*NMR data for compound **3**.*

Figure S14. 1D proton of **3** in DMSO-d_6, T=298 K.
Figure S15. 1D carbon of **3** in DMSO-d_6, T=298 K.
Figure S16. 2D superimposed ^{13}C-HSQC and HMBC of **3** in DMSO-d_6, T=298 K.
Figure S17. Blown up aliphatic region of superimposed 2D ^{13}C-HSQC and HMBC of **3** in DMSO-d_6, T=298 K.
Figure S18. Blown up aliphatic region of superimposed 2D ^{13}C-HSQC and H2BC of **3** in DMSO-d_6, T=298 K.

*NMR data for compound **4**.*

Figure S19. 1D proton of **4** in DMSO-d_6, T=298 K.
Figure S20. 1D carbon of **4** in DMSO-d_6, T=298 K.
Figure S21. 2D superimposed ^{13}C-HSQC and HMBC of **4** in DMSO-d_6, T=298 K.
Figure S22. 2D ^{13}C-HSQCTOCSY of **4** in DMSO-d_6, T=298 K.
Figure S23. Blown up aliphatic region of the 2D ^{13}C-HSQCTOCSY of **4** in DMSO-d_6, T=298 K.
Figure S24. Blown up aliphatic region of superimposed 2D ^{13}C-HSQC and H2BC of **4** in DMSO-d_6, T=298 K.

Figure S25. 2D DQF-COSY of **4** in DMSO-d_6, T=298 K.
Figure S26. 2D ROESY (300 ms) of **4** in DMSO-d_6, T=298 K.
Figure S27. Comparison between experimental and simulated multiplets of the olefinic protons of **4**.
Figure S28. $^3J_{CH}$ couplings estimated using a selective CLIP-HSQMBC experiment.
Figure S29. $^3J_{CH}$ couplings estimated using a SJS-HSQC experiment

NMR data for compound ***5****.*

Figure S30. 1D proton of **5** in DMSO-d_6, T=298 K.
Figure S31. 1D carbon of **5** in DMSO-d_6, T=298 K.
Figure S32. 2D superimposed ^{13}C-HSQC and HMBC of **5** in DMSO-d_6, T=298 K.
Figure S33. 2D DQF-COSY of **5** in DMSO-d_6, T=298 K.
Figure S34. 2D ROESY (300 ms) of **5** in DMSO-d_6, T=298 K.

NMR data for compound ***6****.*

Figure S35. 1D proton of **6** in methanol-d_4, T=298 K.
Figure S36. 1D carbon of **6** in methanol-d_4, T=298 K.
Figure S37. 2D superimposed ^{13}C-HSQC and HMBC of **6** in methanol-d_4, T=298 K.
Figure S38. Blown up aliphatic region of superimposed 2D ^{13}C-HSQC and H2BC of **6** in methanol-d_4, T=298 K.
Figure S39. Blown up aliphatic region of superimposed 2D ^{13}C-HSQC and HSQC-TOCSY of **6** in methanol-d_4, T=298 K.
Figure S40. 2D ^{13}C-HSQC and HSQC-TOCSY at noise level of **6** in methanol-d_4, T=298 K.
Figure S41. 2D DQF-COSY of **6** in methanol-d_4, T=298 K.
Figure S42. 2D ROESY (300 ms) of **6** in methanol-d_4, T=298 K.
Figure S43. Blown up cyclic region of 2D ROESY (300 ms) of **6** in methanol-d_4, T=298 K.
Figure S44. Expansion of the nearly overlapped olefinic protons in a 2D NOESY (500 ms) of **6**.
Figure S45. Experimental and simulated multiplets of the nearly overlapped olefinic protons of **6**.

MS/MS data of compounds ***1****-****6****.*

Figure S46. MS/MS spectrum of **1** $[M + Na]^+$. Ion mode ESI+.
Figure S47. MS/MS spectrum of **2** $[M + Na]^+$. Ion mode ESI+.
Figure S48. MS/MS spectrum of **3** $[M + Na]^+$. Ion mode ESI+.
Figure S49. MS/MS spectrum of **4** $[M + Na]^+$. Ion mode ESI+.
Figure S50. MS/MS spectrum of **5** $[M + Na]^+$. Ion mode ESI+.
Figure S51. MS/MS spectrum of **6** $[M + Na]^+$. Ion mode ESI+.

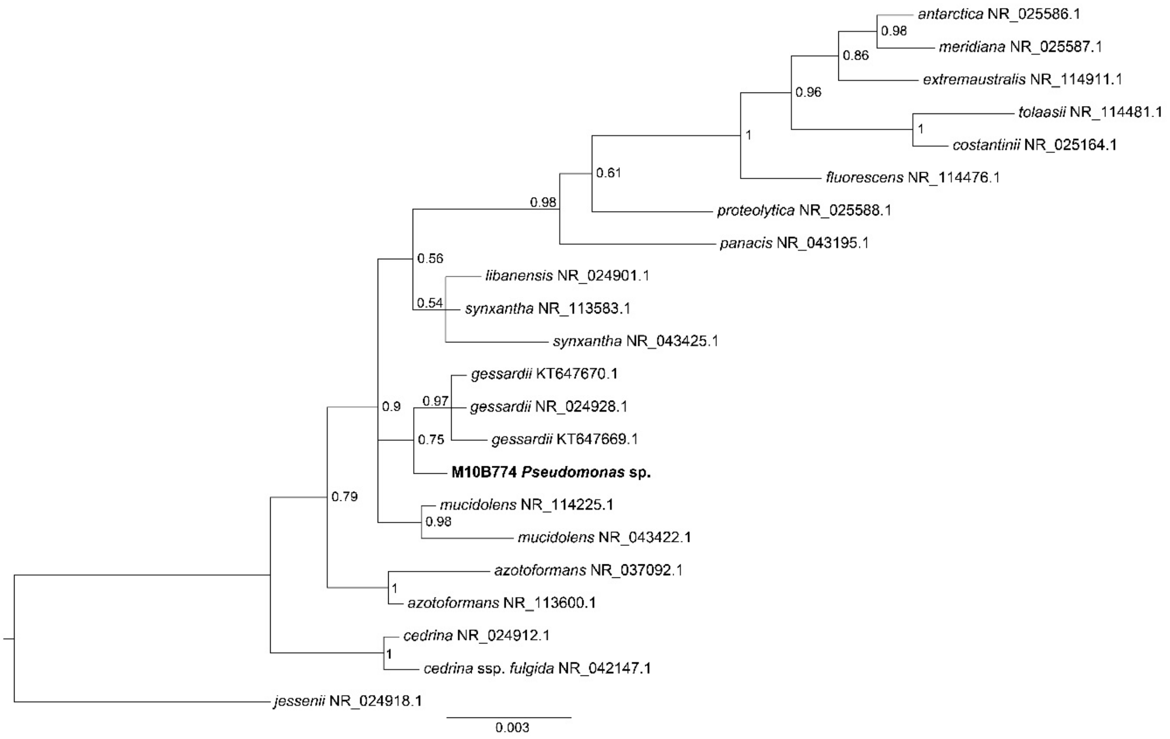

Figure S1. Phylogenetic tree of *Pseudomonas* species in the *P. fluorescence* group based on 16S rRNA gene sequences. The studied isolate M10B774 is shown in bold. Branch labels represent identification and accession codes of the Pseudomonas sequences in GenBank, node support is given as posterior probabilities (PP). *P. jessenii* was used as an outgroup for the other taxa. The accurate identity and placement of M10B774 remains unresolved, because of the adjacent polytomy and lack of node support at nearby nodes of the tree. However, the study isolate seems to be most closely related, if not conspesific, with *P. gessardii.*

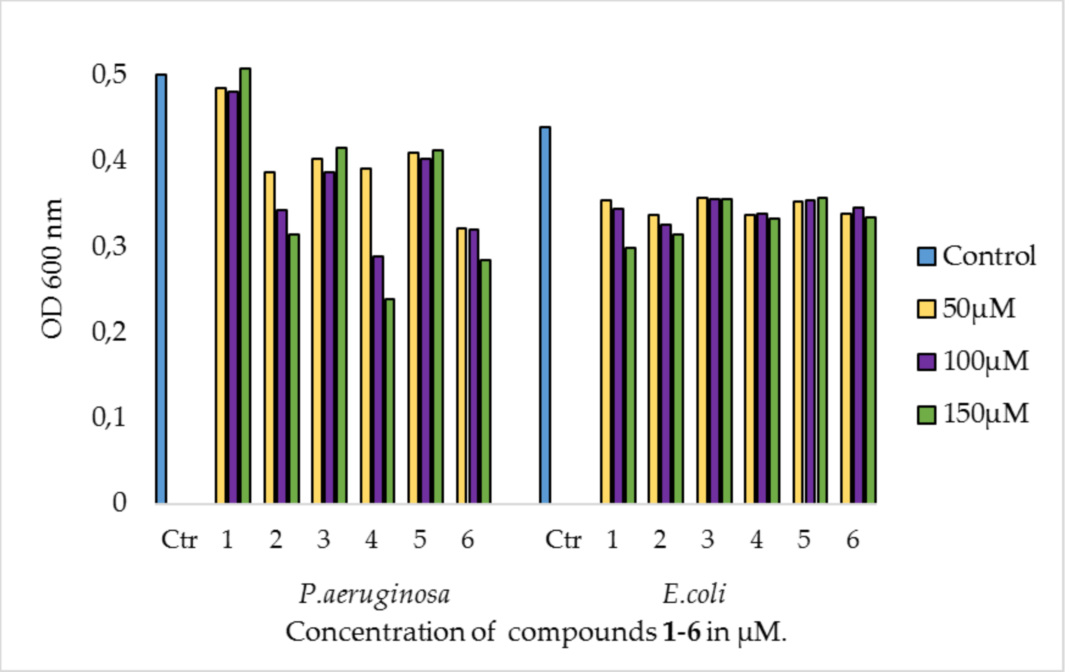

Figure S2. Antibacterial growth inhibition assay of compounds **1-6** tested at three concentrations against Gram-negative *P. aeruginosa* and *E.coli*. Bacteria cells and medium (50:50) was used as negative control (0µM). Values are mean of two parallels.

Table S3. ^{13}C NMR assignments for compounds **1-6**.

	**1** (dmso-d_6)	**2** (methanol-d_3)	**3** (dmso-d_6)	**4** (dmso-d_6)	**5** (dmso-d_6)	**6** (methanol-d_3)
1		100.62	98.7	98.75	98.69	99.82
2		72.29	70.3	70.54	70.97	72.79
3		72.19	69.3	70.47	70.31	72.03
4		74.04	71.9	71.85	71.87	74.24
5		70.00	69.0	68.76	68.93	70.14
6		17.79	17.8	17.77	17.83	17.92
1′	170.59	172.31	170.3	170.03	170.32	172.77
2′	42.76	41.18	40.1	39.54	40.06	41.25
3′	67.06	75.45	72.9	72.47	72.94	74.73
4′	36.69	34.25	32.1	30.38	32.47	33.51
5′	24.49	25.78	24.3	124.37	24.66	25.89
6′	28.70*	30.58	26.3	132.34	28.63*	28.08
7′	28.70*	30.25*	129.9	26.80	28.71*	130.29
8′	31.28*	32.86*	129.3	28.73	28.84*	131.46
9′	22.10*	23.59*	26.6	26.80	28.98*	28.22
10′	13.94*	14.32*	31.3	31.17*	31.22*	30.83
11′			21.7	22.09*	22.11*	30.08
12′			13.8	13.94*	13.96*	32.99*
13′						23.73*
14′						14.46*
1′′	171.62	174.27	170.6	171.65	172.56	177.07
2′′	38.81	39.88	40.4	38.79	40.43	42.31
3′′	69.97	72.60	71.0	70.73	71.39	73.59
4′′	33.36	34.96	33.7	33.29	33.61	35.40
5′′	24.97	26.11	24.7	24.54	24.11	26.33
6′′	28.70*	30.30	28.6	28.37	28.99*	30.56
7′′	28.70*	30.18*	28.8	28.55	29.07*	30.36
8′′	31.19*	32.82*	31.2	31.19*	31.30*	32.95*
9′′	22.10*	23.58*	22.1	22.08*	22.10*	23.73*
10′′	13.94*	14.32*	14.0	13.93*	13.95*	14.45*

* Carbon resonances are not chain specifically assigned due to near identical shifts

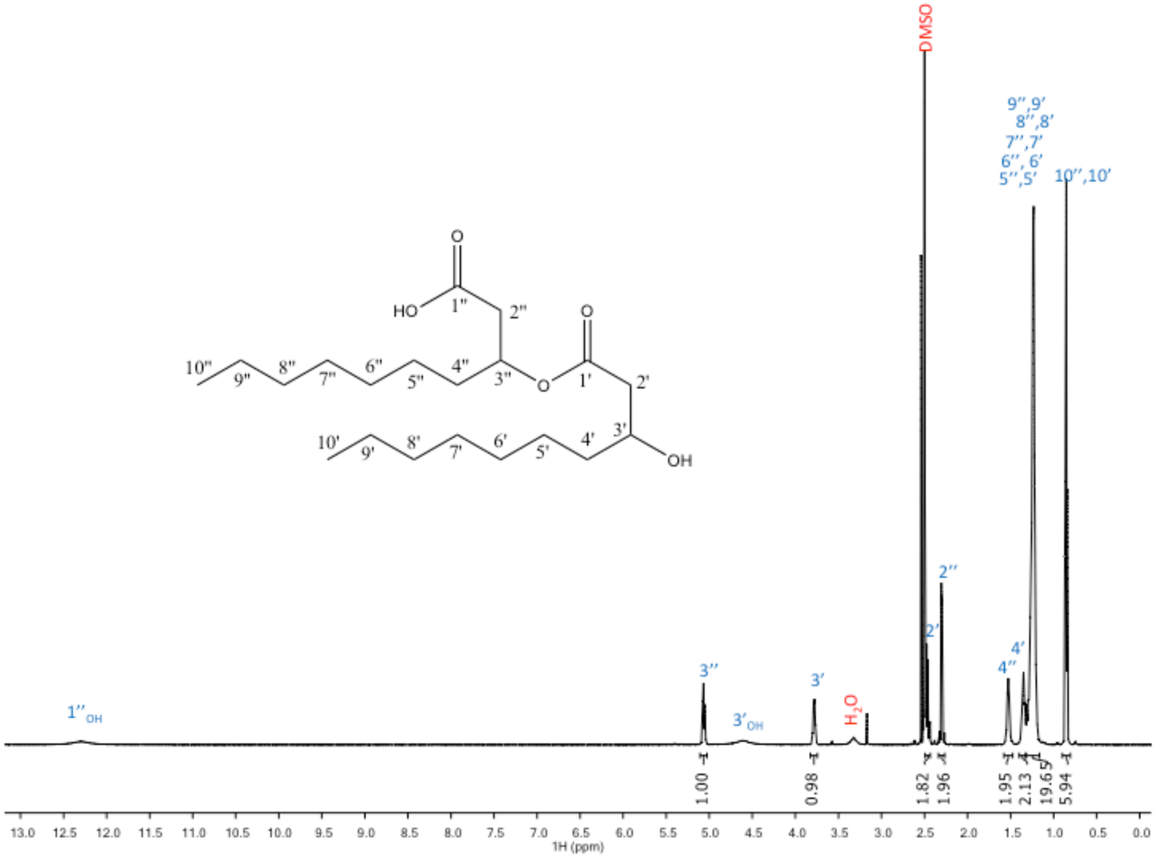

Figure S4. 1D proton of **1** in DMSO-d6, T=298 K.

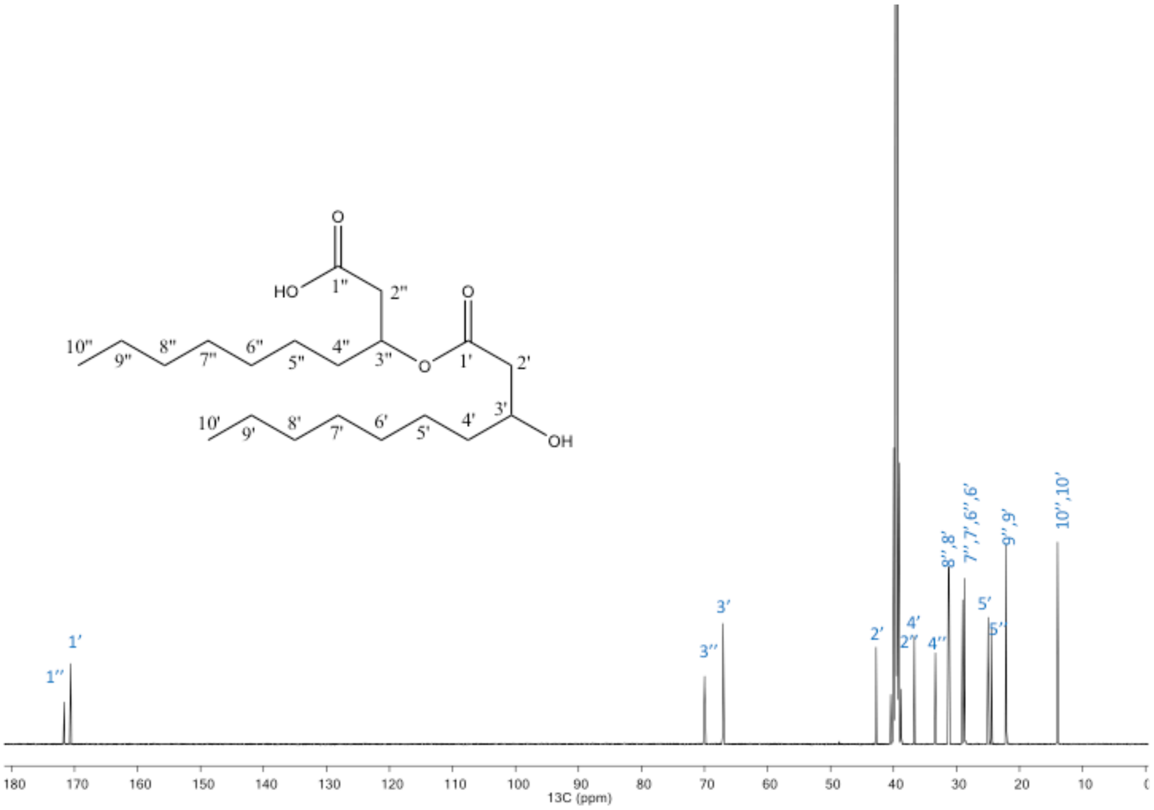

Figure S5. 1D carbon of **1** in DMSO-d6, T=298 K.

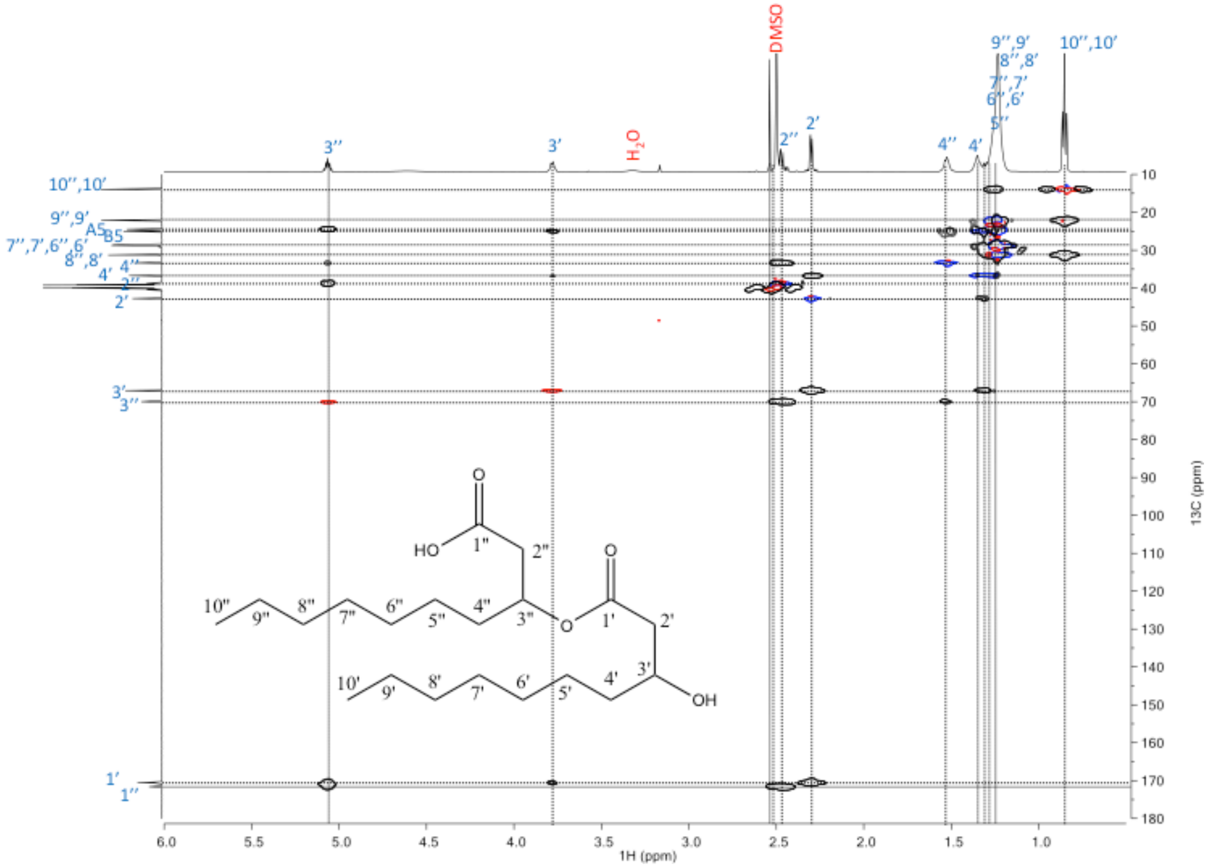

Figure S6. 2D superimposed ^{13}C-HSQC and HMBC of **1** in DMSO-d$_6$, T=298 K.

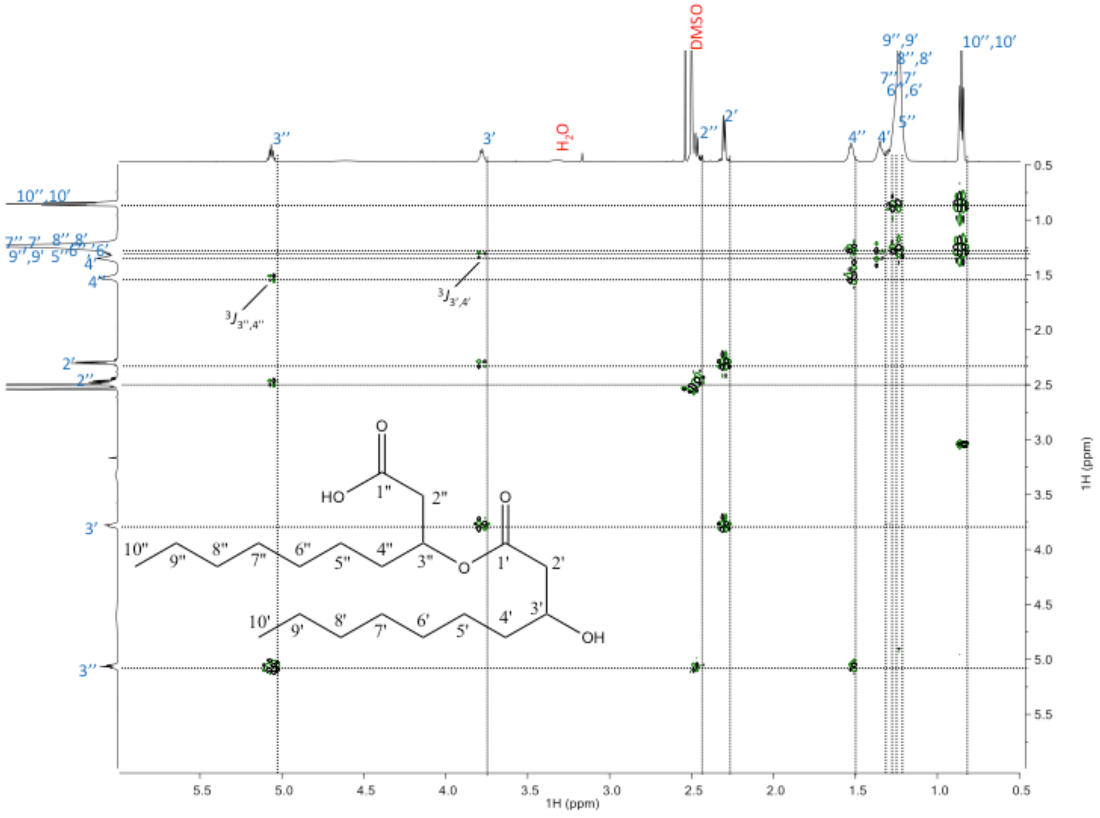

Figure S7. 2D DQF-COSY of **1** in DMSO-d$_6$, T=298 K.

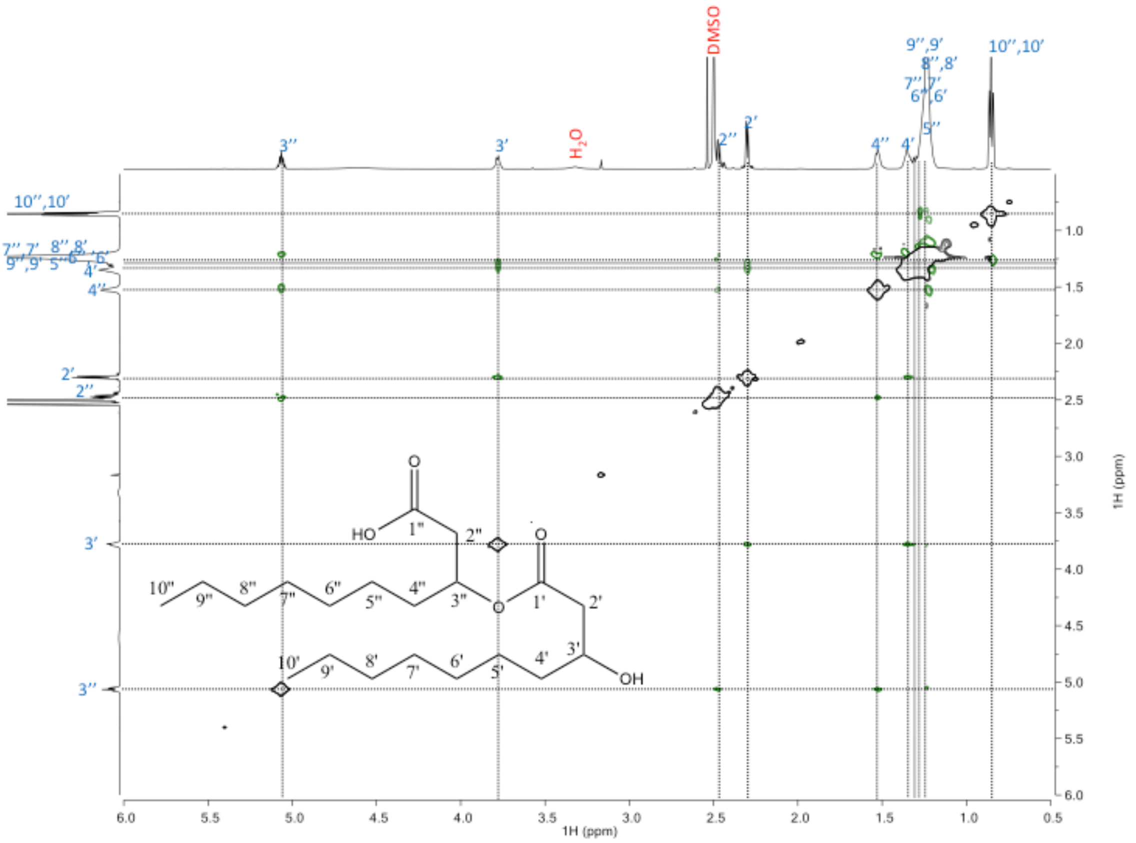

Figure S8. 2D ROESY (300 ms) of **1** in DMSO-d6, T=298 K.

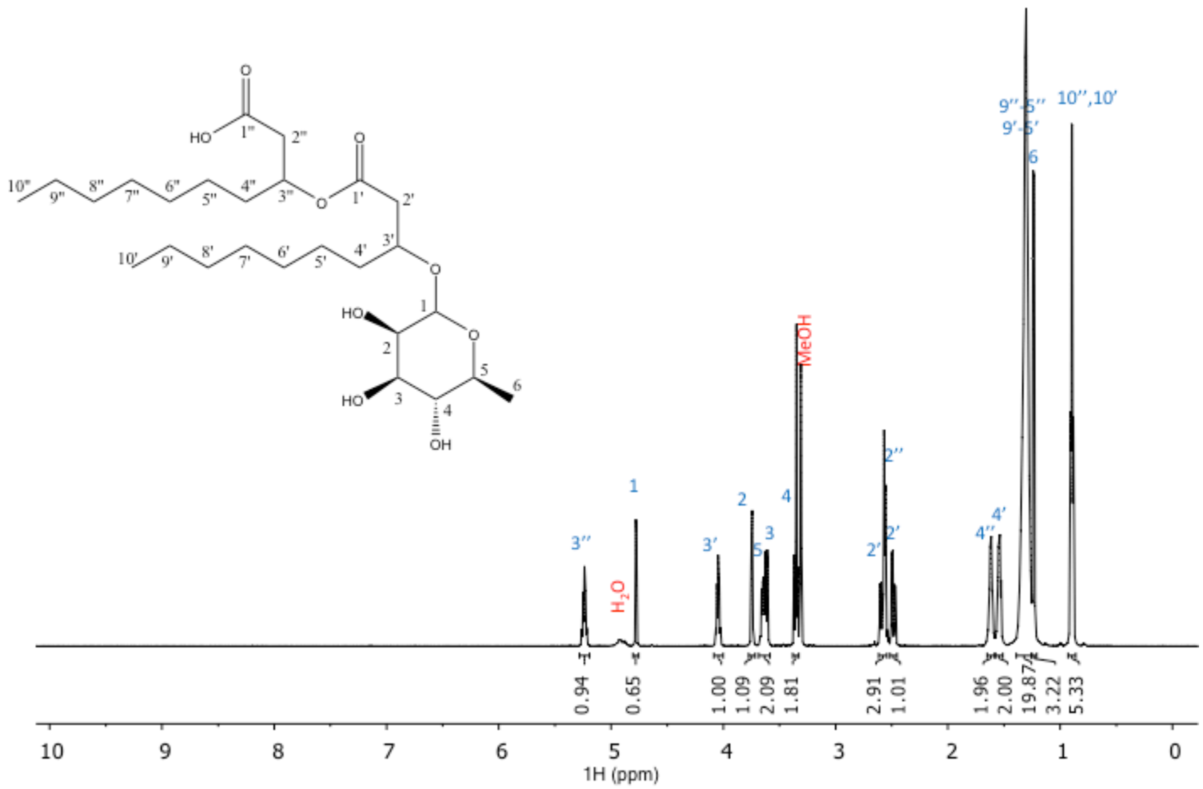

Figure S9. 1D proton of **2** in methanol-d4, T=298 K.

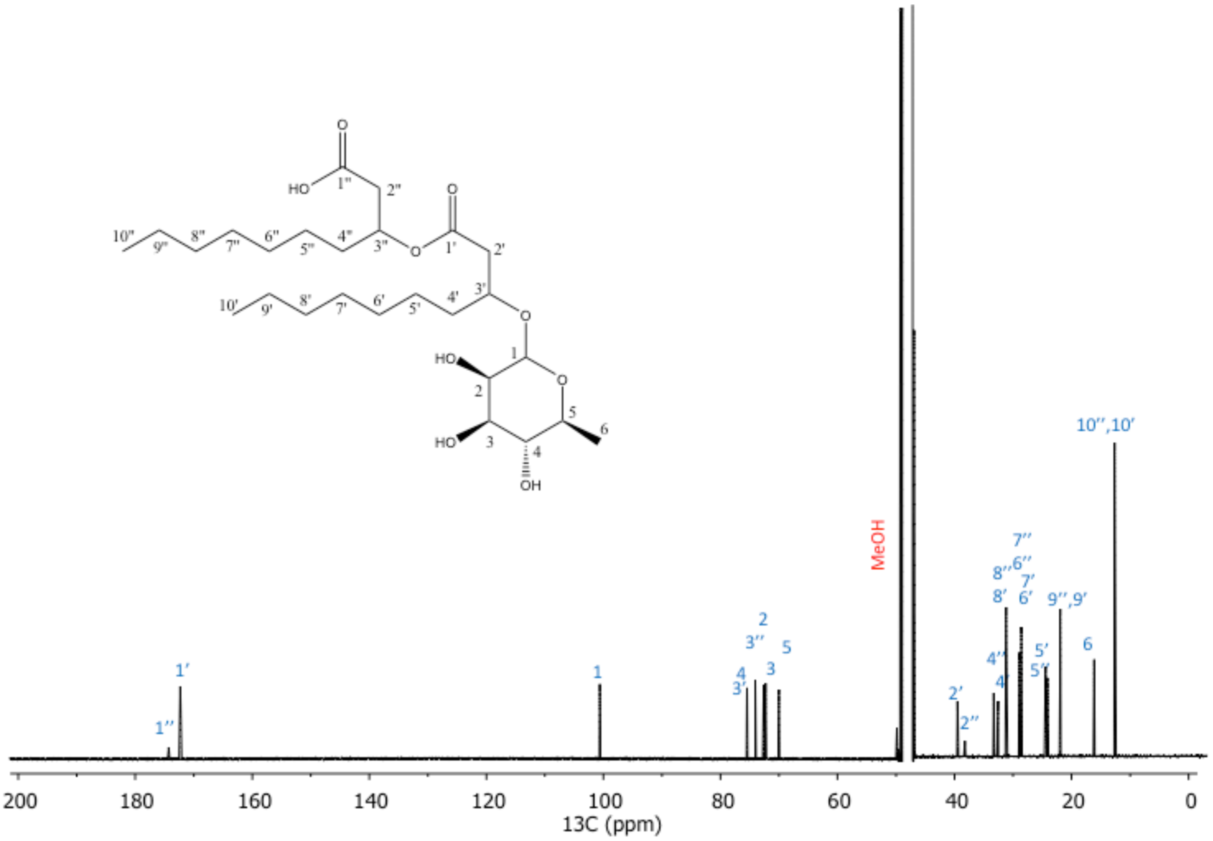

Figure S10. 1D carbon of **2** in methanol-d4, T=298 K.

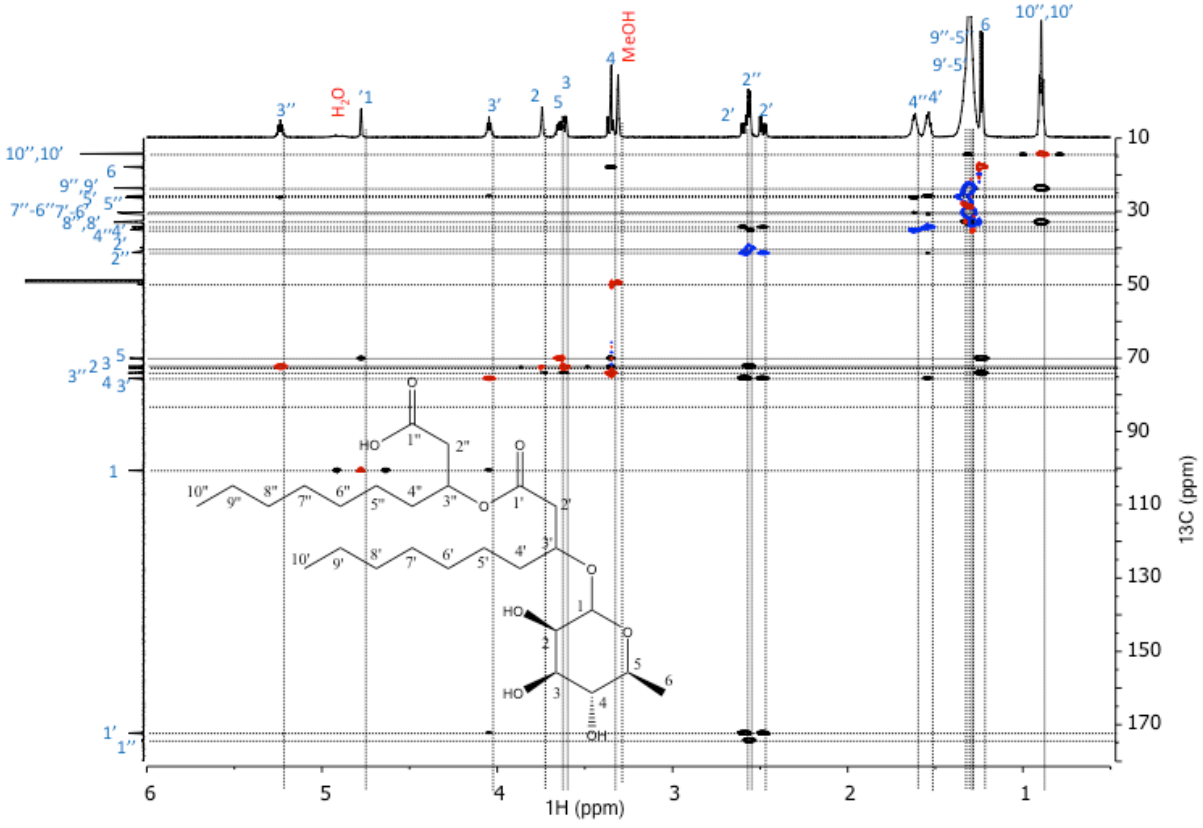

Figure S11. 2D superimposed ^{13}C-HSQC and HMBC of **2** in methanol-d4, T=298 K.

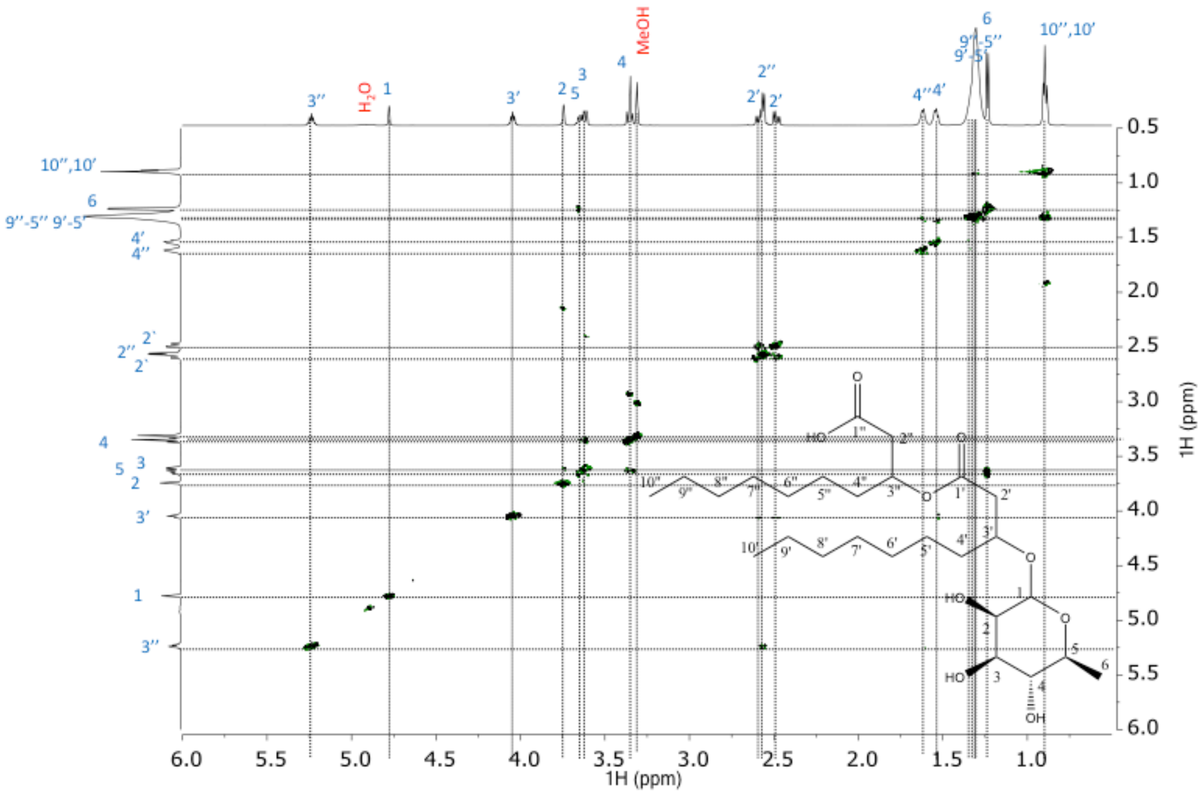

Figure S12. 2D DQF-COSY of **2** in methanol-d4, T=298 K.

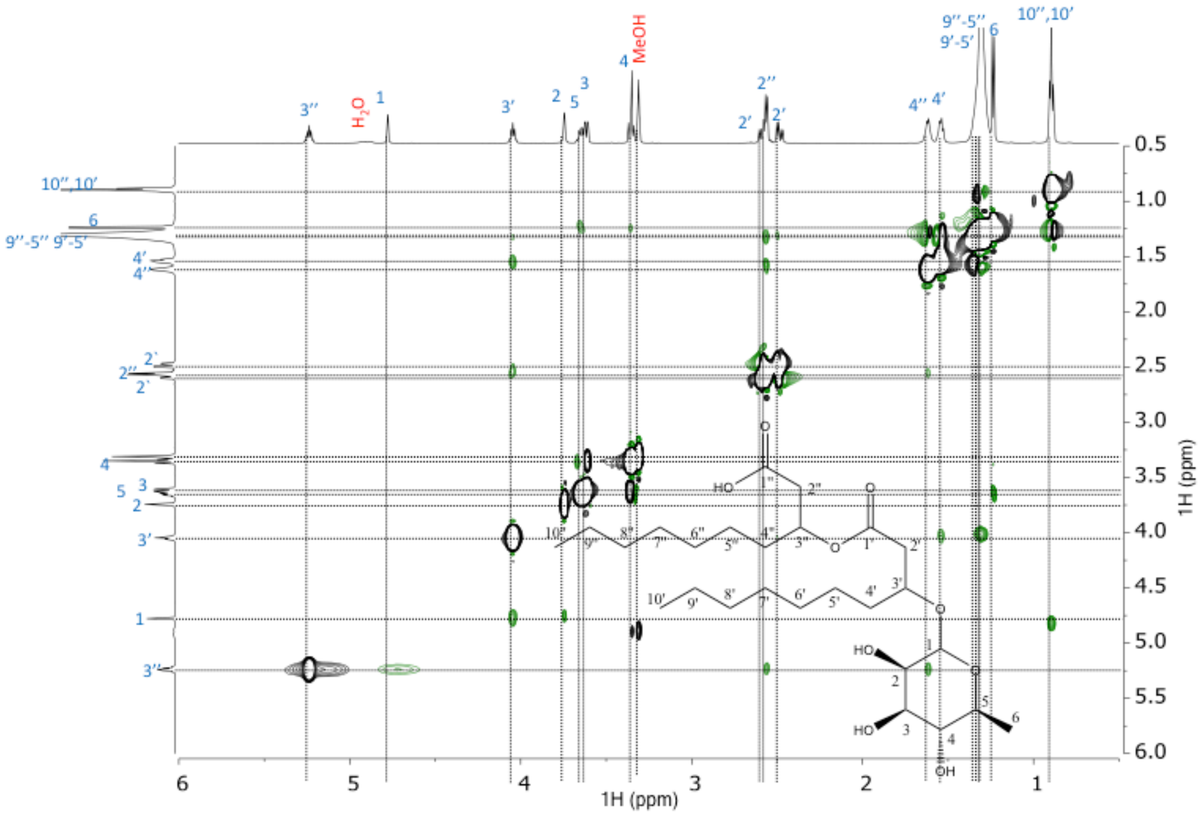

Figure S13. 2D ROESY (300 ms) of **2** in methanol-d4, T=298 K.

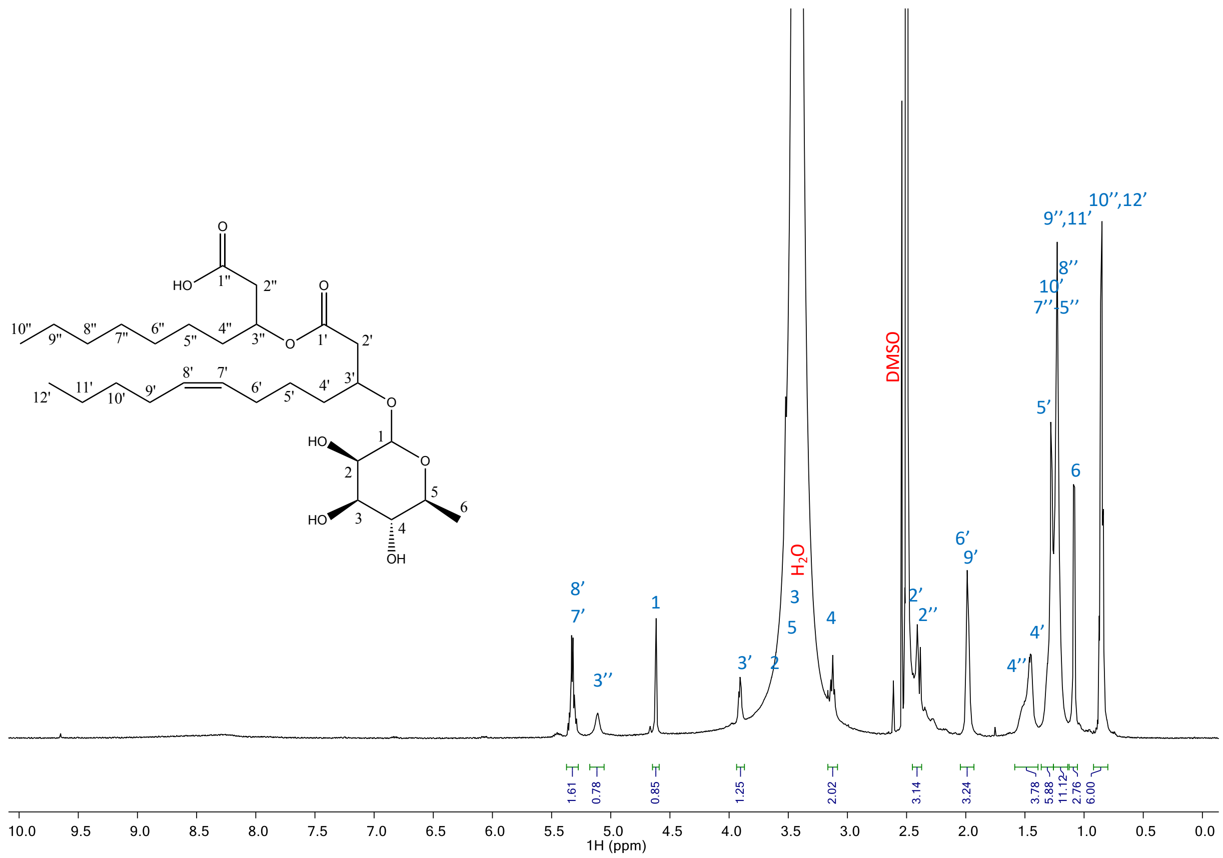

Figure S14. 1D proton of **3** in DMSO-d6, T=298 K.

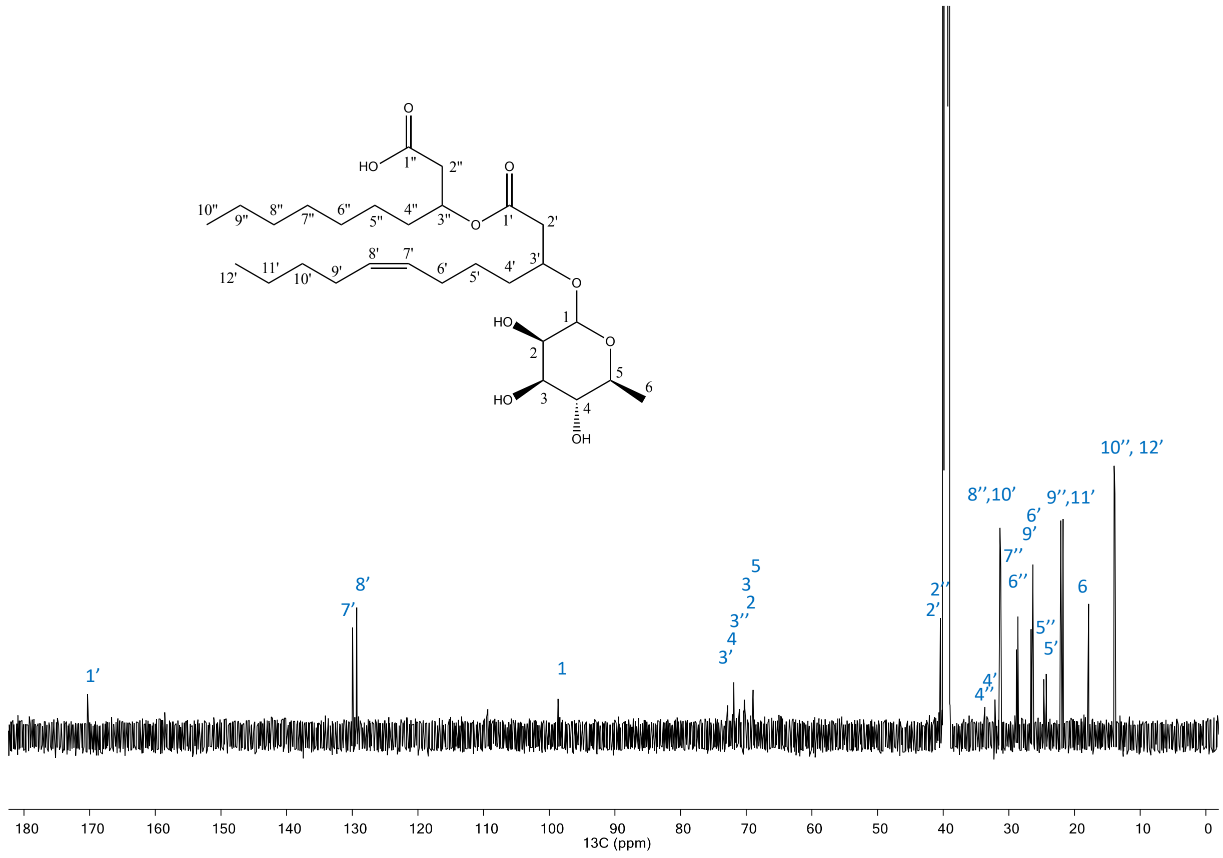

Figure S15. 1D carbon of **3** in DMSO-d6, T=298 K.

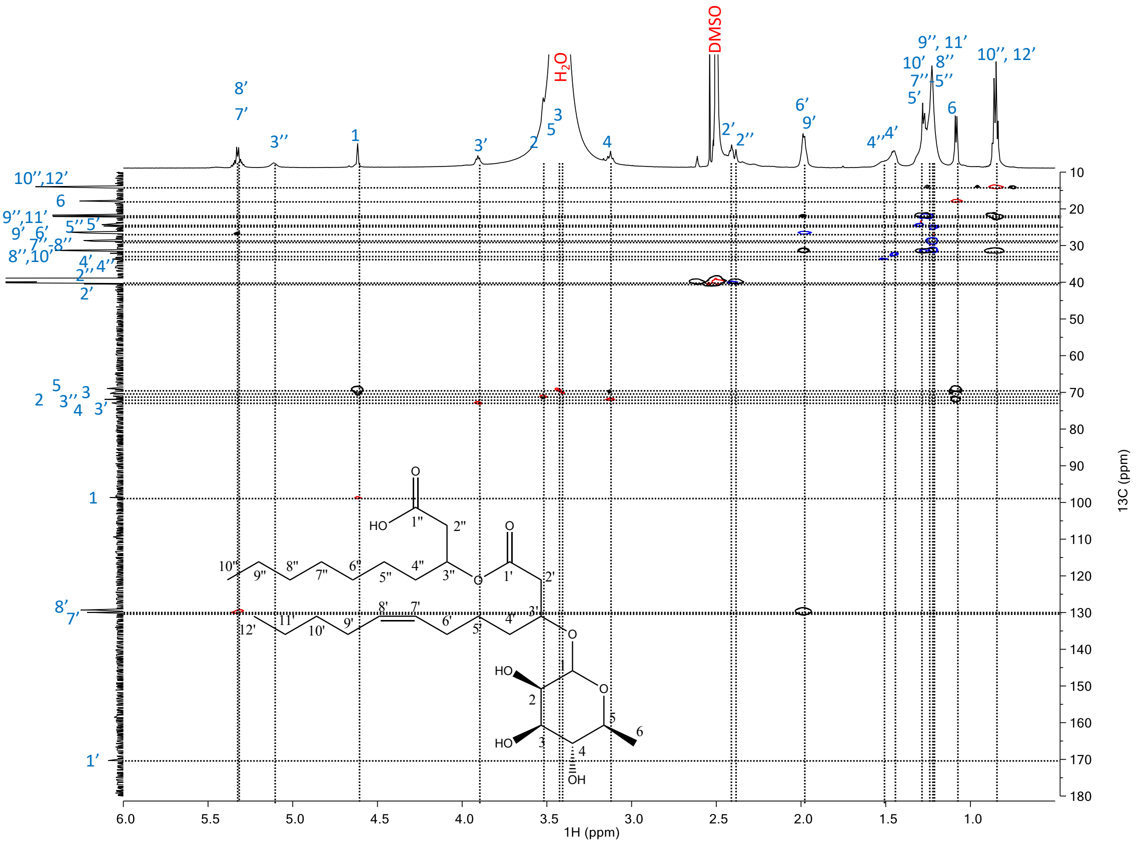

Figure S16. 2D superimposed ^{13}C-HSQC and HMBC of **3** in DMSO-d$_6$, T=298 K.

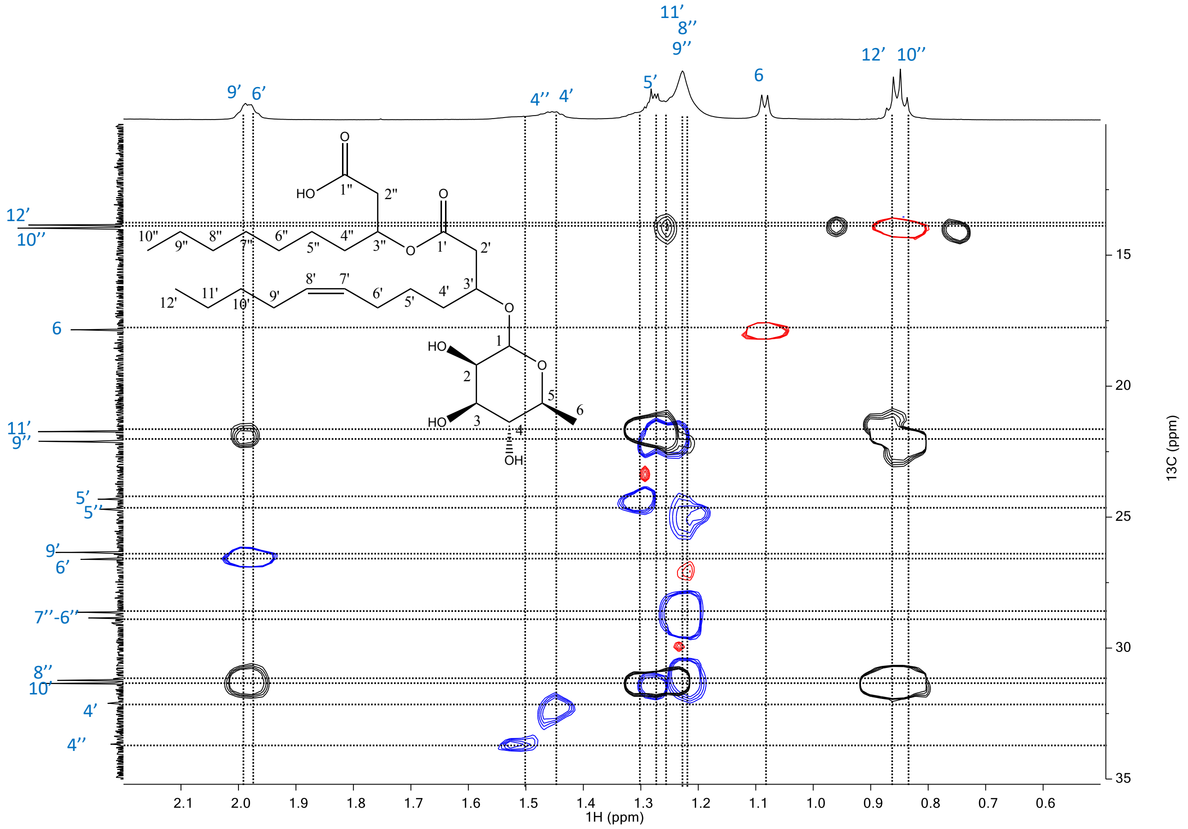

Figure S17. Blown up aliphatic region of superimposed 2D ^{13}C-HSQC and HMBC of **3** in DMSO-d$_6$, T=298 K.

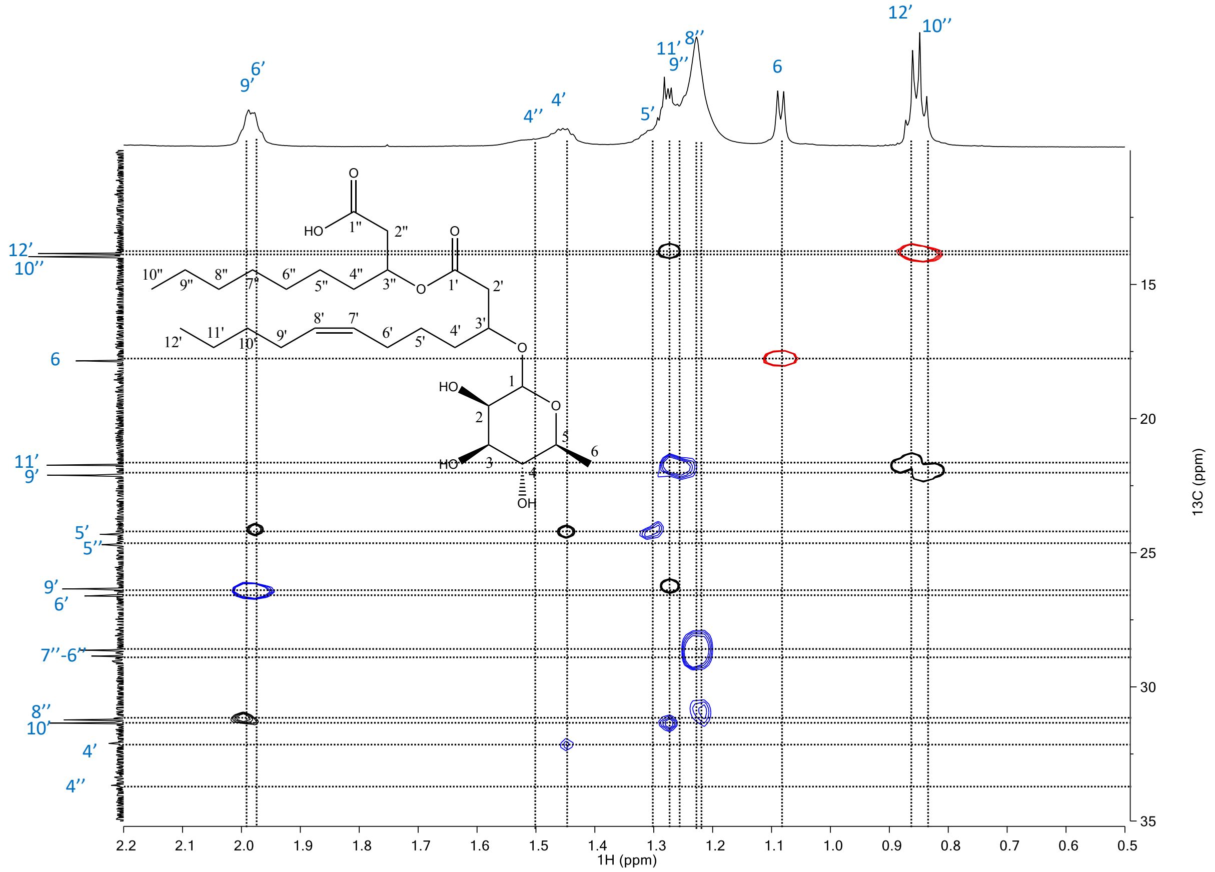

Figure S18. Blown up aliphatic region of superimposed 2D ^{13}C-HSQC and H2BC of **3** in DMSO-d$_6$, T=298 K.

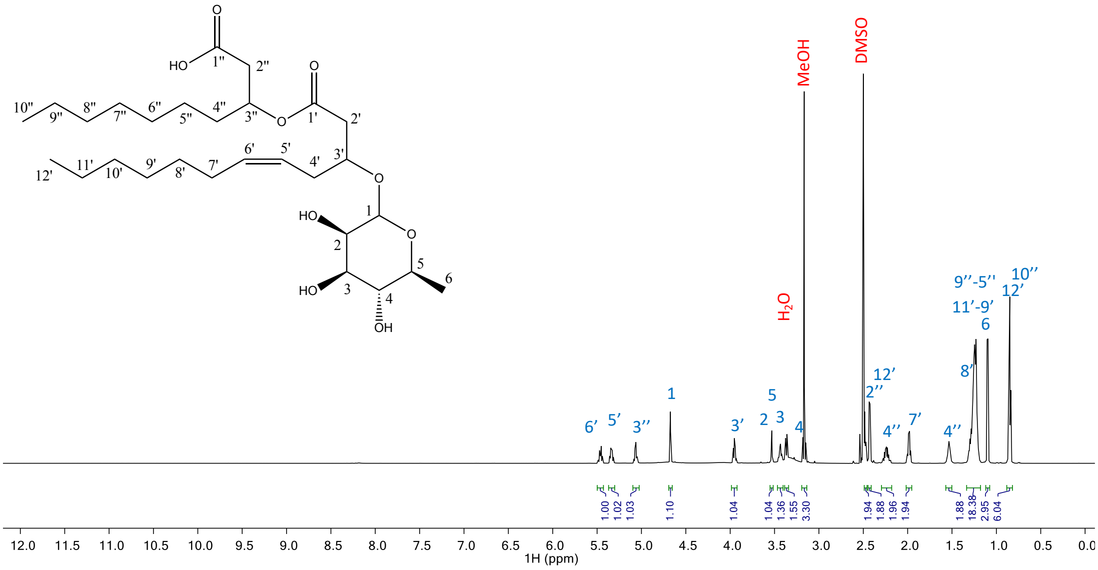

Figure S19. 1D proton of **4** in DMSO-d$_6$, T=298 K.

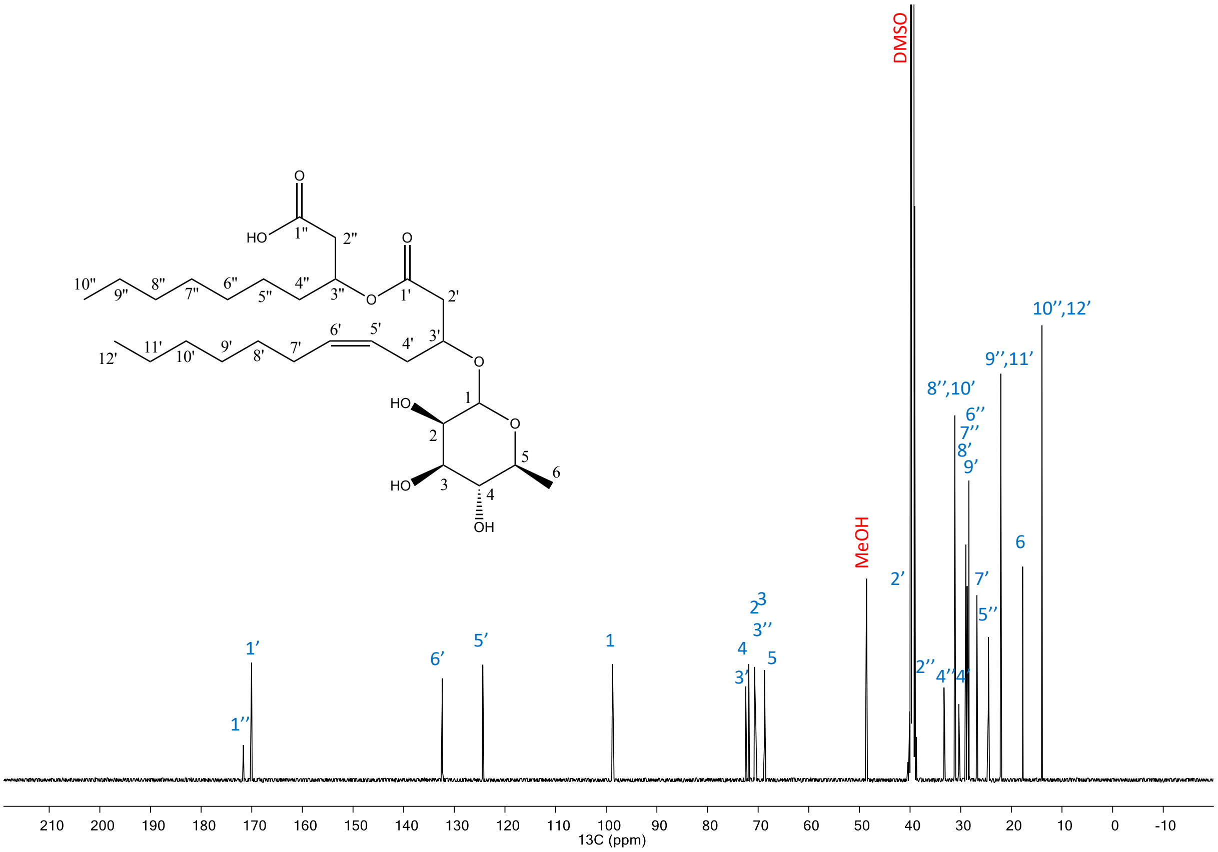

Figure S20. 1D carbon of **4** in DMSO-d6, T=298 K.

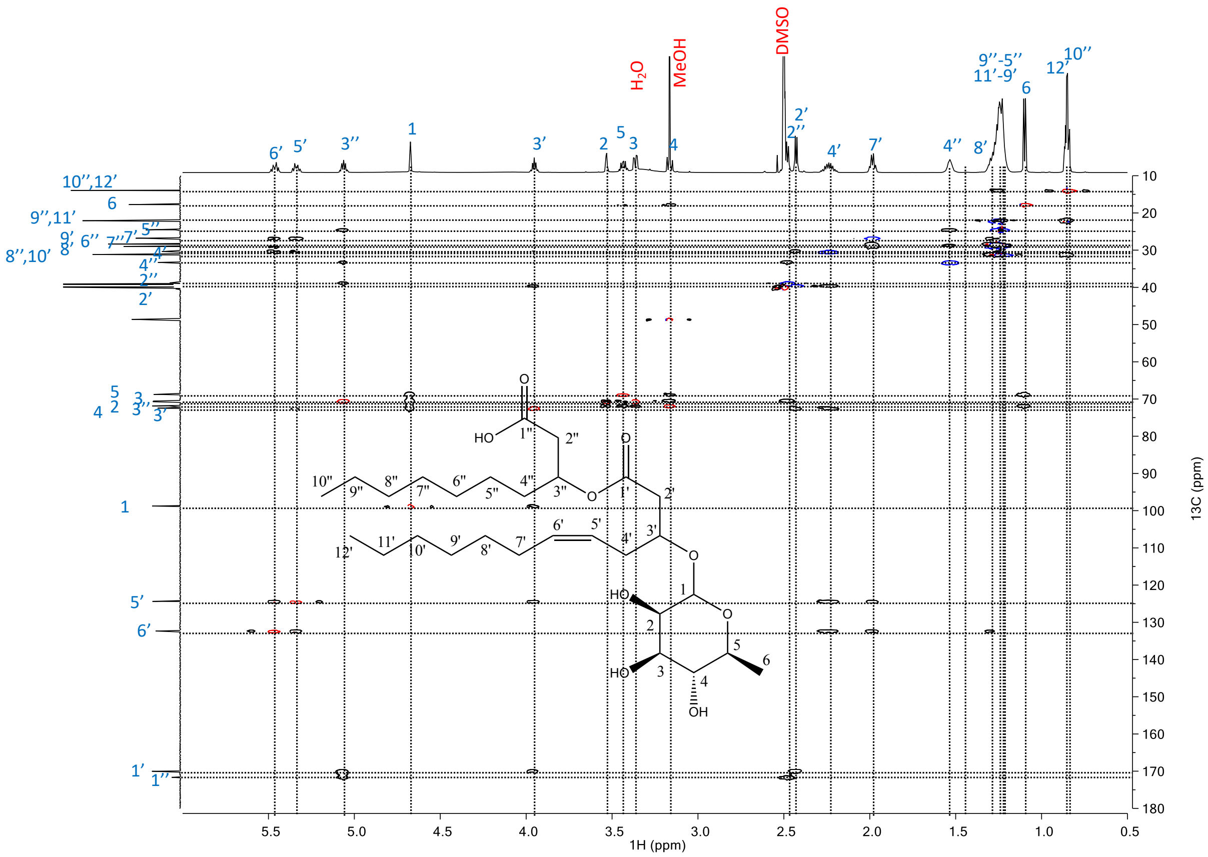

Figure S21. 2D superimposed ^{13}C-HSQC and HMBC of **4** in DMSO-d6, T=298 K.

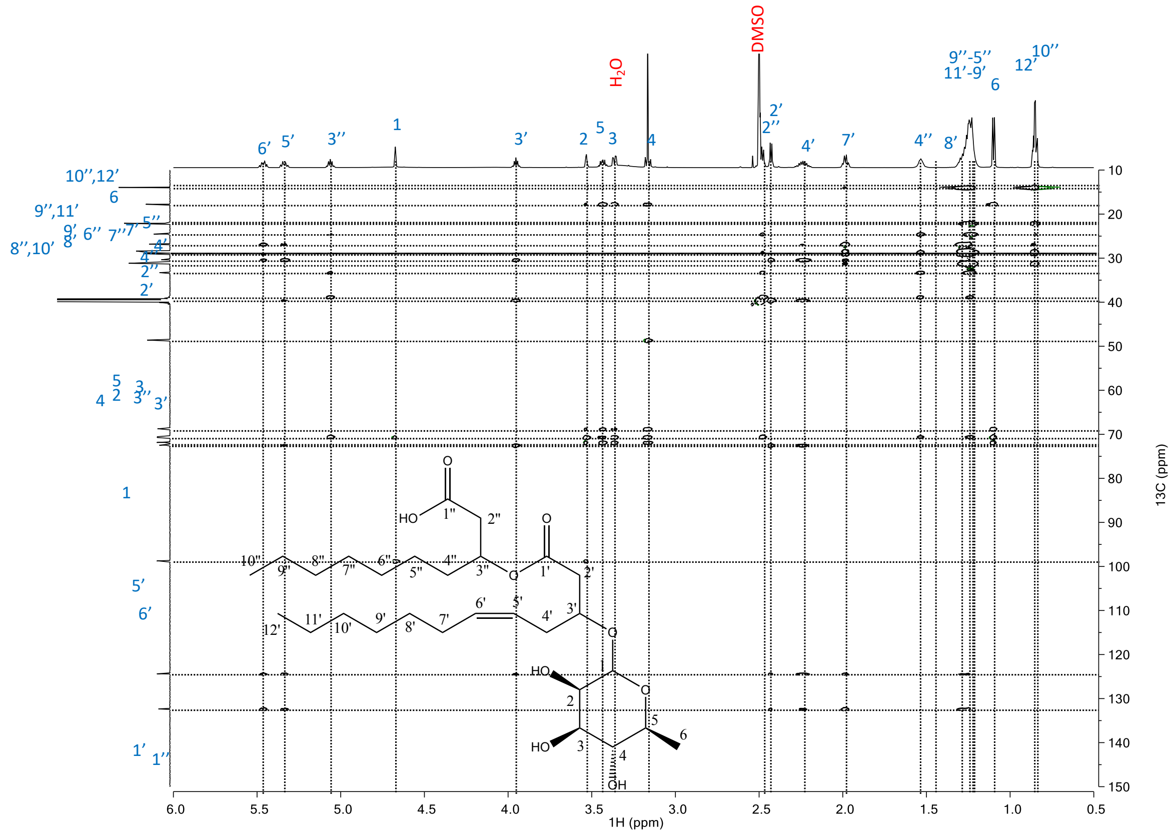

Figure S22. 2D ^{13}C-HSQCTOCSY of **4** in DMSO-d$_6$, T=298 K.

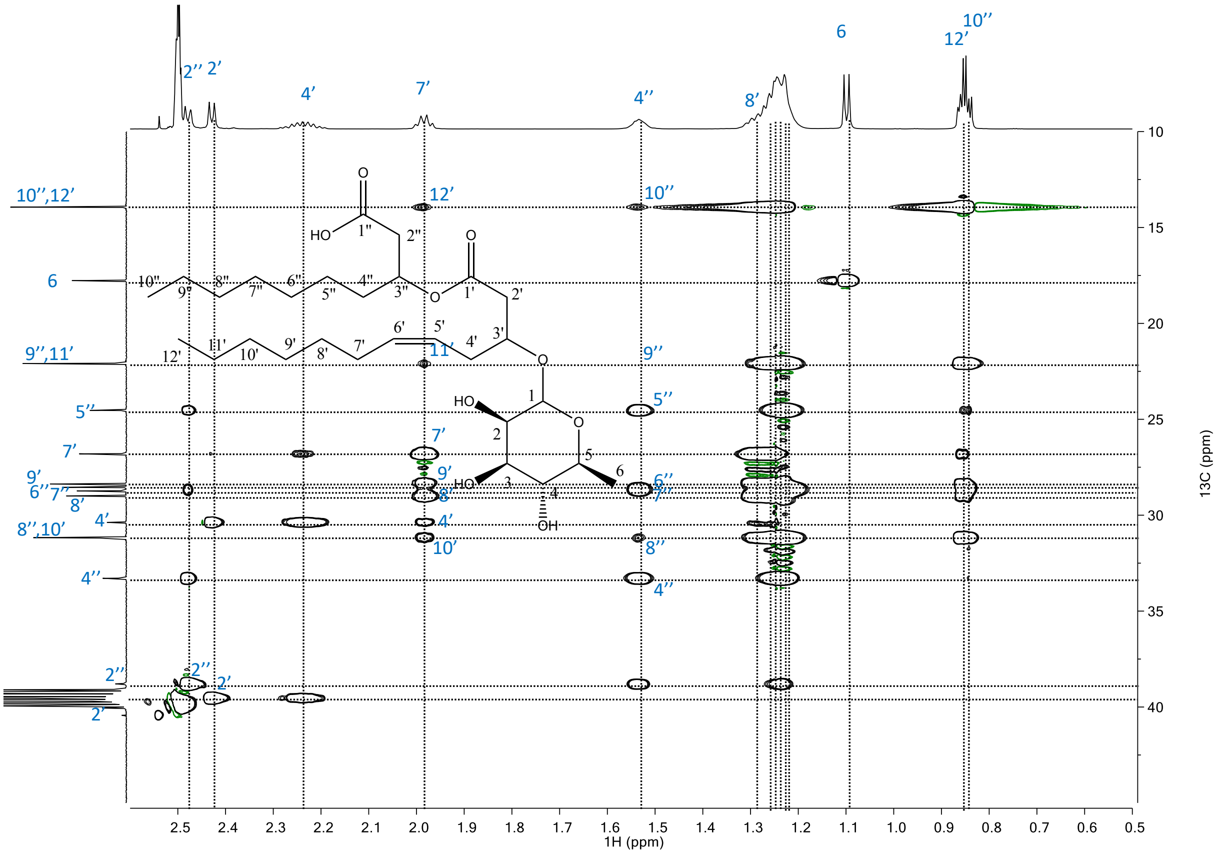

Figure S23. Blown up aliphatic region of the 2D ^{13}C-HSQCTOCSY of **4** in DMSO-d$_6$, T=298 K.

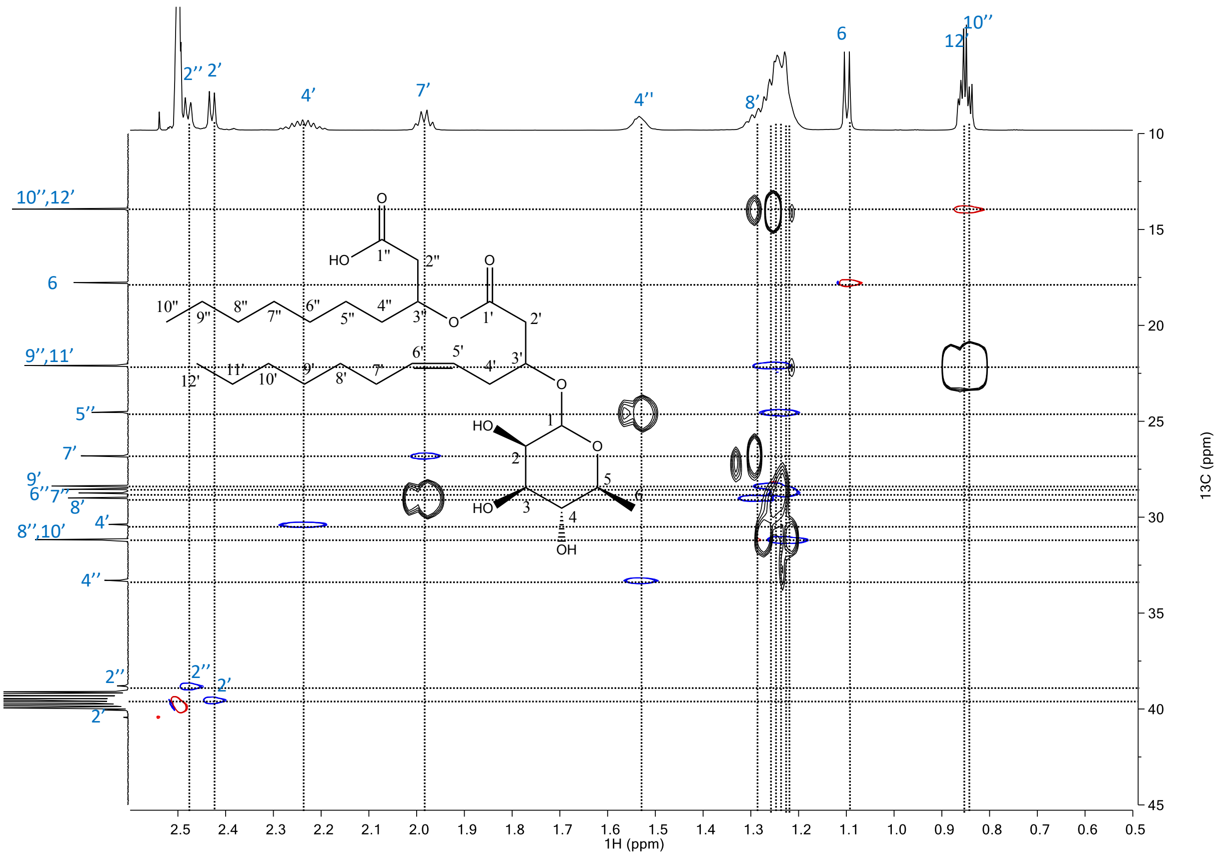

Figure S24. Blown up aliphatic region of superimposed 2D ^{13}C-HSQC and H2BC of **4** in DMSO-d$_6$, T=298 K.

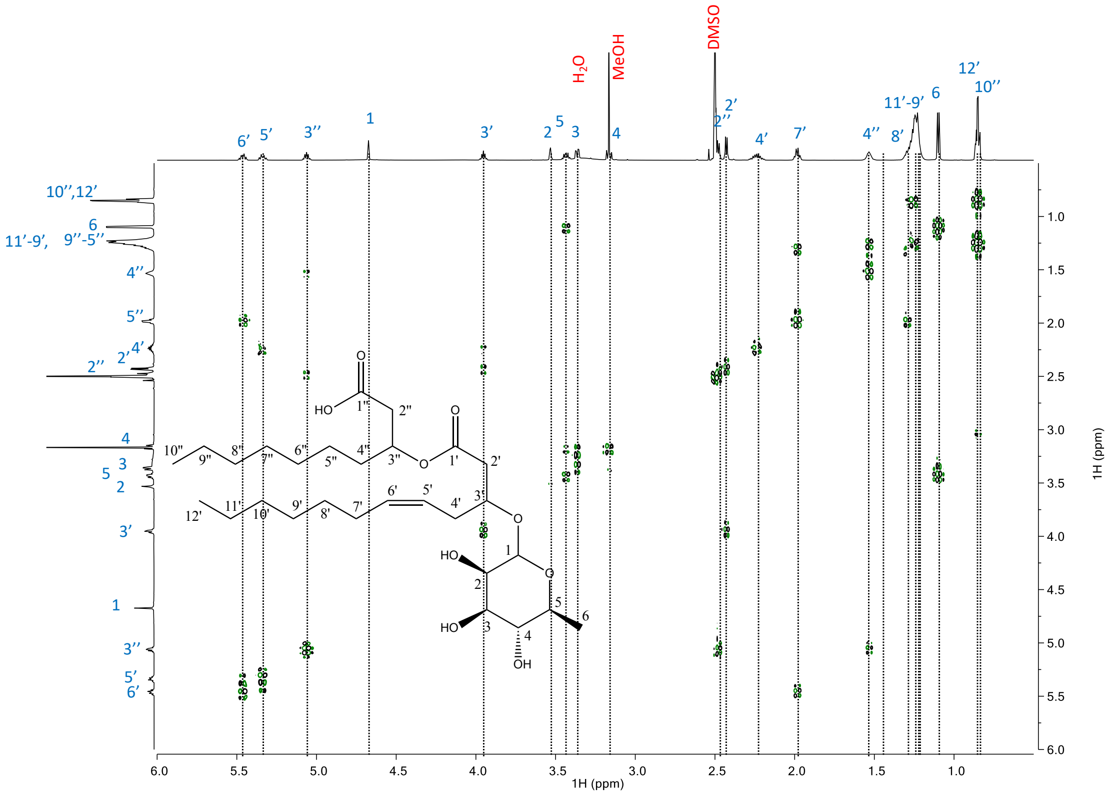

Figure S25. 2D DQF-COSY of **4** in DMSO-d$_6$, T=298 K.

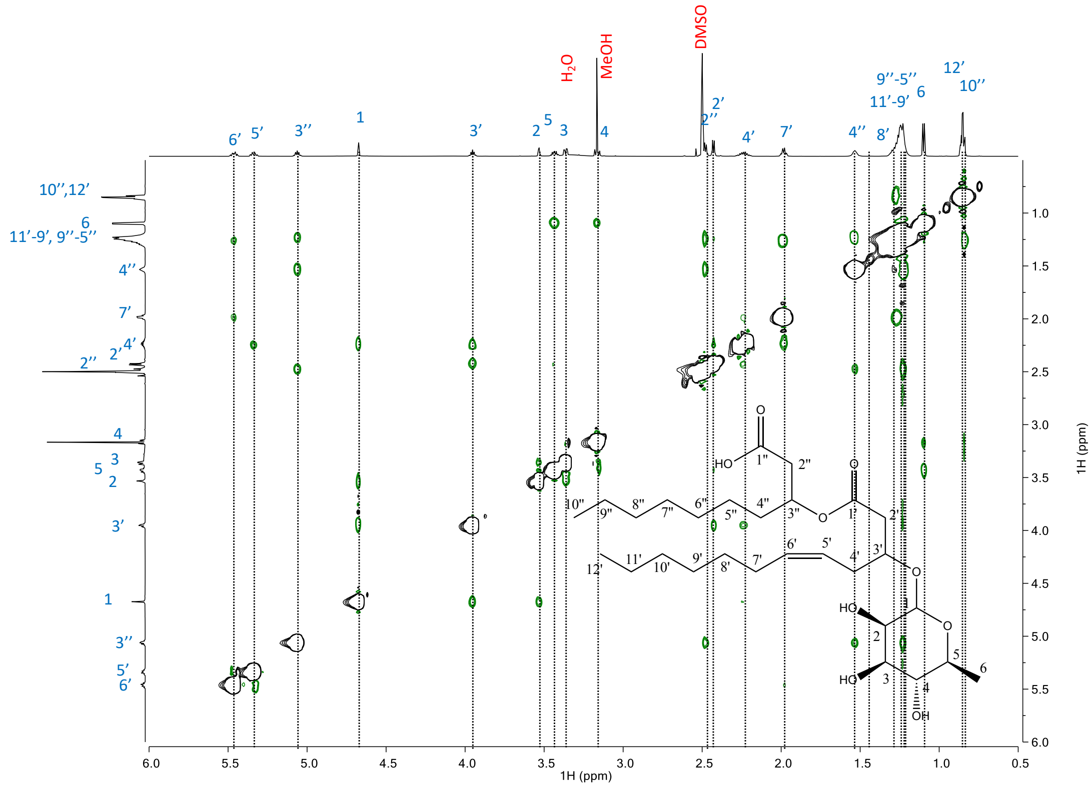

Figure S26. 2D ROESY (300 ms) of **4** in DMSO-d₆, T=298 K.

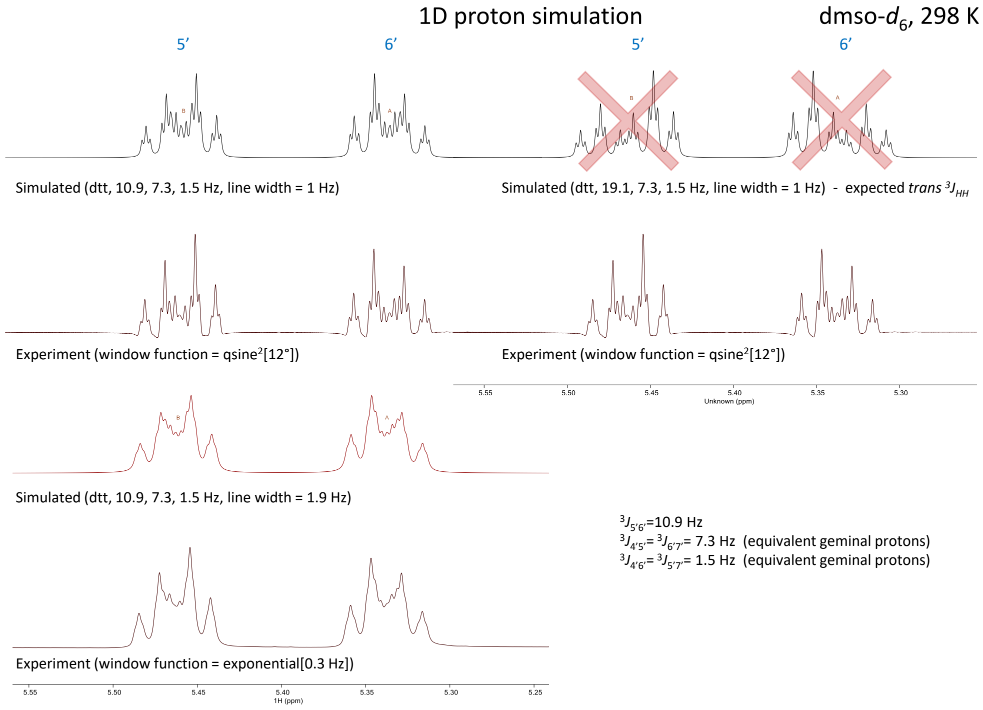

Figure S27. Comparison between experimental and simulated multiplets of the olefinic protons of **4**. The fit was made using a qsine²(12°) window function (upper panel), and then compared to the raw multiplets (lower panel). The best fit was found to be a dtt, 10.9, 7.3, 1.5 Hz multiplet, which indicates a *cis* configuration (expected $^3J_{5'6'}$ ~ 11 Hz). The right panel shows the simulated peak for the expected *trans* coupling ~19 Hz with shows a poor fit.

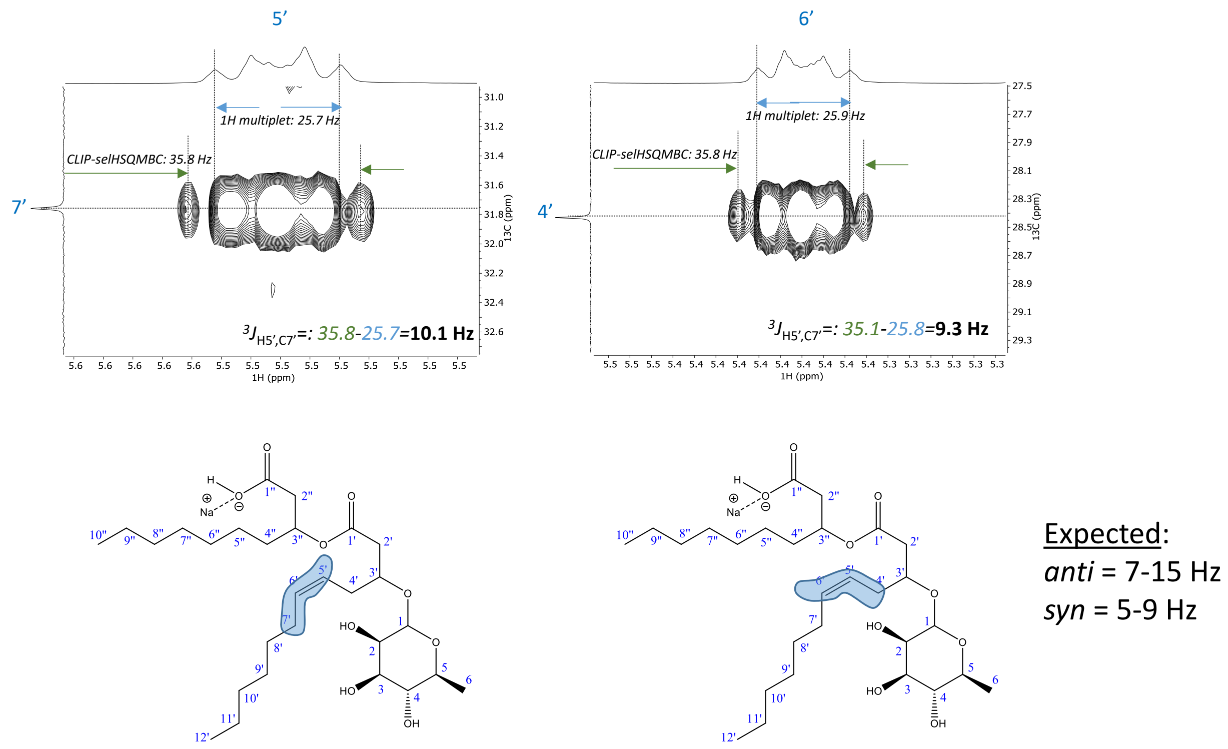

Figure S28. The $^3J_{CH}$ couplings of the olefinic protons are estimated using a selective CLIP-HSQMBC experiment. Values of 9.3 and 10.1 Hz is found for $^3J_{H6'C4'}$ and $^3J_{H5'C7'}$ respectively, suggesting anti configurations of the C and H.

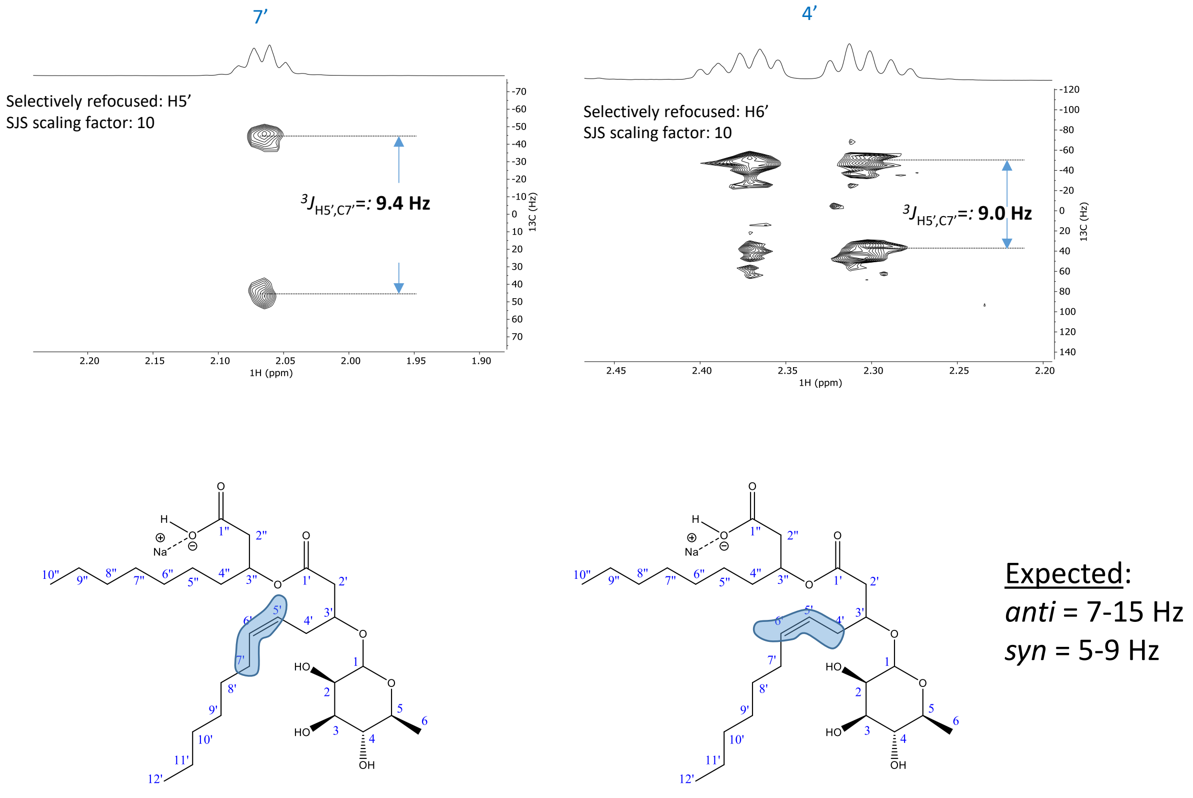

Figure S29. *The* $^3J_{CH}$ couplings of the olefinic protons are estimated using a SJS-HSQC experiment (j-resolved in f1). Values of 9.0 and 9.4 Hz is found for $^3J_{H6'C4'}$ and $^3J_{H5'C7'}$ respectively, suggesting anti configurations of the C and H.

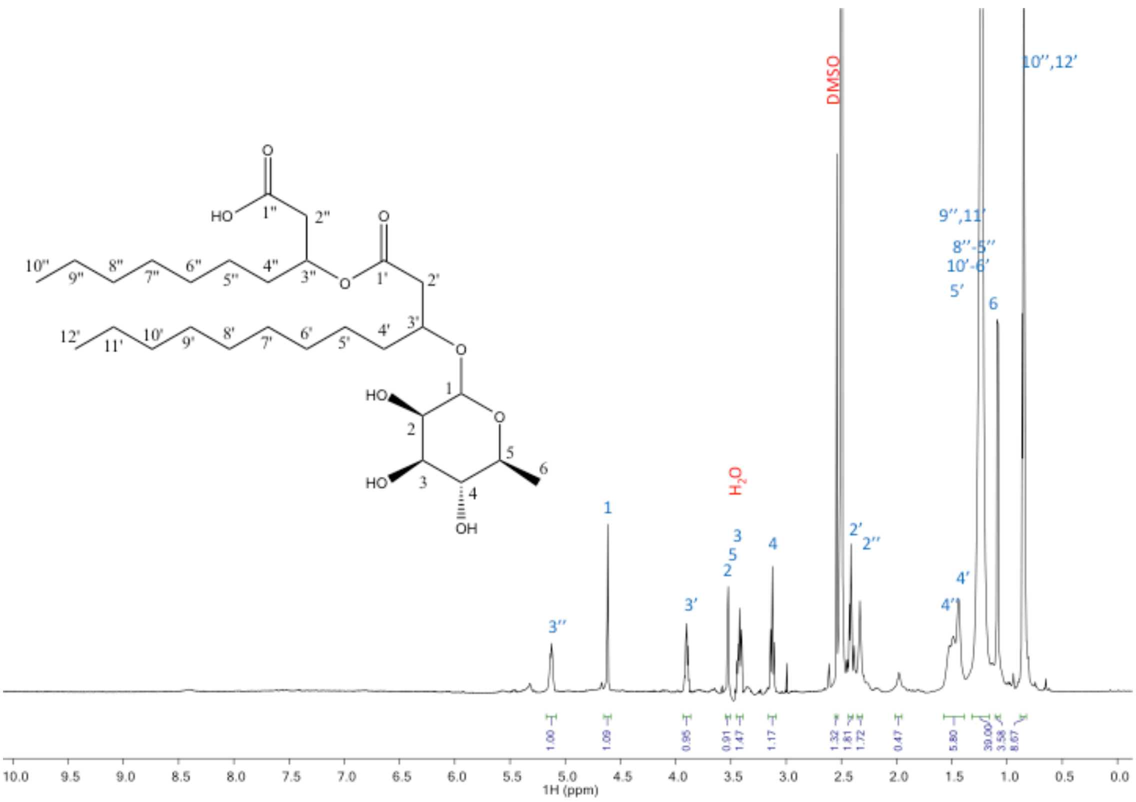

Figure S30. 1D proton of **5** in DMSO-d6, T=298 K.

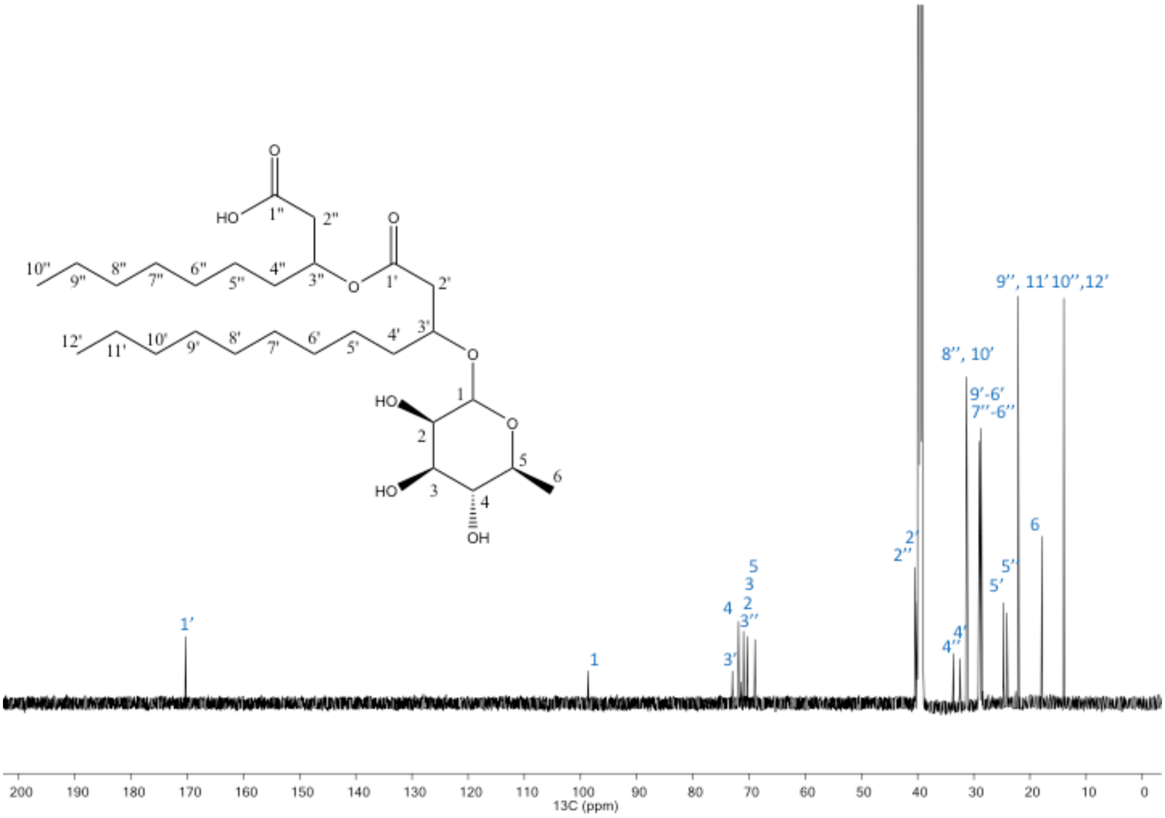

Figure S31. 1D carbon of **5** in DMSO-d6, T=298 K.

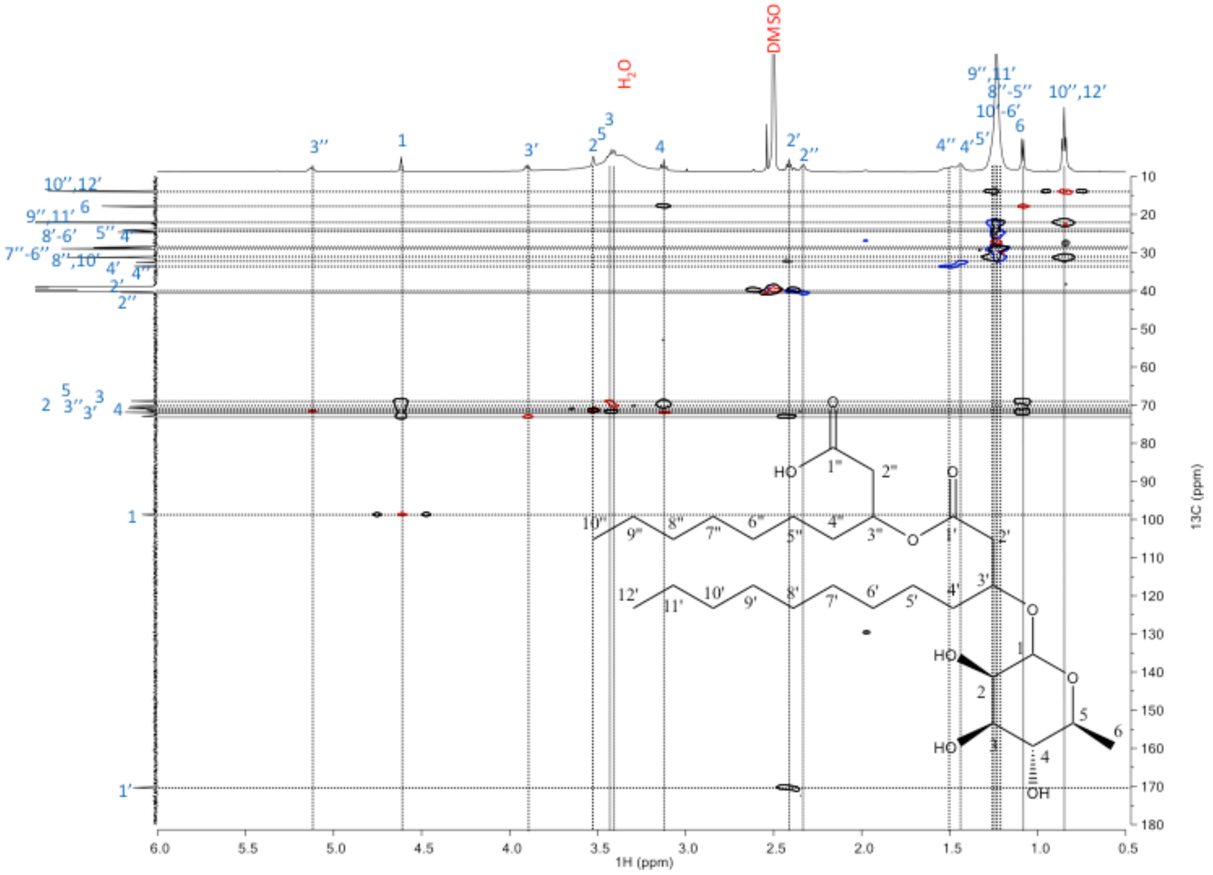

Figure S32. 2D superimposed ^{13}C-HSQC and HMBC of **5** in DMSO-d6, T=298 K.

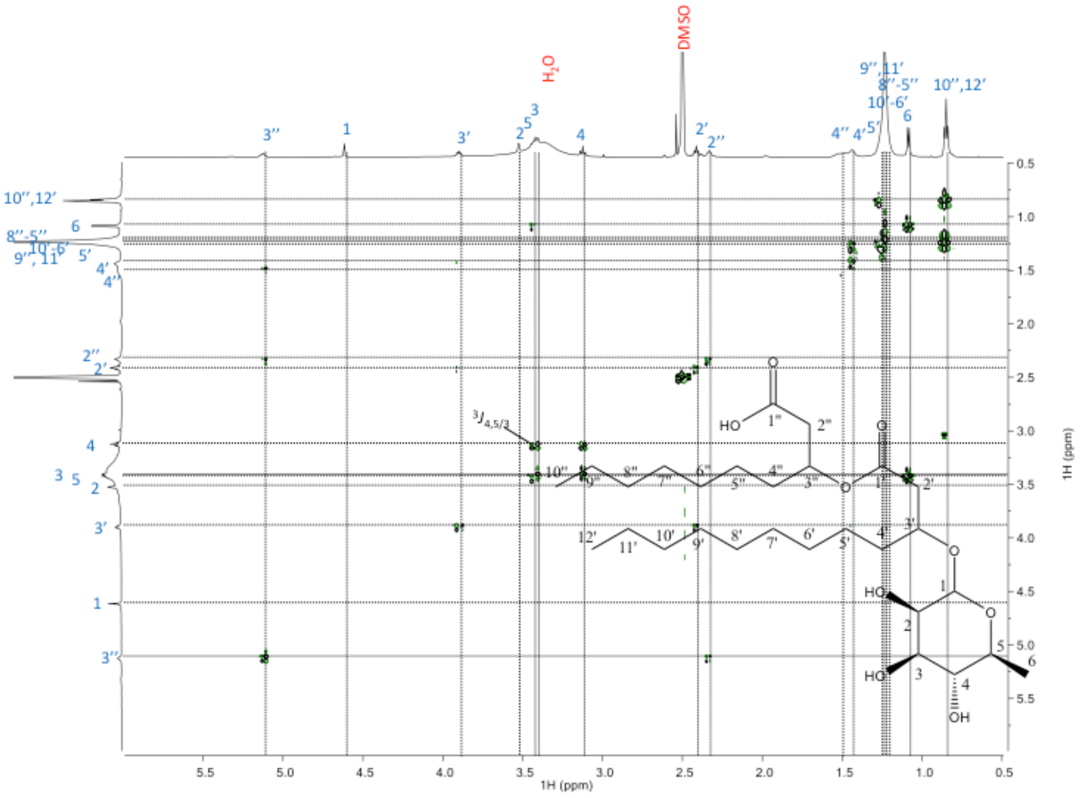

Figure S33. 2D DQF-COSY of **5** in DMSO-d6, T=298 K.

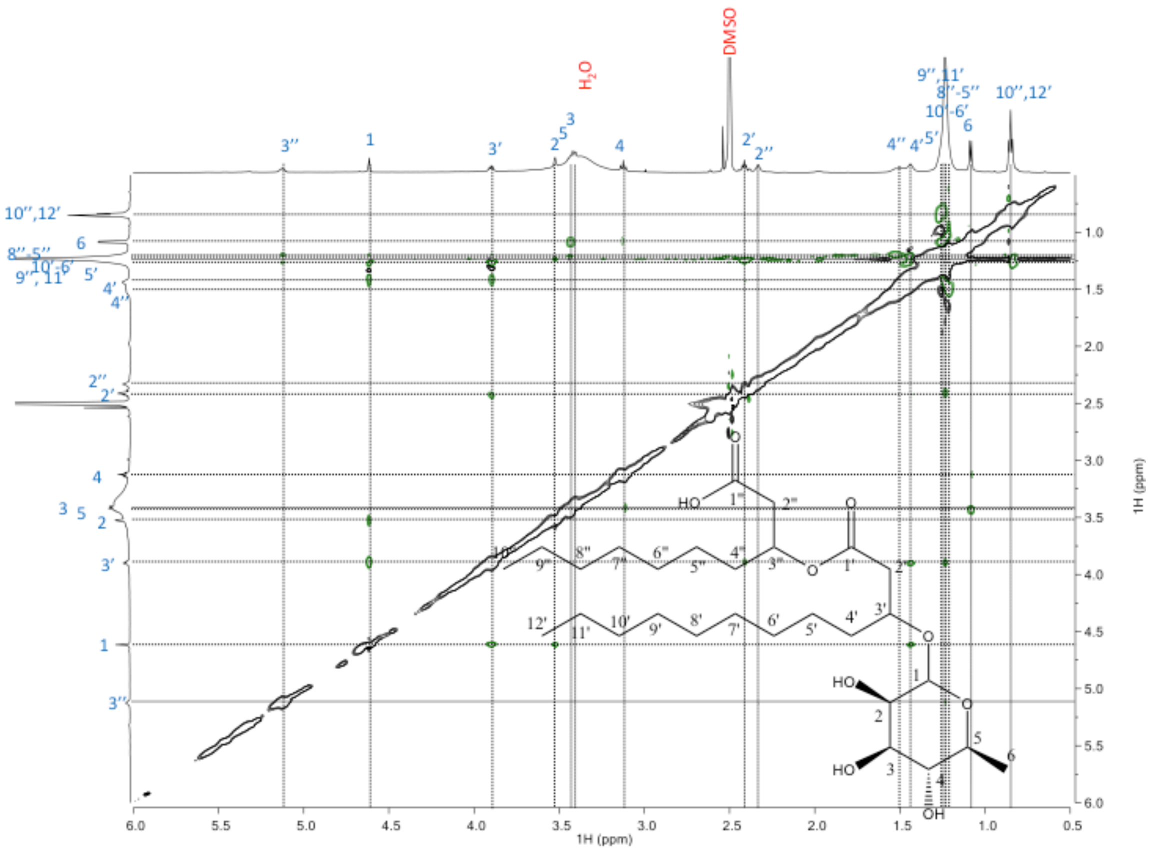

Figure S34. 2D ROESY (300 ms) of **5** in DMSO-d6, T=298 K.

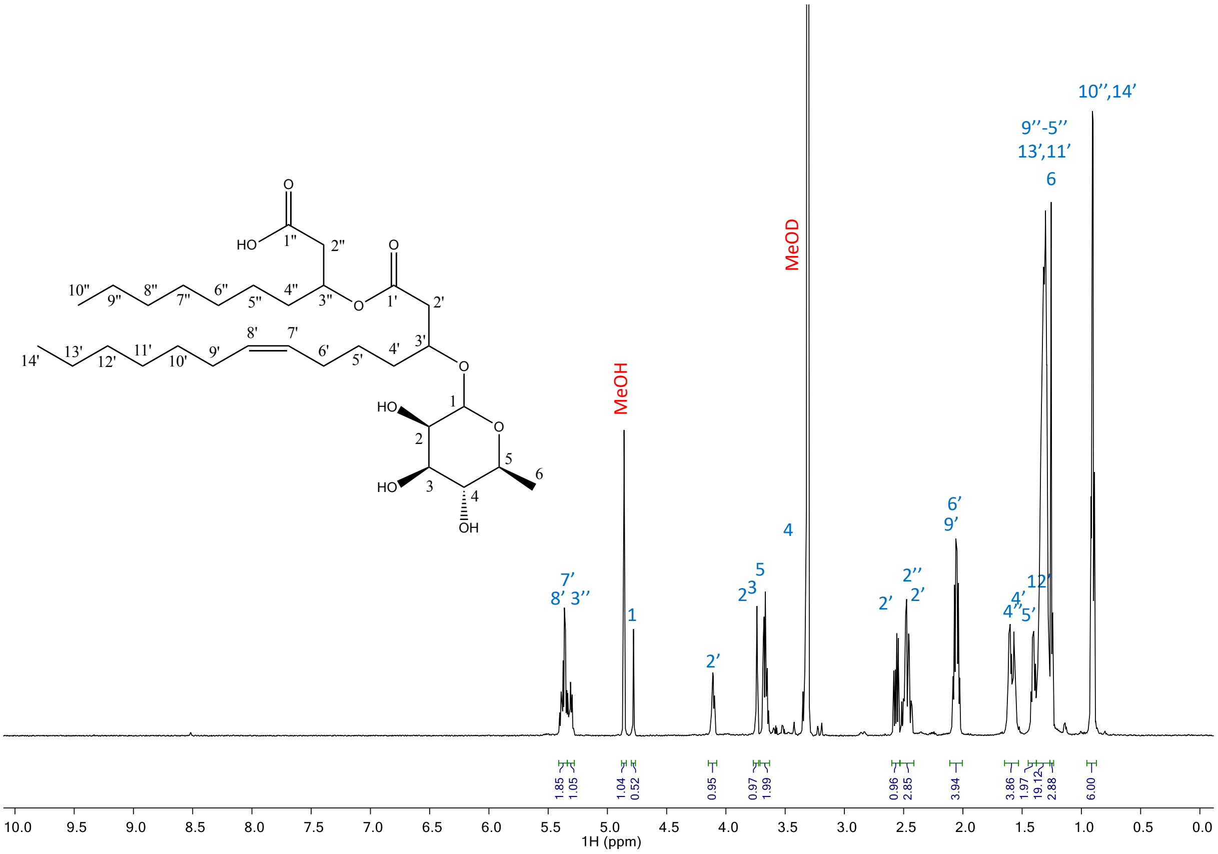

Figure S35. 1D proton of **6** in methanol-d4, T=298 K.

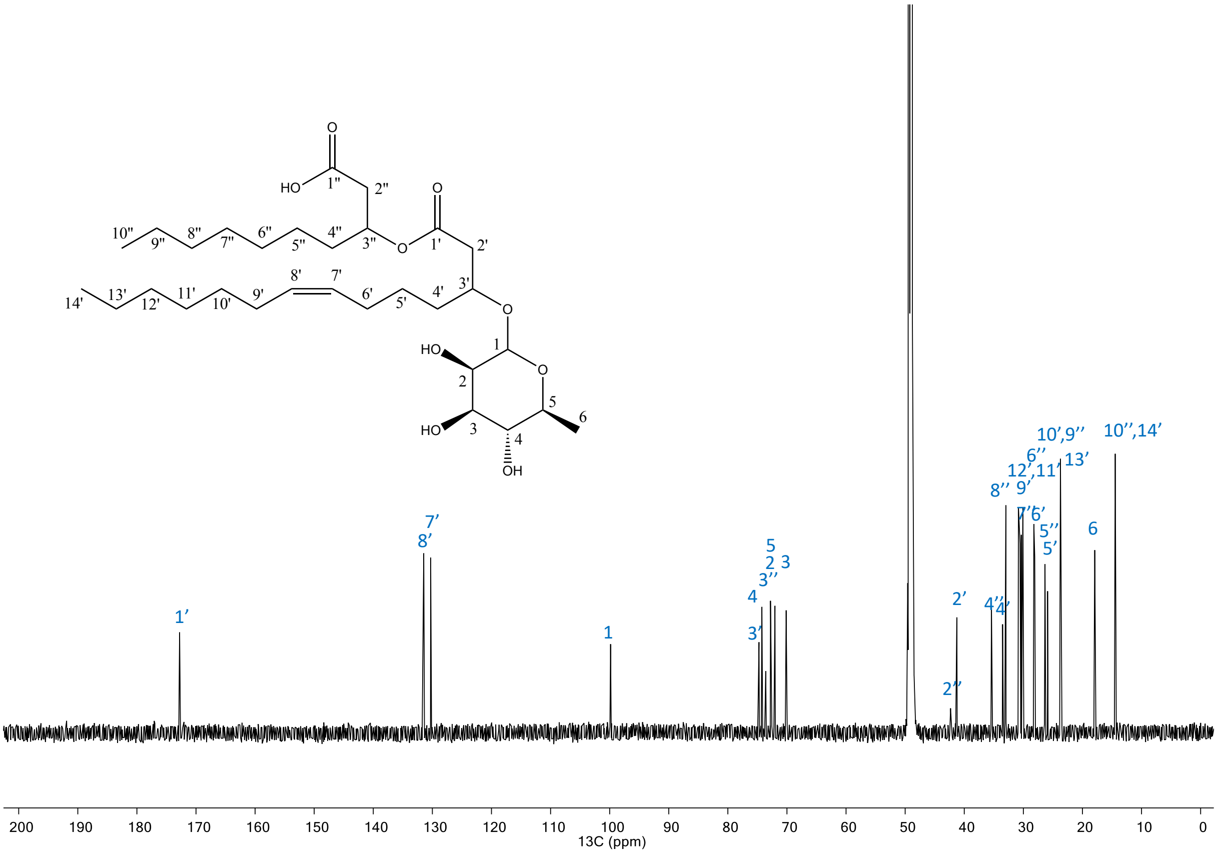

Figure S36. 1D carbon of **6** in methanol-d$_4$, T=298 K.

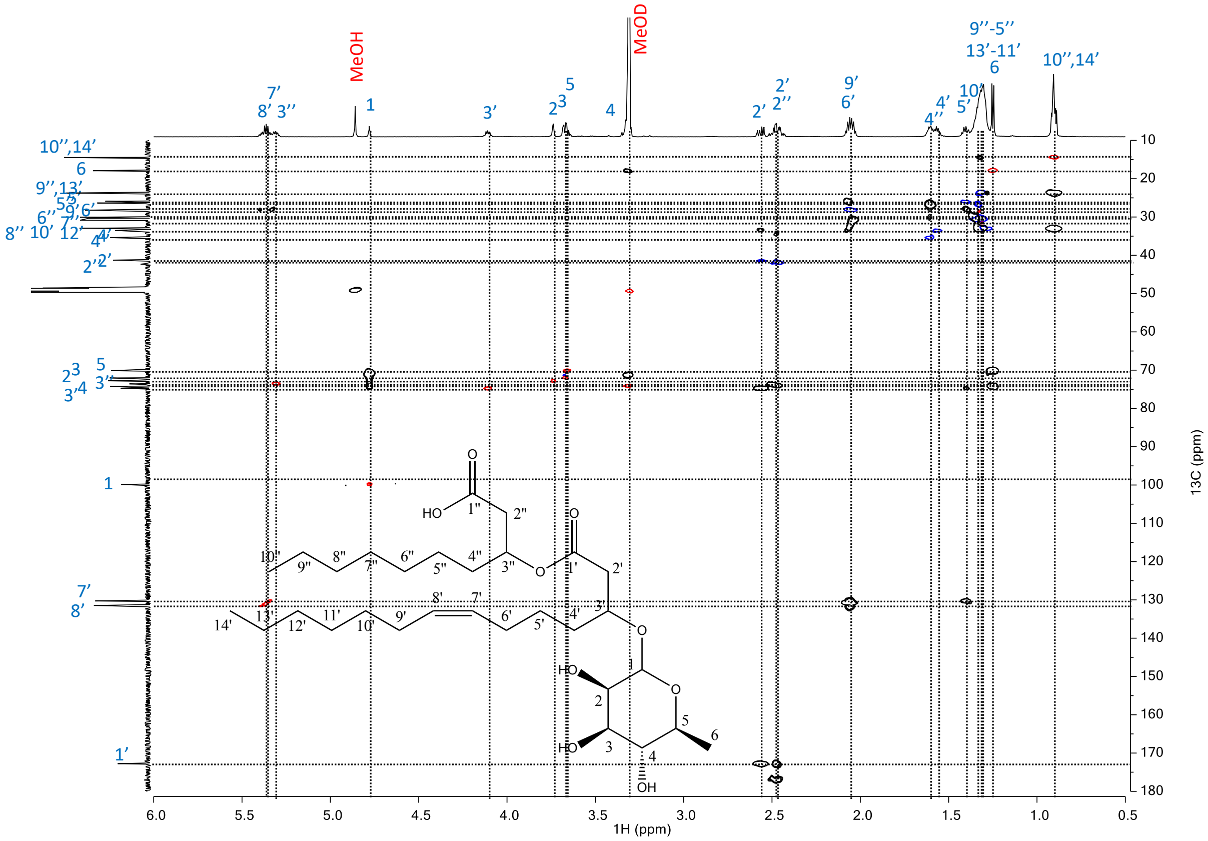

Figure S37. 2D superimposed ^{13}C-HSQC and HMBC of **6** in methanol-d$_4$, T=298 K.

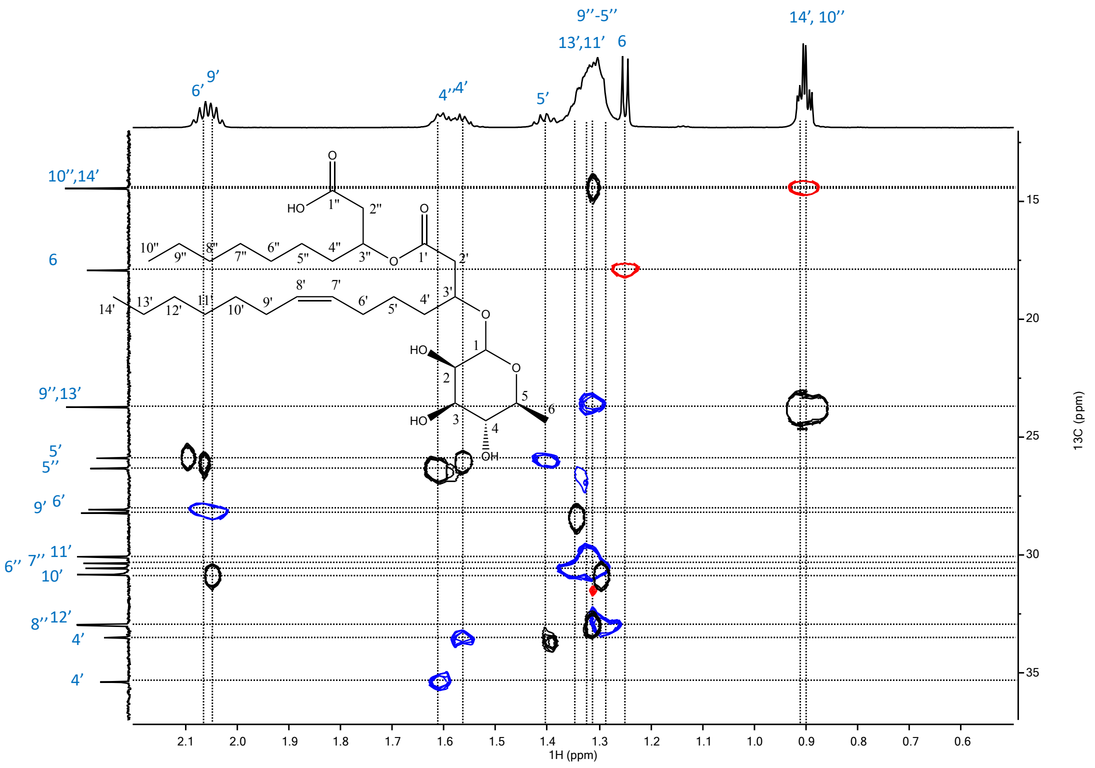

Figure S38. Blown up aliphatic region of superimposed 2D ^{13}C-HSQC and H2BC of **6** in methanol-d$_4$, T=298 K.

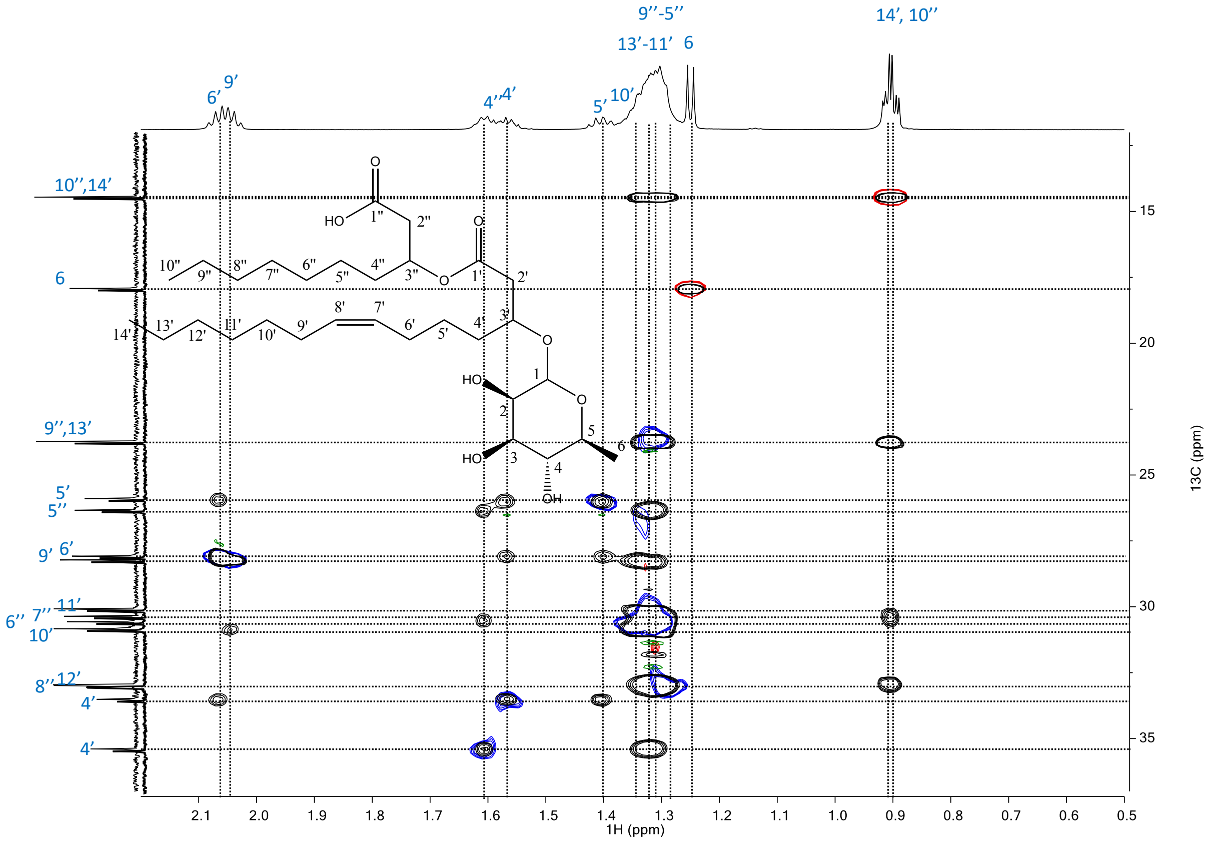

Figure S39. Blown up aliphatic region of superimposed 2D ^{13}C-HSQC and HSQC-TOCSY of **6** in methanol-d$_4$, T=298 K.

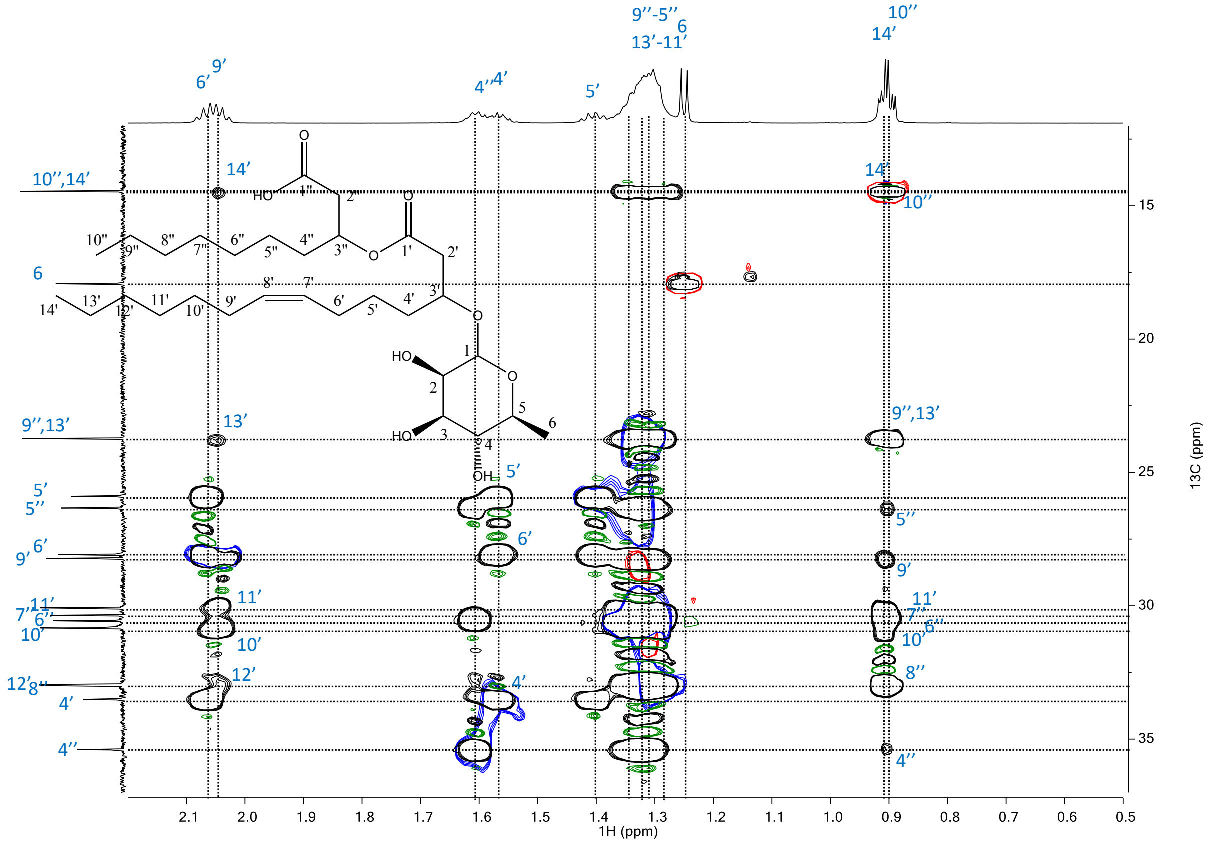

Figure S40. 2D ^{13}C-HSQC and HSQC-TOCSY at noise level of **6** in methanol-d$_4$, T=298 K.

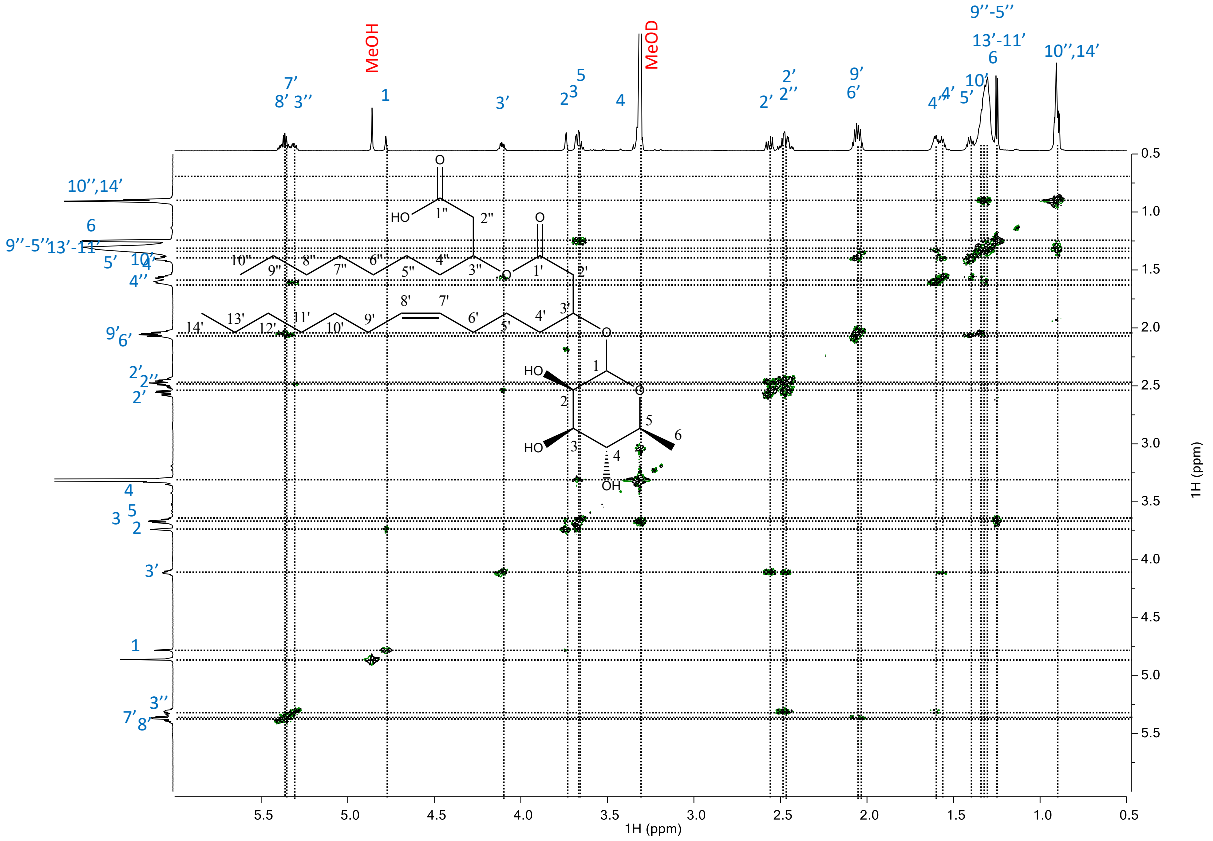

Figure S41. 2D DQF-COSY of **6** in methanol-d$_4$, T=298 K.

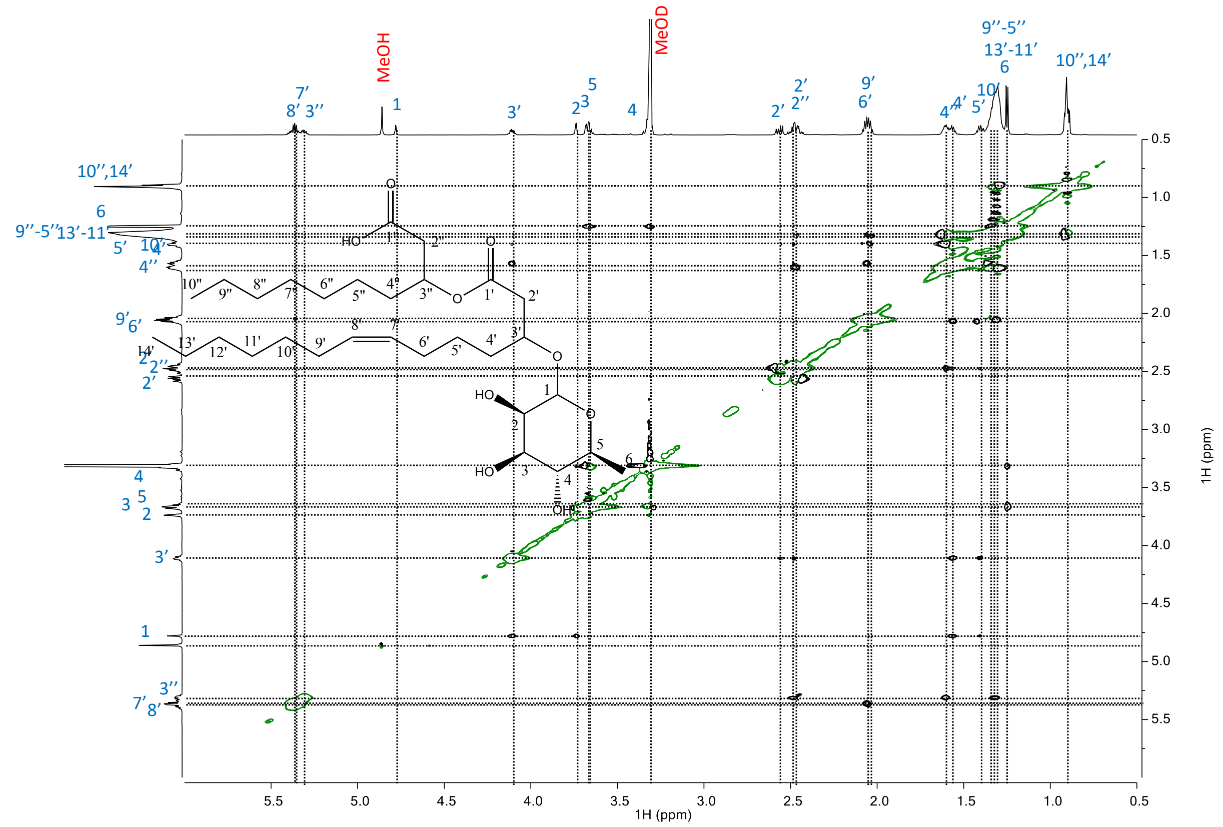

Figure S42. 2D ROESY (300 ms) of **6** in methanol-d4, T=298 K.

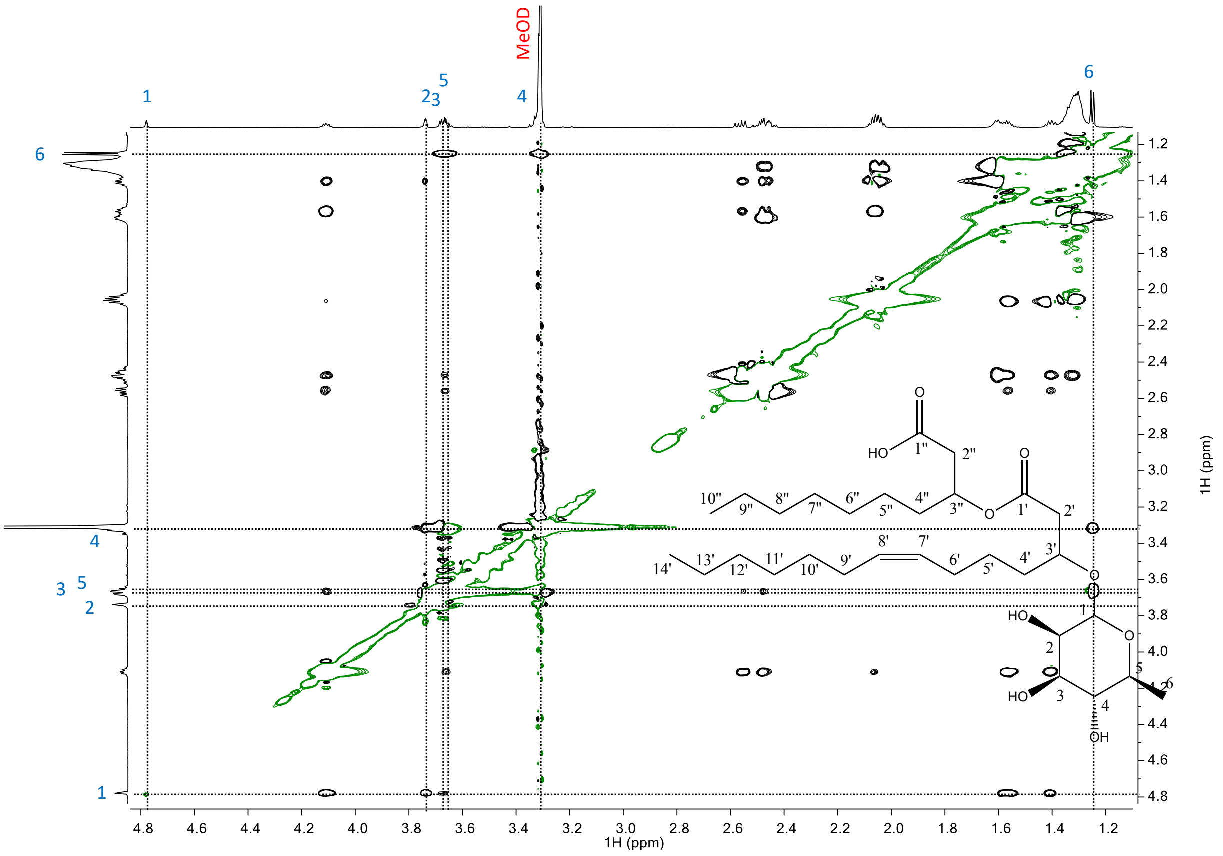

Figure S43. Blown up cyclic region of 2D ROESY (300 ms) of **6** in methanol-d4, T=298 K.

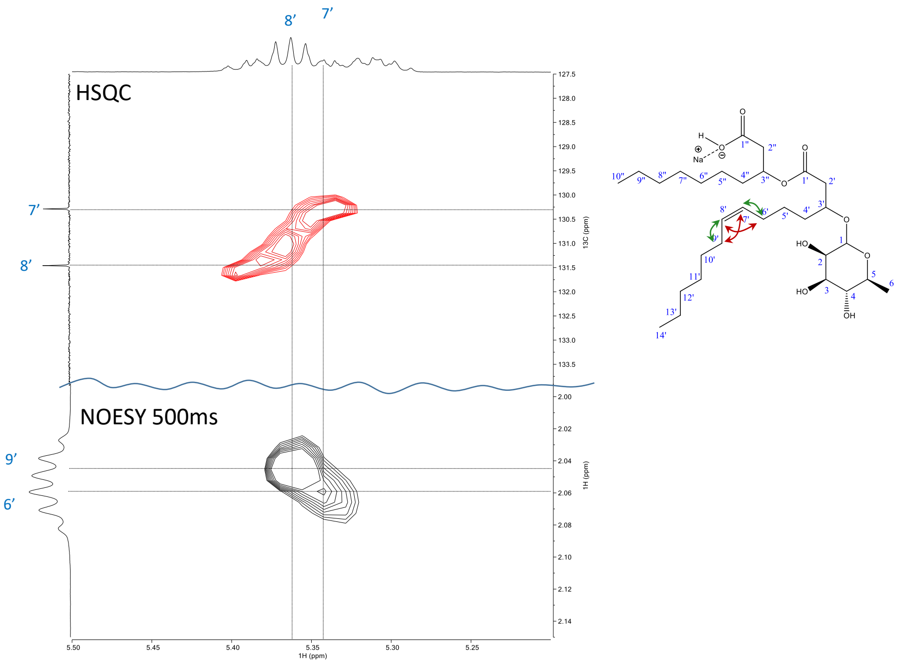

Figure S44. Expansion of the nearly overlapped olefinic protons in a 2D NOESY (500 ms) of **3** in methanol-d$_4$, T=298 K. The absence of NOE6′,8′ and NOE9′,7′ follows the *cis*-pattern observed for 4, which could be reliably determined to be in *cis* configuration.

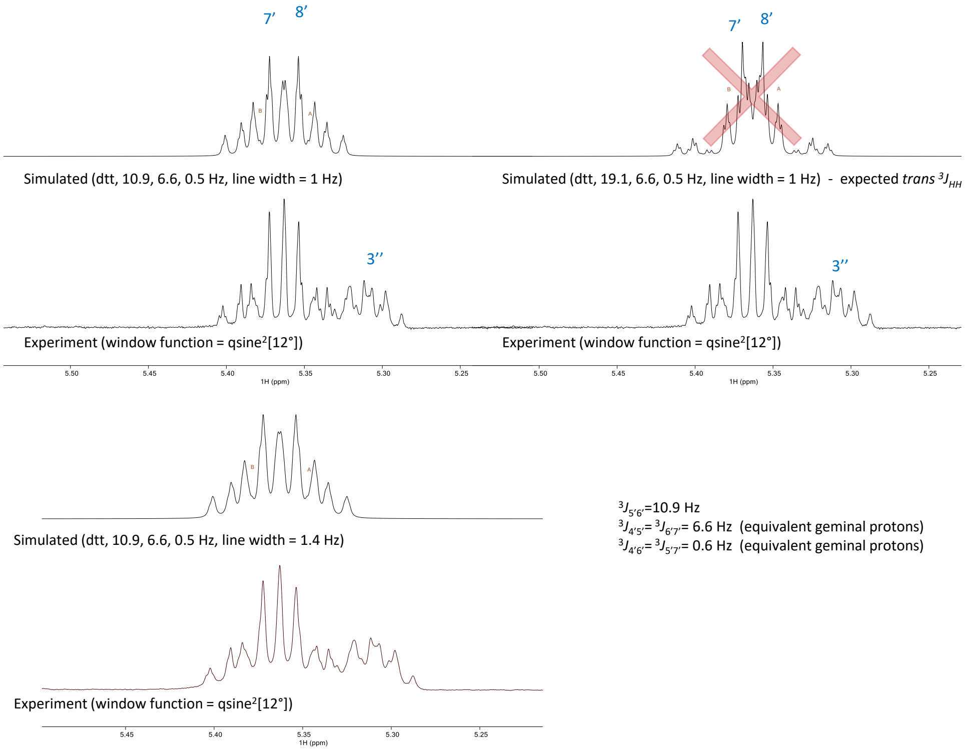

Figure S45. Comparison between experimental and simulated multiplets of the nearly overlapped olefinic protons of **3**. The fit was made using a qsine^2(12°) window function (upper panel), and then compared to the raw multiplets (lower panel). The best fit was found to be a dtt, 10.9, 6.6, 0.6 Hz multiplet, which indicates a cis configuration (expected $^3J_{7'8'}$ ~ 11 Hz). The right panel shows the simulated peak for the expected trans coupling ~19 Hz with shows a poor fit.

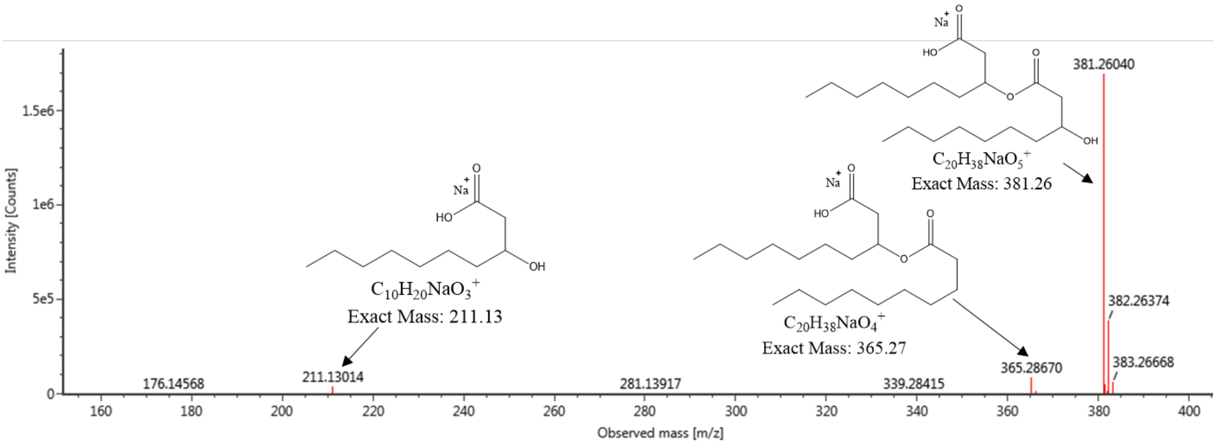

Figure S46. MS/MS spectrum of **1** [M + Na] $^+$. Ion mode ESI+.

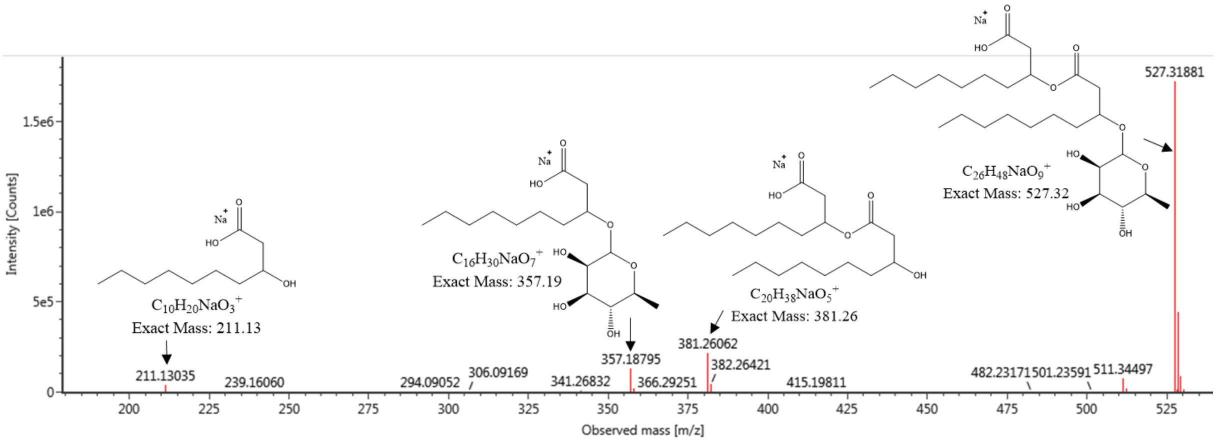

Figure S47. MS/MS spectrum of **2** [M + Na] $^+$. Ion mode ESI+.

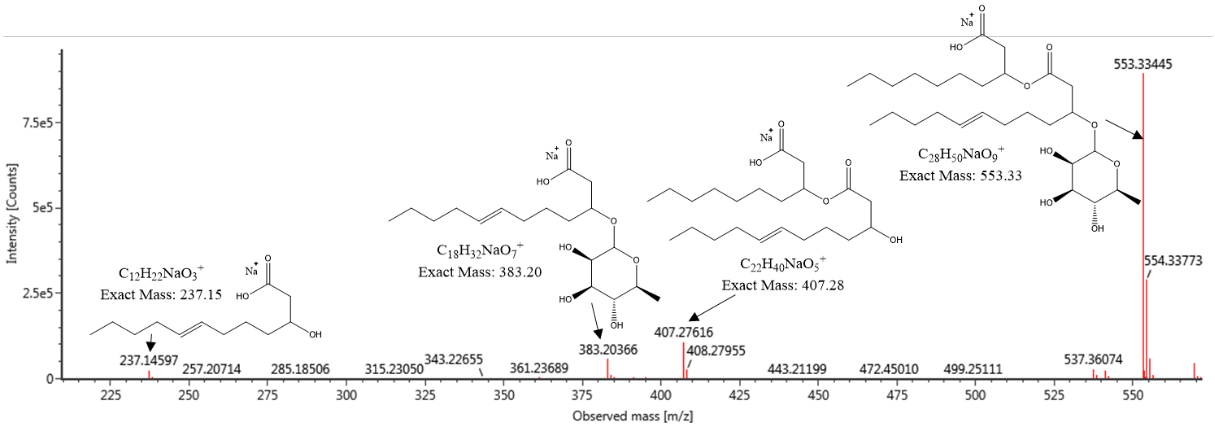

Figure S48. MS/MS spectrum of **3** [M + Na] $^+$. Ion mode ESI+.

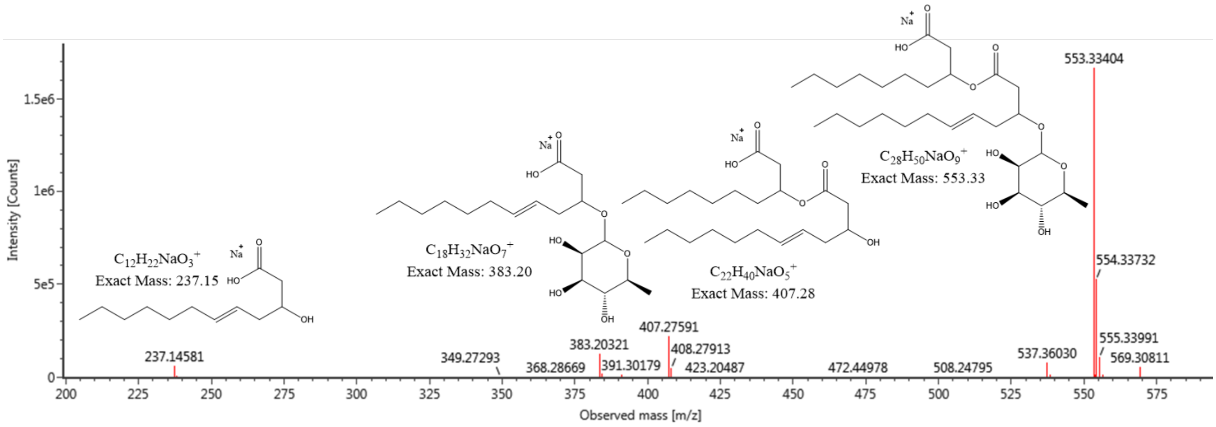

Figure S49. MS/MS spectrum of **4** [M + Na] +. Ion mode ESI+.

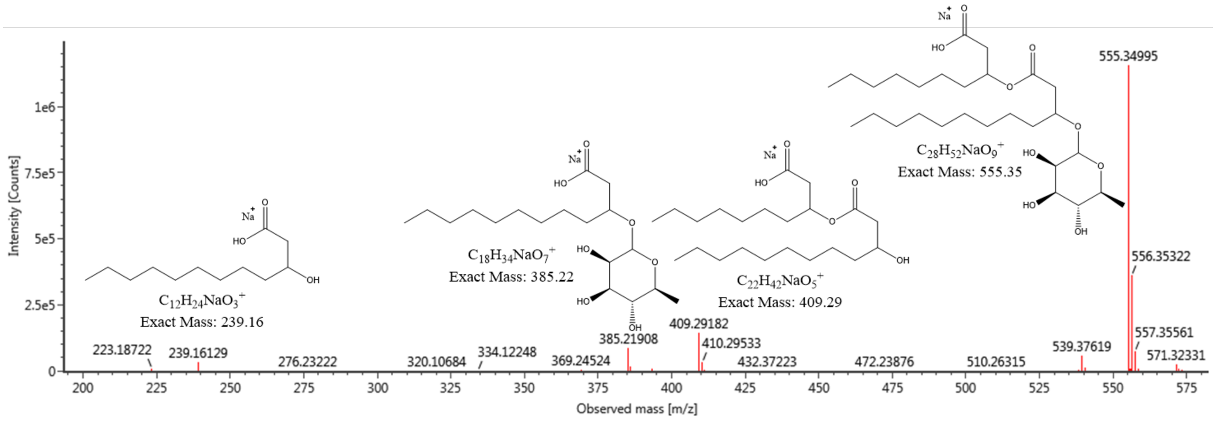

Figure S50. MS/MS spectrum of **5** [M + Na] +. Ion mode ESI+.

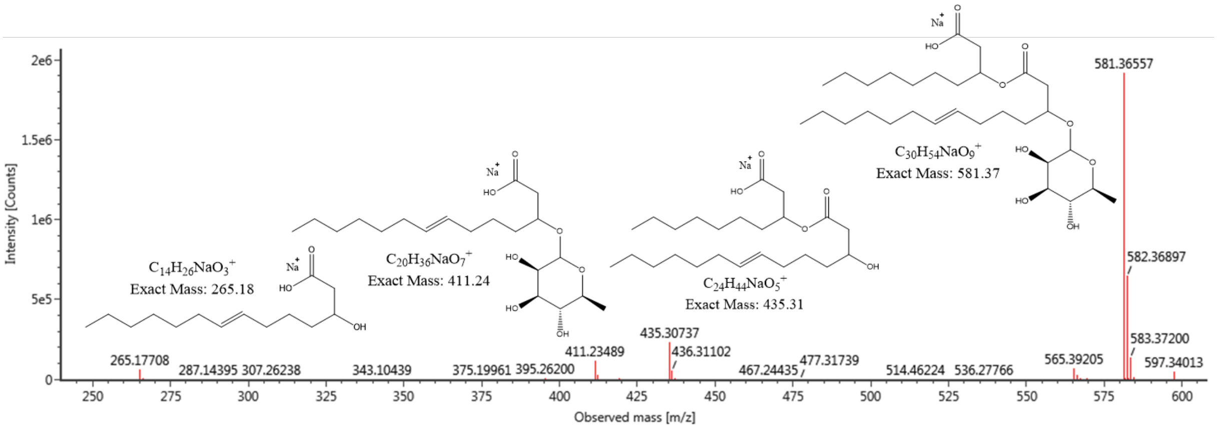

Figure S51. MS/MS spectrum of **6** [M + Na] +. Ion mode ESI+.
